# Supplementary material for: The evolutionary history of 2,658 cancers
Source: Nature. 2020 Feb 5;578(7793):122–8. doi: 10.1038/s41586-019-1907-7 (PMC7054212; doi:10.1038/s41586-019-1907-7)
Supplement: Supplementary file 1 — This file contains a more detailed description of all methods, three supplementary notes, and summary pages for each PCAWG cohort, with sample-level figures representing the results of each of the life history analyses: timing of gains, ordering of events, timing of drivers, signature changes and evolutionary timelines. [file 41586_2019_1907_MOESM1_ESM.pdf]

nature

https://doi.org/10.1038/s41586-019-1907-7

Supplementary information

The evolutionary history of 2,658 cancers

In the format provided by the authors and unedited

Supplementary Information

Supplementary Methods **1**

A more detailed description of all methods.

Supplementary Notes **32**

1. Limitations of single-sample evolutionary timing analyses 32
2. A detailed interpretation of an example cancer timeline 36
3. Data availability 38

Summary pages for all PCAWG cancer types **45**

Summary pages for each PCAWG cohort, with sample-level figures representing the results of each of the life history analyses: timing of gains, ordering of events, timing of drivers, signature changes and evolutionary timelines.

Supplementary Methods

Table of contents

1. The PCAWG dataset 4
2. Timing of copy number gains, point mutations and subclones 5
   2.1. cancerTiming (Fig. 1c) 5
   2.2. MutationTimeR 6
      2.2.1. Model 6
      2.2.2. Parameter estimation 7
      2.2.3. Mutation assignment 8
      2.2.4. Timing of copy number gains 8
      2.2.5. Notes 9
      2.2.6. Confidence intervals 9
      2.2.7. Code availability 9
   2.3. PhylogicNDT SinglePatientTiming (Fig. 3a-d) 10
   2.4. Validation of copy-number timing methods (Extended Data Figure 2) 12
3. Synchronous amplification of large gains (Fig. 1f-h) 13
4. Timing of driver events 14
   4.1. Qualitative timing of driver point mutations (Fig. 2a-d) 14
   4.2. Relative timing of driver mutations (Fig. 3 & Extended Data Fig. 4) 15
      4.2.1. League model relative ordering (PhylogicNDT LeagueModel) 15
      4.2.2. Bradley-Terry model ordering 16
   4.3. Validation of League model relative ordering (Fig. 3 & Extended Data Fig. 4-5) 16
5. Timing of mutational signatures (Fig. 4; Extended Data Figure 9) 17
   5.1. Extracting mutational signature weights from timed mutations 17
   5.2. Testing for spectral changes 18
   5.3. Calculating signature changes 19
6. Real-time estimation of WGD and subclonal diversification (Fig. 5; Extended Data Figure 8-9) 19
   6.1. Mutation types 19
   6.2. Modelling copy number changes 20
   6.3. Subclonal phylogeny 20

6.4. Estimating branch lengths 21
6.5. Selection of samples 22
6.6 Acceleration from relapse samples 22
6.7. Acceleration from mutation burden at diagnosis 24
6.8. Adjusting for mutation rate increase 25
7. Construction of cancer timelines (Fig. 6) 26
8. References 29

1. The PCAWG dataset

This manuscript comprises analyses based on the ICGC-PCAWG dataset, which is fully described in Ref.[1]. Briefly, the dataset contains 2,778 tumours from 2,658 donors, from 38 cancer types (**Supplementary Table 1**). Tumour samples and matched normal samples were whole genome sequenced to a minimum average coverage of 30x and 25x respectively. The majority of samples come from untreated, primary tumours (of which there are 2,605), but a small proportion represent metastases or recurrences (173). Raw SNV and indel calls for each PCAWG tumour were obtained through a consensus of multiple different methods, for which a subset was validated using a deep sequencing approach based on DNA hybridisation capture. Copy number profiles for each sample were generated by the PCAWG Evolution and Heterogeneity working group, based on a consensus of six different copy number callers and structural variant breakpoints as obtained by the PCAWG Structural Variation working group. Also obtained through the Evolution and Heterogeneity working group were subclonal architectures for each tumour, which are the result of a consensus between 11 different methods, and describe the number of subclonal populations per sample, and their size. This study additionally makes use of the annotation of driver gene elements (described as part of Ref.[1]), and the identification of somatic driver mutations in each PCAWG sample[2], both provided by the PCAWG Drivers and Functional Interpretation working group. Lastly, mutational signatures of single-base, double-base and indel mutational processes, as well as their activity in each sample, were produced by the PCAWG Mutational Signatures working group[3]. Two approaches were used to deconvolute mutational signatures across PCAWG. For simplicity in this manuscript we make use of the results of SigProfiler throughout.

Supplementary Table 1: Overview of samples in PCAWG across 38 cancer types, and reference to Supplementary Figures showing results on specific cancer types.

Cancer type	Cohort abbreviation	No. samples	Supp. Fig.
**Biliary adenocarcinoma**	Biliary-AdenoCA	34	7 (p. 40)
**Bladder transitional cell carcinoma**	Bladder-TCC	23	36 (p. 69)
**Bone – benign neoplasm**	Bone-Benign	16	
**Bone – other malignant**	Bone-Epith	10	
**Osteosarcoma**	Bone-Osteosarc	38	26 (p. 59)
**Breast adenocarcinoma**	Breast-AdenoCA	198	8 (p. 41)
**Breast ductal carcinoma *in situ***	Breast-DCIS	3	
**Breast lobular carcinoma**	Breast-LobularCA	13	
**Cervical adenocarcinoma**	Cervix-AdenoCA	2	
**Cervical squamous cell carcinoma**	Cervix-SCC	18	33 (p. 66)

**Glioblastoma**	CNS-GBM	41	15 (p. 48)
**Medulloblastoma**	CNS-Medullo	146	21 (p. 54)
**Oligodendroglioma**	CNS-Oligo	18	25 (p. 58)
**Pilocytic astrocytoma**	CNS-PiloAstro	89	32 (p. 65)
**Colorectal adenocarcinoma**	ColoRect-AdenoCA	60	12 (p. 45)
**Oesophageal adenocarcinoma**	Eso-AdenoCA	98	24 (p. 57)
**Head and neck squamous cell carcinoma**	Head-SCC	57	16 (p. 49)
**Clear cell renal cell carcinoma**	Kidney-CCRCC	111	11 (p. 44)
**Chromophobe renal cell carcinoma**	Kidney-ChRCC	45	9 (p. 42)
**Papillary renal cell carcinoma**	Kidney-PapRCC	33	30 (p. 63)
**Hepatocellular carcinoma**	Liver-HCC	327	17 (p. 50)
**Lung adenocarcinoma**	Lung-AdenoCA	38	20 (p. 53)
**Lung squamous cell lung carcinoma**	Lung-SCC	48	34 (p. 67)
**B-cell non-Hodgkin lymphoma**	Lymph-BNHL	107	6 (p. 39)
**Chronic lymphocytic leukaemia**	Lymph-CLL	95	10 (p. 43)
**Acute myeloid leukaemia**	Myeloid-AML	16	5 (p. 38)
**Myelodysplastic syndrome**	Myeloid-MDS	3	
**Myeloproliferative neoplasms**	Myeloid-MPN	51	23 (p. 56)
**Ovarian adenocarcinoma**	Ovary-AdenoCA	113	27 (p. 60)
**Pancreatic adenocarcinoma**	Panc-AdenoCA	241	28 (p. 61)
**Pancreatic neuroendocrine tumours**	Panc-Endocrine	85	29 (p. 62)
**Prostate adenocarcinoma**	Prost-AdenoCA	286	31 (p. 64)
**Melanoma**	Skin-Melanoma	107	22 (p. 55)
**Leiomyosarcoma**	SoftTissue-Leiomyo	15	18 (p. 51)
**Liposarcoma**	SoftTissue-Liposarc	19	19 (p. 52)
**Gastric adenocarcinoma**	Stomach-AdenoCA	75	14 (p. 47)
**Thyroid adenocarcinoma**	Thy-AdenoCA	48	35 (p. 68)
**Endometrial adenocarcinoma**	Uterus-AdenoCA	51	13 (p. 46)

2. Timing of copy number gains, point mutations and subclones

Three related methods were used to time individual point mutations and copy number gains, which are described in detail below. All three methods are based on the same underlying concept of timing gains using the proportions of co-amplified point mutations.

2.1. cancerTiming (Fig. 1c)

Clemency Jolly

The timing of clonal chromosomal gains may be inferred using the copy number of point mutations within the gained region. Clonal mutations that have occurred before the gain become duplicated along with the chromosomal region, and themselves double in copy number, whereas mutations occurring after the duplication, or on a non-duplicated

chromosome remain in single-copy. Thus, the ratio of single to double-copy mutations gives an estimate of when the chromosomal region was gained in mutational time.

One approach used for the application of this rationale to the 2,658 samples across the PCAWG dataset was a published method, cancerTiming[4], which uses a maximum-likelihood based approach to estimate the timing of single gains (regions of 2+1), double gains (3+1) and copy-neutral loss of heterozygosity (CNLOH, 2+0). Regions of more complex copy number gains are not recommended to be timed using this approach, as the historical copy number of the region cannot be explicitly modelled, which is required to link the observed allele frequencies to the time of the gain. We considered, however, that regions of 2+2 in whole genome duplications are likely to be exceptions to this rule, as we expect that both alleles were gained at the same time. Therefore, the cancerTiming algorithm was modified to accept the input for timing regions of 2+2 in samples that were identified as WGD (the amended function is provided as part of the PCAWG-11 Evolution github repository).

cancerTiming was run with all default parameters except the minimum number of mutations per timed segment, which was lowered to 2. Confidence intervals for mutational time estimates were calculated by taking 500 non-parametric bootstrap samples. Confidence intervals were observed to become very small in cases of low mutation counts, and so were adjusted using the approach of MutationTimeR (described in more detail below).

2.2. MutationTimeR

Moritz Gerstung

2.2.1. Model

We use the following hierarchical model to calculate timing parameters based on copy number and variant allele frequency data. Let X denote the number of reads reporting a variant, n denotes the coverage. The basic model is that mutant read counts follow a beta-binomial distribution.

$$X \sim \mathrm{BetaBin}(n, f, \varrho)$$

Here, f denotes the variant allele frequency, which takes discrete values depending on local copy number and subclonal composition, which we will define in the following. ϱ is a dispersion parameter, which usually takes small values of ϱ=0.01.

Suppose there are s discrete clonal and subclonal states (1 clonal denoted by s=0 and 1, ..., s-1 subclonal states). Using the placeholder S for the unknown state we hence have

$$S \sim \text{Cat}(s)$$

The probabilities for each state s are denoted Pr(s) and are taken as input from the subclonal composition analyses, where Pr(s) = #(s) / # total is the estimated fraction of mutations in a particular state.

Each state allows for c_s different copy number solutions, depending on the major copy number in the given clonal state. For clonal states c_s = 1,..., M where M denotes the major copy number state, as mutations occur initially on a single allele and may be co-amplified with subsequent copy number gains (the total copy number is T=M+m, where m is the number of minor alleles). For subclonal states this implies c_s = 1 in the absence of a subclonal mutation copy number change, and c_s = 1, ..., d for cases with subclonal copy number change as point mutations can be co-amplified or deleted. Here d denotes the difference between ancestral and derived copy number state, which either occurs on the major or minor allele. Values of d are usually -1,+1, denoting subclonal single loss, or gain.

Hence the number of alleles carrying a point mutation is

$$C \mid S \sim \text{Cat}(c_s)$$

Lastly the VAF corresponding to a given state c_s and s is given by f(c_s, s) = $f_s\, c_s$ / ($f_0\, T$ + (1-f_0) N). f_0 denotes the tumour purity, and N is the normal copy number at the given locus (usually 2 for autosomes and 1 or 2 for the allosomes). For loci with subclonal copy number change, T is the weighted average of the total copy number of the two states.

Lastly, a mutant allele may not be detected. We use the assumption that typically 3 reads are required to detect a variant. This has consequences as typically for low VAF fewer mutations will be observed to a lesser extent. Thus, our Y observations will be

$$Y \mid X = X \text{ if } X > 3, \text{ else absent}$$

Hence Y is given by a truncated beta-binomial distribution.

2.2.2. Parameter estimation

The only unknown parameters in the model are P(C | S), which are estimated by an EM-algorithm. P(S) are input from subclonal consensus.

Using Bayes' formula, we have

$$P(C \mid S, Y) = P(C, S, Y) / P(S, Y) = P(Y \mid C, S)\, P(C \mid S)\, P(S) / \Sigma_C\, P(Y \mid C, S)\, P(C \mid S)\, P(S)$$

This implies iteratively calculating $P(C \mid S, Y)$ for all observations Y and taking $P(C \mid S)$ as the average over all Y in each iteration.

Lastly, the probability $P(Y \mid C, S) = P(X \mid C, S) / P(X < 3 \mid C, S)$. Here, $P(X < 3 \mid C, S)$ denotes the power to detect all variants for state C and S, which we decompose into $P(X < 3 \mid C, S) \cong P(X < 3, C \mid S)\, P(X < 3 \mid S) =: \mathrm{Pow}(C \mid S)\, \mathrm{Pow}(S)$, relating to the power of detecting mutations for a particular mutation copy number C for a given state S and the power of each subclonal states S, respectively. These can be readily evaluated from the formulae above for each CN segments for $\mathrm{Pow}(C|S)$ and across all variants for $\mathrm{Pow}(S)$.

Overall, the EM algorithm for estimating the true proportions amounts to iteratively evaluating, where $P(C \mid S)$ and $P(S)$ are divided by their corresponding power terms,

$$P(C \mid S, Y) = P(X = Y \mid C, S)\, P(C \mid S) / \mathrm{Pow}(C \mid S)\, P(S) / \mathrm{Pow}(S) / \mathrm{const.}$$

2.2.3. Mutation assignment

Individual point mutations are assigned a mutation copy number and subclonal state using the MAP estimates

$$c,s = \arg\max P(C, S \mid Y)$$

2.2.4. Timing of copy number gains

The quantities $P(C \mid S = \text{clonal}) =: \pi_C$ denote the unbiased proportions of mutations in a given copy number state C. In cases of copy number gains, these parameters carry important information about the timing of the amplification.

As previously described in Refs.[4,5], the timing of a gain can be expressed by the fraction of co-amplified mutations, accounting for the number of available alleles.

Mono-allelic gains. The general formula for the timing of the first mono-allelic gain on a segment with total copy number $M+m$ and minor copy number m in {0,1} is

$$t_1 = (M+m)\, \pi_M / \textstyle\sum_{i=1}^{M} i\, \pi_i$$

The expression for the latency of the second gain for $M > 2$, m in {0,1} is:

$$t_2 = (M+m)\, \pi_{M-1} / \textstyle\sum_{i=1}^{M} i\, \pi_i$$

Note that tertiary gains cannot be unambiguously timed.

Bi-allelic gains. Bi-allelic gains on both copies, that is M=2, m=2 can be timed similarly, assuming synchronous duplications. Here the formula reads:

$$t_1 = ½\ (M+m)\ \pi_M \,/\, \sum_{i=1}^{M} i\ \pi_i$$

On segments with M=3, m=2, the timing of the first synchronous gain is

$$t_1 = (M+m)\ \pi_M \,/\, \sum_{i=1}^{M} i\ \pi_i$$

and that of the second gain reads

$$t_2 = (M+m)\ (\pi_M - \pi_{M-1}) \,/\, \sum_{i=1}^{M} i\ \pi_i$$

Using the estimated proportions π_c, unlike the number of mutations in a given state, has the advantage of implicitly adjusting for stochastic fluctuations, overlapping subclonal states and power.

2.2.5. Notes

1. The formulae for bi-allelic gains assume that the two alleles are amplified synchronously; this is very plausible in cased of whole-genome duplications, but not guaranteed.
2. In cases of subclonal copy number gains, we calculate the above formula for the ancestral copy number state, summing up those π_c for those c corresponding to the same ancestral state.

2.2.6. Confidence intervals

We use b = 200 bootstraps to calculate confidence intervals [$t_{lo,}$, t_{up}] for the timing estimates t. We observed empirically that these are too narrow for cases of low counts; therefore we use the following weighted average:

$$t_{up,adj} = (5 + n\ t_{up}) \,/\, (5 + n)$$

$$t_{lo,adj} = n\ \ t_{lo} \,/\, (5 + n)$$

Where n is the number of mutations in the given segment used for timing.

2.2.7. Code availability

Code for MutationTimeR is freely available at http://github.com/gerstung-lab/MutationTimeR

2.3. PhylogicNDT SinglePatientTiming (Fig. 3a-d)

Ignaty Leshchiner and Daniel Rosebrock

For each tumour sample, somatic events can be timed relative to one another with different certainty. Subclonal events occur in a subpopulation of cells, and thus occur at a later point in tumour development than clonal events, which occur in all cancer cells in the population. The likelihood that an event is clonal or subclonal is taken into account. In the event of copy number changes in the tumour genome, it is also possible to time clonal events overlapping these copy number altered regions. For a given clonal mutation lying in a gain region with alt_count = k and coverage = n, we compute the likelihood for each mutational copy number (multiplicity) mode as follows:

$$L(\text{mult}) = B(k; n, \text{expected\_af}(\text{mult})),$$

where B(k;n,p) is the probability mass function of the binomial distribution, and expected_af(mult) = mult × purity / (2(1 - purity) + N × purity), and N is the total number of tumour allelic copies in that region. If mutational multiplicity is 1 in a region where both alleles are gained, or one allele is deleted and the other is gained, then the mutation occurred after the copy number gain, if more than 1 then it occurred before the gain. In some instances, it is not possible to accurately time the mutation without phasing information, for example, in regions where only one allele is gained and the other allele retains its single copy, and the mutation has estimated multiplicity of 1, though it is possible to probabilistically assign it to each of the alleles. The multiplicity likelihoods are propagated into the relative timing model.

Using multiplicity rates, clonal copy number gains can be timed in mutational time and relative to one another. We define the quantity π as the relative time of occurrence of a copy number gain with respect to the rate of clonal mutation accumulation. An estimate of $\pi = 0$ signifies a very early gain (all clonal mutations occurring within the gained region occurred after the gain), while an estimate of $\pi = 1$ signifies a very late gain (all clonal mutations occurring within the gained region occurred before the gain). A detailed example of the algorithm for at least one doubled chromosome in a region is described below. First, a prior is defined on the probability of a detected clonal mutation to have multiplicity 2 in a given region, which we define as p_2.

Single allelic gains:

$$p_2 = \pi / (3 - \pi)$$

Double allelic gains or CNLOH:

$$p_2 = \pi / (2 - \pi), \text{ etc.}$$

Assuming π has a uniform prior distribution in [0,1], for regions of single allelic gains, our prior on $p_2 = 3 / (p_2 + 1)^2$, and for regions of double allelic gains or CNLOH, our prior on $p_2 = 2 / (p_2 + 1)^2$.

For a detected clonal mutation with alt count = k, and coverage = n, the likelihood (without a detection power correction) of that mutation having multiplicity 1 or 2 will be:

$$L(mult_1) = B(k; n, expected\_af(mult1))$$

$$L(mult_2) = B(k; n, expected\_af(mult2))$$

Then, for each mutation i, we build a posterior probability of the specific mutation having multiplicity 2, which we define as $f_i(p)$, where p in [0,1],

$$f_i(p) = P_i(mult_1) \times p + (1 - p) \times P_i(mult_2),$$

where

$$P_i(mult_1) = L(mult_1) / (L(mult_1) + L(mult_2))$$

and

$$P_i(mult_2) = L(mult_2) / (L(mult_1) + L(mult_2)).$$

To estimate the posterior distribution on the overall quantity p_2 can then be estimated:

$$p_2(p) = prior\_p_2(p) \times \prod_{i=1}^{N} f_i(p)$$

In order to account for different power to detect mutations of different multiplicities in the region of interest, we create an empirical mapping from observed p_2 space into corrected p_2 space by using the genomic region coverage profile and absolute local copy number (**Supplementary Figure 1**).

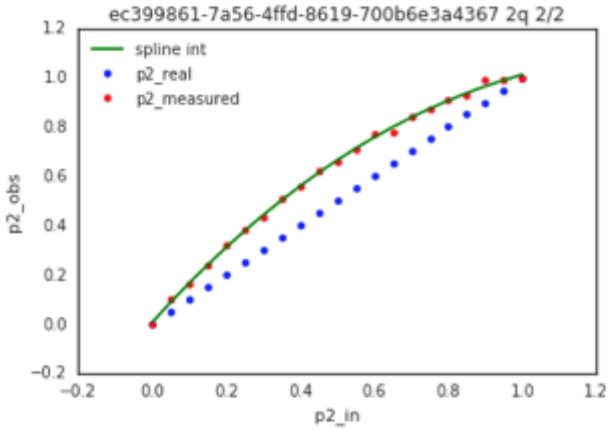

Supplementary Figure 1. Empirical mapping from observed p2 space into corrected p2 space.

Finally, we transform the corrected p_2 to π space via the transformation from above:

Single allelic gains:

$$\pi = 3p_2 / (p_2 + 1)$$

Double allelic gains or CNLOH:

$$\pi = 2p_2 / (p_2 + 1), \text{ etc.}$$

The same procedure was used to time regions of higher allelic copy number and to estimate the timing of whole genome duplication (WGD) events. Whole genome duplications present a unique opportunity to time events across physically disconnected regions of DNA on different chromosomes. Regions of focal or chromosomal full deletion of one allele are more likely to have occurred before a genome doubling event than after. The most likely timeline of events for a region of single allelic copy number is a loss after a whole genome doubling event.

Code availability

Code for PhylogicNDT[6] is freely available at https://github.com/broadinstitute/PhylogicNDT

2.4. Validation of copy-number timing methods (Extended Data Figure 2)

Ignaty Leshchiner and Daniel Rosebrock

We simulated a cohort of samples with random evolutionary trajectories by using the **PhylogicNDT - PhylogicSim TimingSimulator** module. For each simulated tumour sample, we specified a given ordering of somatic events at random times in π space. The clonal

mutation rate for each simulated sample was chosen at random from clonal mutation rates estimated across PCAWG samples. After specifying a clonal mutation rate, mutations were distributed randomly across the genome space. Total bases at risk were updated upon introduction of each copy number event to the genome, as well as multiplicity (number of physical strands of DNA harbouring a point mutation) of each mutation lying in the region affected by the copy number event. By using mutation accumulation across the genome as a molecular clock and correcting for genome-wide tumour ploidy at the end of the simulation, copy number events and driver point mutations were added at their corresponding time in π space. The number of subclones and subclonal mutation rate within each simulated sample was chosen at random from the distribution of subclones and subclonal mutation rates estimated from all PCAWG samples.

The purity of each simulated sample was drawn from the estimated purities of PCAWG samples. We modelled the coverage profiles of whole genome samples by fitting the coverage profiles of PCAWG samples in diploid regions to a beta binomial distribution, the average coverage and the coverage for each simulated mutation was drawn from this distribution, scaling for local ploidy and purity accordingly. The alt count for each simulated mutation was then drawn from a binomial distribution, with the expected allele fraction for that mutation equal to (ccf) × (multiplicity) × (purity)/(2 × (1-purity)+(ploidy) × (purity)). Thus, we have simulated a realistic set of tumour samples with the ordering information preserved.

We then evaluated the 3 copy-number gain timing methods against each other on the PCAWG dataset and against the truth on the simulated dataset (**Extended Data Figure 2**). All results showed high concordance between each other and with the simulated truth results.

3. Synchronous amplification of large gains (Fig. 1f-h)

Santiago Gonzalez, Moritz Gerstung

To confirm if the presence of patients with several chromosomal amplifications co-occurring in a narrow period of time is just a simple random effect or corresponds to an underlying process, we have analysed patients classified as carrying whole genome duplications and those not carrying whole genome duplications. Duplication of the genome is a well-studied single catastrophic event that can be used as a positive control of our analysis.

For each amplified fragment, the relative timing is obtained using MutationTimeR as described above. As previously discussed, the timing confidence intervals depend on the number of mutations present in each fragment, and we have arbitrarily classified as uninformative those with CI (t_{up} - t_{lo}) > 0.5. Similarly, patients with the mean of their CI > 0.5 have been classified as uninformative and excluded from the synchronous analysis. Afterwards, using the values of the individual segments, we estimated the gain timing as the period where most of the observed fragments overlap. Then, we considered the sample as synchronous if 80% of its amplified genome can be explained in just one gain time.
The expected background distribution for the timing of individual gains was obtained through permutations of chromosomes between patients of the same tumour type, with near-diploid tumour genomes. This allowed us to control for the overall differences in the timing of gains between tumour types. The process described above was then applied to the permuted samples.

4. Timing of driver events

4.1. Qualitative timing of driver point mutations (Fig. 2a-d)

Santiago Gonzalez

In order to study the preferred timing when mutations in known driver regions occur, we analysed the list of mutations affecting driver regions provided by the PCAWG Drivers and Functional Interpretation working group[1,2].

According to our previous analysis, we classified mutations in 4 different timing stages: early and late clonal, clonal (NA), and subclonal using MutationTimeR as described above. These 4 states produce 2 different transitions to analyse: (i) early/late referred if the mutation occurred preceding or after the copy number gains, and (ii) clonal/subclonal based on if the mutations is present in all tumour cells or only in a fraction of them.

We merged both substitutions and small indels for the analysis since their timing distribution agree across the different tumours and an independent analysis has shown compatible results performing the analysis separately.

For each of the 50 more mutated driver regions we selected those patients carrying mutations in the analysed locus. For each selected sample, the background is obtained using all the mutations present in fragments with the same copy number configuration as the one carrying

the driver mutation. In order to assess the variability of the estimations we bootstrapped each subgroup 1,000 times.

Because mutations in *TP53* are present across different tumour types, we performed exactly the same analysis on the cited gene but decomposing the patients per tumour type.

4.2. Relative timing of driver mutations (Fig. 3 & Extended Data Fig. 4)

4.2.1. League model relative ordering (PhylogicNDT LeagueModel)

Ignaty Leshchiner, Daniel Rosebrock and Gad Getz

Let $\{x_i\}$, i = 1,...,N be a collection of N somatic mutations and copy number events found in a given sample. For each pair of events, (x_i,x_j), likelihoods of relative ordering of two events are estimated according to procedure described above in 2.3. When two events co-occur across M samples in the cohort, a discrete background multinomial distribution for the event pair, (x_i,x_j) ~ (p1, p2, p3) is formed, where:

p1 = P(x_i before x_j) = probability with x_i before x_j across cohort / M

p2 = P(x_j before x_i) = probability with x_j before x_i across cohort / M

p3 = P(order of (x_i,x_j) unknown) = probability with unknown timing across cohort / M.

For two events which co-occur less than 5 times across the cohort, we increase the uncertainty of the above distribution by contributing additional equal distributed density to p1, p2, p3.

Significant arm level copy number events for the corresponding cohort were included whenever available[7], as well as the 15 most prevalent significant coding or non-coding mutations specific to that cohort whenever available[8]. Only events that occurred in at least 3 samples across the cohort and had a prevalence of at least 5%, were included in the final events for the league model. Similarly, the 5 most focal gains and losses, drawn from known significant focal events specific to that cohort whenever available (or otherwise from pan-cancer significant focal events[9]) were included in the final events for the league model.

The league model is organized into seasons. Within each season, each event "plays" each other event once. Each "game" is played by drawing from the multinomial distribution formed as described above for each event pair. If a win is drawn (event A before event B), then the winner (event A) is awarded 2 points and the loser (event B) 0 points. If an unknown ordering is drawn, then both events are awarded a single point. At the end of the season, the total score is recorded

for each event. A distribution of orderings for each event is made by playing at least 1,000 seasons. This approach is in effect sampling the true underlying joint distribution of the ordering of events across the cohort **(Fig. 3a)**.

In order to detect multi-modal orderings, potentially a result of various subtypes within a cohort with different underlying disease progression models, and to account for outlier samples, we subset to 70% of samples across the cohort at random and multiple league model runs are performed on each of these subsets. The final timing probability density distribution for each event is then integrated across league model runs over all subsets. The method was comprehensively validated on simulated ordering data before being applied to real sequenced cohorts.

Code availability

Code for PhylogicNDT[6] is freely available at https://github.com/broadinstitute/PhylogicNDT

4.2.2. Bradley-Terry model ordering

Tom Mitchell

Contingency tables, collated from the timing estimates of common somatic and copy number events within single samples across each tumour type for were input into an implementation of the Bradley-Terry model of pairwise comparison. A score of 1 was used for wins between event pairs, with no score allocated for draws. Bias reduced maximum likelihood ratios estimated the ability or overall order of each individual contest. Spearman's rho was calculated for the association between the ordering derived from the League model and Bradley-Terry ordering models, with good concordance (**Extended Data Fig. 5**).

4.3. Validation of League model relative ordering (Fig. 3 & Extended Data Fig. 4-5)

Ignaty Leshchiner and Daniel Rosebrock

For the purpose of validating the League model relative ordering methods and results we used PhylogicNDT - PhylogicSim TimingSimulator (see **section 2.4**) to obtain cohorts of simulated samples according to predetermined trajectories, and then ran PhylogicNDT SinglePatientTiming and PhylogicNDT LeagueModel on the simulations. We first validated

that samples simulated from randomly ordered trajectories will give a result where odds of events being early/late in the trajectory is centred on 1 (as expected).

Further, we simulated cohorts of samples generated from a single predefined trajectory (with varied events prevalence). The results showed very high concordance with the true trajectory, fully recovering the expected order (**Extended Data Fig. 4**).

It is expected that a cohort of real tumour samples will have a mixture of distinct trajectories, with some events potentially showing unspecific timing (appearing during different phases of tumour development). To simulate such a scenario, we simulated cohorts of samples coming from a mixture of 2 or more trajectories with varied trajectory prevalence and varied prevalence of constituent events (**Extended Data Fig. 4**). Results showed that the obtained League model ordering result is an average of predefined trajectories (i.e. order of events is consistent with the mixture) with events that are shared between trajectories but have differential order converging to the middle (odds of early vs late of 1) displaying an unspecific order. It is worth noting, that events that were consistently early or late across the trajectories (or absent in some) maintained this predefined early or late order position, confirming our interpretation of results from ordering real PCAWG cohorts.

To quantify the overall accuracy of trajectory reconstruction we simulated a random set of 100 cohorts with random trajectory mixtures and quantifying the distance in odds early/late from perfect ordering (**Extended Data Fig. 4**). We find that in the vast majority of events (even with low number of occurrences in the cohort) the odds error does not exceed 10, suggesting that nearly none of the events would switch between, for example, early timing and middle timing. Most of the events have errors below odds 5 and centred on 0 median error. Mutations, copy gains and copy losses show consistent accuracy profiles (**Extended Data Fig. 4**).

5. Timing of mutational signatures (Fig. 4; Extended Data Figure 9)

Clemency Jolly, Moritz Gerstung, Yulia Rubanova

5.1. Extracting mutational signature weights from timed mutations

Mutations were classified according to the mutational features of the PCAWG Mutational Signatures group[3]. The trinucleotide contexts for all SNVs were obtained from the human reference genome build GRCh37 using Bioconductor package

BSgenome.Hsapiens.UCSC.hg19[10]. Mutations occurring at a purine base were converted to the pyrimidine context to obtain 96 mutational features (as first described in Ref. [11]). Pairs of adjacent SNVs with the same timing classification were classified as doublet base substitutions and also converted to their pyrimidine counterpart where appropriate. For indels, we selected four indel signatures with distinctive mutational features (ID1, ID2, ID8 and ID13), and quantified the number of these features in each sample. For ID1, we took 1bp insertions of T or A, at homopolymer regions of 5+ T's (or A's, respectively). ID2 is similar, although is comprised entirely of deletions of T or A at homopolymer stretches of 5 or more. To quantify ID8, we took deletions of 5+ bp that were not at repeat units, and for ID13 we took deletions of TT (or AA) at 2 bp homopolymer regions of T's or A's. With this catalogue of mutations defined per sample, we then used the timing of SNVs and indels as obtained from MutationTimeR (as described above, section 1.2) to group the mutations into early, late, clonalNA and subclonal. Non-negative least squares was used to estimate the weights of single base substitution (SBS) and doublet base substitution (DBS) signatures. For indel signatures, we simply took the counts of each indel feature in each time frame.

5.2. Testing for spectral changes

We use a likelihood ratio test to assess whether the observed mutation histograms at two different time points differ. Let $\mathbf{X} \in \mathbb{N}_0^{96}$ and $\mathbf{Y} \in \mathbb{N}_0^{96}$ be the trinucleotide single base substitution spectra at two time points, respectively, in a given sample. Assuming that these follow a Multinomial distribution each,

$$\mathbf{X} \sim \text{Mult}(n, \boldsymbol{p}),$$

$$\mathbf{Y} \sim \text{Mult}(m, \boldsymbol{q}),$$

where n and m are the total numbers of single base substitutions at each time point, we calculate a likelihood ratio test for the alternative H_1: $\boldsymbol{p} \neq \boldsymbol{q}$ against the null that the expected spectra are identical H_0: $\boldsymbol{p} = \boldsymbol{q}$. Under the alternative the maximum likelihood estimates are the relative frequencies $\boldsymbol{p} = \mathbf{X} / n$, $\boldsymbol{q} = \mathbf{Y} / m$, respectively, while under the null the estimates are $\boldsymbol{p} = \boldsymbol{q} = (\mathbf{X} + \mathbf{Y}) / (n + m)$. We use the usual χ^2-approximation with 95 degrees of freedom for the deviance $2(l_1 - l_0)$ to calculate p-values.

To account for multiple testing we used the method of Bonferroni to adjust the significance level. Only samples with non-zero mutation counts at both time points were considered informative.

5.3. Calculating signature changes

Proportional signature weights were used to calculate signature changes per sample between early and late clonal mutations, and between clonal and subclonal mutations. The fold change was derived from relative activities (A) as follows, e.g. for early (A_{early}) and late (A_{late}) mutations:

$$\text{fold change} = (A_{late} / (1 - A_{late})) / (A_{early} / (1 - A_{early}))$$

We applied a bootstrapping approach to determine 95% confidence intervals for all of the signature changes. Within each sample, mutations were resampled from their multinomial distributions in early, late, clonalNA and subclonal mutations (where appropriate). Then, the corresponding signature weights and changes were estimated. This process was repeated 1000 times per sample, to generate a distribution of signature changes per signature, from which 95% confidence intervals could be derived. To compute the average signature changes for individual signatures (both pan-cancer and for each cohort), 1000 change estimates were drawn from the corresponding bootstrap replicates, from which a mean change and 95% confidence intervals could be estimated.

6. Real-time estimation of WGD and subclonal diversification (Fig. 5; Extended Data Figure 8-9)

Moritz Gerstung, Santiago Gonzalez

6.1. Mutation types

The logic outlined in the first section calculates the occurrence of events in "mutation time", i.e. the fraction of mutation accumulated over a tumour's lifetime, standardised to the genome size. Hence the estimates *t* are subject to biases resulting from variation in the rate at which mutations accumulate. As a general trend, we can expect the mutation rate to accelerate during tumorigenesis as a consequence of increased proliferation rate and acquired DNA repair deficiencies. The exact rates are often unknown, but recent reports and our own analysis indicates that the accumulation of C>T transitions in a CpG dinucleotide context due to spontaneous deamination of 5meC is a relatively inert process.

Hence, by accounting only for CpG>TpG, one can expect to reduce the influence of more variable mutational processes on the timing estimates. The downside is that this reduces the

number of mutations used for timing to typically only 10-20%, therefore allowing only to time genome-wide events, such as whole genome duplications, or subclonal diversification due to the reduced number of data points. In melanoma samples, the UV spectrum can also generate a considerable number of CpG>TpG mutations, particularly in a CpCpG and a TpCpG context due to the formation of pyrimidine photo dimers upon UV damage. We therefore excluded these two contexts for Skin-Melanoma samples only. A consequence of this restriction is that the estimates of WGD and MRCA shift further away from diagnosis (by about 2-5 years compared to counting all CpG>TpG mutations) and agree better with other cancer types. Applying the same restrictions to all other cancer types did not change the results systematically, but inflated the confidence intervals due to about 50% lower mutation numbers.

Mutations counted: All CpG>TpG, excluding YpCpG for Skin-Melanoma.

6.2. Modelling copy number changes

First, the CpG>TpG burden was adjusted for the DNA content and its changes over time by calculating an effective genome size $G = n \,/\, \sum_i m_i / T_i$, where n denotes the total number of CpG>TpG mutations in a given sample, m_i denotes the estimated multiplicity of mutation i and T_i is the total copy number at this locus. For example, a diploid genome corresponds to $m_i = 1$ and $T_i = 2$ for all mutations, therefore $G = 2$. For a sample with WGD at time 0, all $m_i = 1$ and $T_i = 4$, such that $G = 4$. Conversely, if WGD occurs late, immediately before diagnosis all $m_i = 2$ and $T_i = 4$, such that $G = 2$, indicating that the genome was diploid across the life history of the sample. The advantage of using G at this stage is that it enables to regress out a wide range of ploidy changes. For a more detailed analysis of WGD using only regions of copy number 2+0, 2+1 and 2+2, see below.

6.3. Subclonal phylogeny

The phylogeny of subclones can only be partially resolved using the available data. It is clear though, that subclonal mutations succeed clonal mutations. The main challenge for using the number of subclonal mutations to time the most recent common ancestor (MRCA) stems from the fact that the branches of linearly succeeding subclones are additive, while those of branching clones are not. Hence failure to correctly account for the phylogeny can have an impact on the inferred time. The two extreme scenarios are a *linear* phylogeny and a maximally

branching one. In the latter scenario the expected number of subclonal mutations approximately follows a $1/f$–distribution resulting from the increase of possible subclonal lineages proportional to the number of cells present at different times of clonal expansion. Using a more rigorous population genetic assessment, Noorbakhsh and Chuang (2017)[12] noted that the expected allele frequency distribution in a growing tumour is f^{-k}, where k is the relative selective advantage and k=1 being the case of no selection. Thus, we implicitly assume that the average selective advantage within the cancer is small, $k \approx 1$, and we note that individual samples may deviate from this expectation due to linearly succeeding subclones and/or drift. Nevertheless, the advantage of this approach is that it can be easily applied by scaling the number of subclonal variants at a given frequency f with the inverse of f. The median branch length, scaled by the ploidy is 7%. We use this phylogeny for the analyses shown in **Figure 5**, as it is most conservative (shortest branch lengths).

A linear phylogeny on the other hand increases the subclonal branch length on average by 77% (21%-108% IQR). The corresponding median subclonal branch length would be 14% (compared to 7% under a branching model), showing that the phylogeny has a profound, but also not prohibitive influence on timing the MRCA.

6.4. Estimating branch lengths

For a given branch j (clonal trunk, subclones), we estimate the number of CpG>TpG mutations by summing over the posterior probability $p_{ij} = \mathrm{P}(S=j \mid Y_i)$ and adjusting for the power to detect variants at the expected frequencies, both calculated by MutationTimeR, $n_j = \sum_i p_{ij} / \mathrm{Pow(j)}$. As discussed above, different subclones are folded into a single branch as $n_{subclonal} = \sum_j f_j\, n_j$. We estimate the length of each branch as

$$b_j = n_j / G_j = \sum_j f_j \sum_i p_{ij} / \mathrm{Pow}(j) / G_j$$

Thus, branch lengths (clonal, subclonal) are adjusted for:

- Power to detect variants
- Ploidy changes
- Phylogeny

6.5. Selection of samples

Hypermutation usually leads to mutation of a very characteristic spectrum, such as TpCpT>TpApT mutations in *POLE* mutant tumours. However, mismatch repair deficiency and also *MBD4* mutations increase the rate of CpG>TpG mutations. We therefore removed hypermutant samples from the real-time inferences.

Second, let $b = b_{clonal} + b_{subclonal}$ be the power, ploidy and branching adjusted mutation burden. The average rate of mutation acquisition prior to diagnosis at age a is $\mu = b/a$.

If the hypermutation is acquired during the life history of the sample the change in rate may bias the inferred timing. Noting that hyper mutant samples usually display a dramatic increase in μ, we removed samples j, for which $|\mu_j - \text{median}(\mu_j)| < 2$ in a given tissue.

Another possible source of bias arises from tumour in normal contamination (TiN). As most variant calling algorithms remove variants found in the matched normal, the presence of cancer variants in the normal (usually blood) leads to the loss of some mutations, and especially so for variants at high VAF, as they are more likely to be detected in the normal and subsequently filtered from the cancer sample. To mitigate the possible effect of this bias samples with TiN > 0.01. This criterion was met for 2,101/2,778 samples (253 with TiN > 0.01, 423 with missing values).

Samples j selected:

- Tumour in normal ≤ 0.01 (676 samples removed)
- $|\mu_j - \text{median}(\mu_j)| < 2$ in a given tissue (67 hyper mutant samples removed)
- Sample not cell line (1 sample removed)

6.6 Acceleration from relapse samples

Tumours sequenced at primary and relapse stages allow to compare the number of mutations acquired during the patient's life with the mutations acquired during the relapse period. We have used 8 different tumour types in order to verify the variability of the acceleration in the mutational rate across tumours: 9 ovarian samples from PCWAG, 4 acute myeloid leukemia samples[13], 7 breast cancer samples[14], 2 medulloblastoma[15], 2 liver cancer (TCGA-DD-AACA, TCGA-ZS-A9CF), 4 low grade glioma (TCGA-DU-6397, TCGA-DU-5872, TCGA-DH-

A669, TCGA-FG-5963), 1 lung cancer (TCGA-50-5946) and 1 B-cell lymphoma[16]. The age of the patients ranges from 25 years up to 74, which also confers a wide distribution.

The estimation of the acceleration in each individual patient can be affected by biological and methodological factors: hypermutation processes, changes in the division rate, the existence of various subclones, differences in purity between the primary and relapse sample, *etc*. Thus, our objective is to establish a confident interval where this acceleration is located. We do that, even assuming that the previous mentioned processes can increase the dispersion of the values in a wider interval. These values are used to calculate our maximum and minimum absolute timing estimations in our timing analysis.

In order to do that, we have selected only the clonal mutations present in the primary and the relapse sample. For those samples with copy number information we have modelled 3 different scenarios, which are represented in the supplementary figure using error bars, considering the time of its acquisition: (I) No copy number information as in samples where this data lacks, (II) mutations are acquired prior to the copy number acquisition, (III) copy number is prior to the mutations. The time of divergence (t_d) between the primary and the relapse sample could be prior to the diagnosis, to minimize this effect we have assumed an equivalent mutation rate while both samples coexist. The final mutation rate is estimated as shown in **Supplementary Figure 2**.

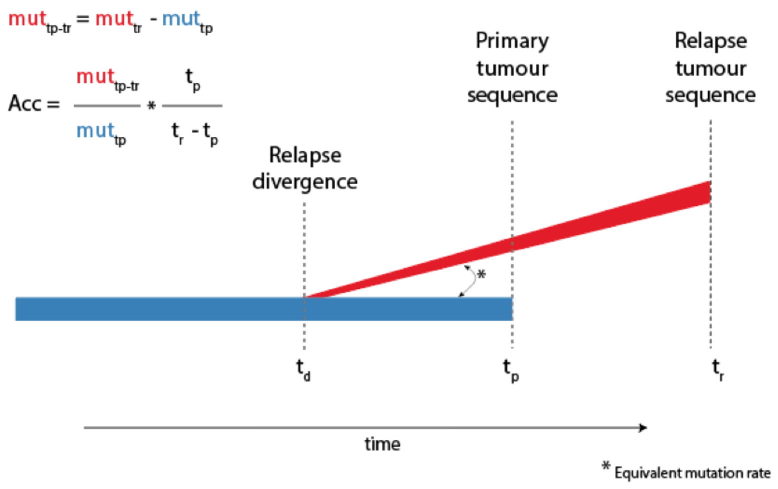

Supplementary Figure 2. Schematic of mutation rate estimation based on primary and relapse samples.

6.7. Acceleration from mutation burden at diagnosis

All somatic mutations arise between zygote and diagnosis and are practically irreversible. Hence the total mutation burden for an individual cancer can only increase over time. Hence, for a given sample the relation between then number of mutations (in retained chromosomal segments) and time is monotonously increasing. If the rate per sample was constant, the relation is linear, if there is a late increase in mutation rate, then the relation is convex. *Assuming that the baseline mutation rate in a given tissue is constant across samples*, the surplus of mutations relative to the linear increase can be estimated by the offset of a linear fit. This logic has been developed and demonstrated to hold across a range of evolutionary models by Tomasetti *et al.* (2013)[17]. Here we apply this logic to CpG>TpG mutations only (see comment above regarding Skin-Melanoma samples).

To study the fraction of mutations attributable to a linear, fixed rate accumulation in a given tissue, we fitted a hierarchical Bayesian model to the mutation burden *b* as a function of age *a* and tissue *t*. The Bayesian model allows to account for offset and slope to be strictly positive and share information across cancer types. The model used is

$$b \mid c, \mu, a \sim \mathrm{N}(\mu a + c, a^2 \tau^2 + \sigma^2)$$

$$\mu \mid t \sim \mathrm{Gamma}(\alpha, \beta)$$

$$c \mid t \sim \mathrm{Gamma}(\delta, \gamma)$$

Here μ denotes the mutation rate in each tumour type and *c* measures the offset per tumour type. Both parameters are linked across tumour types by Gamma distributions to ensure positivity. The variance of the mutation burden has a constant and an age-dependent contribution τ^2, and σ^2. Model parameters and confidence intervals are estimated using Hamiltonian Monte-Carlo[18] as implemented in the rstan package[19] and run over 2,000 iterations after 1,000 burn in steps.

The fraction of mutations *f* contributed by the linear term can be calculated as $f = \mu\ a\ /\ c$ and the mean of this quantity is calculated across all samples of a given tumour type. A confidence interval for *f* is calculated from the joint distribution of μ and *c*.

Results for this analysis part are shown in **Extended Data Figure 8.**

6.8. Adjusting for mutation rate increase

The mutation rate prior to the first sequenced sample has been obtained using the total number of mutations of the primary tumour and the age of the patient. Similarly, the mutation rate between the primary tumour and the relapse consists of the increment of mutations observed in the relapse sample divided by the relapse time. We calculate the acceleration for all observed mutations and in 5meC deaminations. One AML patient has been filtered out during this process due to the relapse sample showing less mutations than the primary tumour which seems to be inconsistent.

From the set of π_i, one can calculate the time points $[0, t_g, t_c, 1]$, where 0 denotes fertilisation, t_g is the time of the first gain and t_c is the time of the most recent common ancestor. In case of an intermittent rate acceleration at time t_a, one has to adjust these estimates accordingly.

Clearly nothing would change if the acceleration occurs at times 0 or 1, as it would influence either all or none of the observed mutations. Generally, an acceleration at later times would inflate the estimate of the period after the acceleration and an adjustment seeks to reverse this inflation. Here, we assume that the acceleration occurs during the clonal period $[0, t_c]$.

As the exact onset is unknown, we simulate different acceleration values a=1,...,10x and average this over the period from $t_a \in [t_0, 1] \times t_c$, where $t_0 = \max\{1\text{-}15\text{yr/age}, 0.5\}$. Note that for a median age at diagnosis of 60yr, t_0 would be 0.75. The rationale of this approach is that any acceleration is expected to occur during the late stages (~25% of clonal molecular time, but not less than 50%) of tumour development.

For the duration of $[t_c,1]$ we use the power- and branching adjusted proportion of all subclonal mutations as discussed above. Hence the acceleration in different periods is

- $[0, t_a] = 1$
- $[t_a, t_c]$ = a (variable t_a)
- $[t_c, 1]$ = a

For the results shown in **Extended Data Fig. 9**, the following acceleration was chosen

- 7.5x for Ovary-AdenoCa and Liver-HCC in agreement with the timing of relapse samples
- 2.5x for CNS-Medullo, CNS-GBM, CNS-Oligo and CNS-PiloAstro in agreement with relapse samples and mutation burden analysis.

Notes

- The approach does not account for the (unknown) time between the emergence of the founder cell of a subclone and diagnosis. Hence it is likely to underestimate the appearance of events by the duration of the subclonal expansion, which can be expected to take several months to a year.
- As shown in **Extended Data Figure 9**, selecting an acceleration value *a* for each sample, based on the adjusted mutation burden *b*, relative to the tissue lower quartile (ie. higher acceleration for those samples with greater burden), does not qualitatively change the estimated median time of occurrence. We thus conclude that the observed variation of inferred timing is not driven by differences in acceleration between samples.
- A total of 818 samples were initially classified as WGD, from which the following number of samples were removed for absolute timing purposes
    - 124 due to abnormal mutation rates
    - 71 were classified as WGD uncertain
    - 13 had no age information

Code

R code for this and other parts of the analysis is available at http://github.com/gerstung-lab/PCAWG-11.

7. Construction of cancer timelines (Fig. 6)

Clemency Jolly

Taken together, these analyses allow us to build a typical picture of tumour development for each cancer type, placing key events along the timeline leading up to diagnosis, and characterising the changing activity of mutational processes. The input to these evolutionary timelines, and how they are combined, is depicted in **Supplementary Figure 3**.

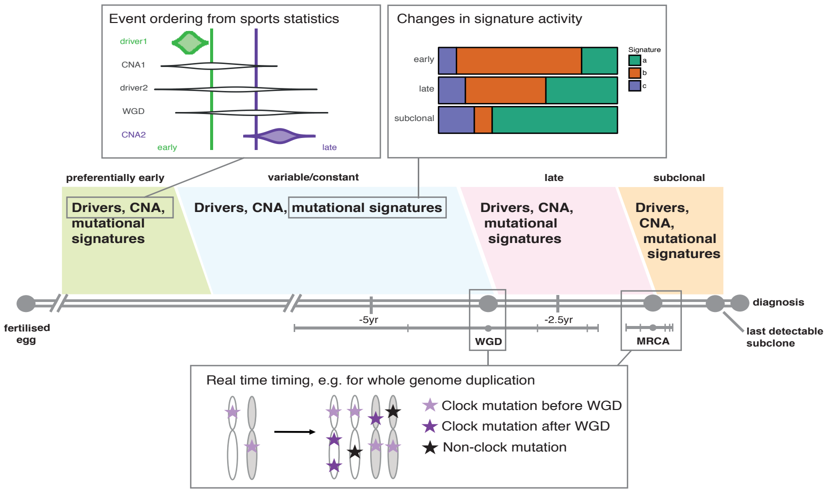

Supplementary Figure 3. A schematic depicting the integration of evolutionary analyses into individual cancer timelines.

Each timeline spans from the fertilised egg to the median age of diagnosis per cohort, although in most cases this axis is broken to allow a clearer visualisation of events in the later stages of tumour evolution. Working back from the point of diagnosis, the median real time estimates of the MRCA, and WGD events, may be placed directly onto the timeline. The real-time points on the timelines are those according to a 5x acceleration of molecular clock, with the exceptions of ovarian adenocarcinoma and the four central nervous system tumours (which were observed from relapse samples to experience an acceleration of 7.5x and 2.5x, respectively).

Between these real time anchor points, it is then possible to interleave the ordered driver mutations and copy number aberrations as provided by the league model (events only shown if present in more than 10% of samples). The first time period, marked as “preferentially early”, comprises events from the league model that have an odds ratio of being early > 10. As we do not know precisely when this interval begins, there is a break in the timeline close to the fertilised egg, and the first epoch starts from there. The subsequent “variable/constant” time period includes events that are assigned a variable timing from the league model, but are ranked before the WGD event. Again, we are unsure precisely where this interval starts, and so it also begins shortly after another break in the timeline. The “late” period does have a definite start, as this includes events which are ranked after WGD, when it occurs. In the final, “subclonal” stage, events are included if they are amongst the last in the league model ranking, and are subclonal in at least 50% of cases.

Signatures are shown on the timeline if they change over time, or if they contribute a substantial fraction of mutations consistently (at least 10% of mutations in one time period). Where there is evidence for a signature change (i.e. confidence intervals not overlapping 0), then the signature is annotated during the epoch of its greatest intensity. Where there is no change, signatures are annotated in the middle "variable/constant" epoch.

Additionally, mutations with known timings during oncogenesis are annotated, for example if they have been described to occur in cancer precursors. Where our timing agrees with the timing reported in the literature, events are annotated "*", where our timing does not agree, the event is denoted with "†". The set of timed mutations from the literature is tabulated in **Supplementary Table 2**.

Supplementary Table 2: Driver mutations in pre-cancers and subclones. Mutations with a known timing from the literature were annotated on the cancer timelines. Here, known events in each tumour type are shown, with accompanying references.

Cancer Type	*Precursor*	*Precursor driver*	*Late*	*Subclonal*	*Note*	*References*
Colorectal-AdenoCa	Adenoma	*APC, KRAS*	*TP53*			[20]
Prostate-AdenoCa	Prostate Intraepithelial Neoplasia; PIN	*FOXA1*, +1q, +8q; *TPRSS-ERG*; -8p	*SPOP, KDM6A, KMT2D*			[21]
Myeloid	Clonal Haematopoiesis	*DNMT3A, TET2, SRSF2, U2AF1*		*NPM1*		[22-24]
Ovary-AdenoCa	STIC	*TP53*				[25,26]
Breast-AdenoCa	Ductal Carcinoma In Situ; DCIS	Unknown			High similarity between different stages	[27]
Pancreatic-AdenoCa	Pancreatic Intraepithelial Neoplasm; PanIN	*KRAS, CDKN2A, BRAF, GNAS*	*TP53, SMAD4*			[28,29]
Esophagous-AdenoCa	Barrett's Esophagous		*TP53, SMAD4,* WGD		Most recurrent genes tend to overlap	[30-32]
Cervix-SCC	Cervical Intraepithelial Neoplasm; CIN	Unknown; HPV virus			The frequency and average number of genetic alterations corresponded directly to the extent to which the cervical carcinoma had progressed.	[33]
Liver-HCC	Hepatocellular Adenoma; HCA	*CTNNB1, TERT; HNF1A* and *IL6ST* only in HCA; HBV and HCV viruses	*TP53*			[34]
Skin-Melanoma	Benign nevus	*BRAF; NRAS* and *TERT* intermediate	*CDKN2A, PTEN, TP53*			[35]
CNS-GBM	Normal brain tissue	+7				[36]
Kidney-RCC		t(3;5)		*PTEN, SETD2, KDM5C*	Timing of t(3;5) by point mutations	[37,38]
Lung-AdenoCa	Atypical Adenomatous Hyperplasia; AAH Adenocarcinoma in situ; AIS	*KRAS, TP53, EGFR*				[39]

8. References

1 The ICGC/TCGA Pan-Cancer Analysis of Whole Genomes Network. Pan-cancer analysis of whole genomes. *Nature* (2019).

2 Rheinbay, E. *et al.* On the discovery of somatic driver events in >2,500 whole cancer genomes. *Nature* (2019).

3 Alexandrov, L. B. *et al.* The Repertoire of Mutational Signatures in Human Cancer. *Nature* (2019).

4 Purdom, E. *et al.* Methods and challenges in timing chromosomal abnormalities within cancer samples. *Bioinformatics* **29**, 3113-3120, doi:10.1093/bioinformatics/btt546 (2013).

5 Nik-Zainal, S. *et al.* The life history of 21 breast cancers. *Cell* **149**, 994-1007, doi:10.1016/j.cell.2012.04.023 (2012).

6 Leshchiner, I. *et al.* Comprehensive analysis of tumour initiation, spatial and temporal progression under multiple lines of treatment. *bioRxiv*, 508127, doi:10.1101/508127 (2018).

7 Mermel, C. H. *et al.* GISTIC2.0 facilitates sensitive and confident localization of the targets of focal somatic copy-number alteration in human cancers. *Genome Biol* **12**, R41, doi:10.1186/gb-2011-12-4-r41 (2011).

8 Lawrence, M. S. *et al.* Discovery and saturation analysis of cancer genes across 21 tumour types. *Nature* **505**, 495-501, doi:10.1038/nature12912 (2014).

9 Zack, T. I. *et al.* Pan-cancer patterns of somatic copy number alteration. *Nat Genet* **45**, 1134-1140, doi:10.1038/ng.2760 (2013).

10 BSgenome: Infrastructure for Biostrings-based genome data packages and support for efficient SNP representation. v. R package version 1.38.0.

11 Alexandrov, L. B. *et al.* Signatures of mutational processes in human cancer. *Nature* **500**, 415-421, doi:10.1038/nature12477 (2013).

12 Noorbakhsh, J. & Chuang, J. H. Uncertainties in tumor allele frequencies limit power to infer evolutionary pressures. *Nat Genet* **49**, 1288-1289, doi:10.1038/ng.3876 (2017).

13 Ding, L. *et al.* Clonal evolution in relapsed acute myeloid leukaemia revealed by whole-genome sequencing. *Nature* **481**, 506-510, doi:10.1038/nature10738 (2012).

14 Yates, L. R. *et al.* Genomic Evolution of Breast Cancer Metastasis and Relapse. *Cancer Cell* **32**, 169-184 e167, doi:10.1016/j.ccell.2017.07.005 (2017).

15 Morrissy, A. S. *et al.* Divergent clonal selection dominates medulloblastoma at recurrence. *Nature* **529**, 351-357, doi:10.1038/nature16478 (2016).

16 Moncunill, V. *et al.* Comprehensive characterization of complex structural variations in cancer by directly comparing genome sequence reads. *Nature Biotechnology* **32**, 1106, doi:10.1038/nbt.3027 (2014).

17 Tomasetti, C., Vogelstein, B. & Parmigiani, G. Half or more of the somatic mutations in cancers of self-renewing tissues originate prior to tumor initiation. *Proc Natl Acad Sci U S A* **110**, 1999-2004, doi:10.1073/pnas.1221068110 (2013).

18 Neal, R. A. MCMC using Hamiltonian dynamics. In: Handbook of Markov Chain Monte Carlo, Chapman & Hall / CRC Press, Chapter 5, pp 113-162. (2011).

19 Stan Development Team. RStan: the R interface to Stan. R package version 2.17.3. http://mc-stan.org/. (2018).

20 Fearon, E. R. & Vogelstein, B. A genetic model for colorectal tumorigenesis. *Cell* **61**, 759-767 (1990).

21 Jung, S.-H. *et al.* Genetic Progression of High Grade Prostatic Intraepithelial Neoplasia to Prostate Cancer. *European Urology* **69**, 823-830, doi:10.1016/j.eururo.2015.10.031 (2016).

22 Genovese, G. *et al.* Clonal Hematopoiesis and Blood-Cancer Risk Inferred from Blood DNA Sequence. *New England Journal of Medicine* **371**, 2477-2487, doi:10.1056/NEJMoa1409405 (2014).

23 Jaiswal, S. *et al.* Age-Related Clonal Hematopoiesis Associated with Adverse Outcomes. *New England Journal of Medicine* **371**, 2488-2498, doi:10.1056/NEJMoa1408617 (2014).

24 Papaemmanuil, E. *et al.* Genomic Classification and Prognosis in Acute Myeloid Leukemia. *N Engl J Med* **374**, 2209-2221, doi:10.1056/NEJMoa1516192 (2016).

25 Eckert, M. A. *et al.* Genomics of Ovarian Cancer Progression Reveals Diverse Metastatic Trajectories Including Intraepithelial Metastasis to the Fallopian Tube. *Cancer Discovery* **6**, 1342, doi:10.1158/2159-8290.CD-16-0607 (2016).

26 Folkins, A. K., Jarboe, E. A., Roh, M. H. & Crum, C. P. Precursors to pelvic serous carcinoma and their clinical implications. *Gynecologic Oncology* **113**, 391-396, doi:10.1016/j.ygyno.2009.01.013 (2009).

27 Bombonati, A. & Sgroi, D. C. The molecular pathology of breast cancer progression. *The Journal of Pathology* **223**, 308-318, doi:10.1002/path.2808 (2011).

28 Bardeesy, N. & DePinho, R. A. Pancreatic cancer biology and genetics. *Nature Reviews Cancer* **2**, 897-909, doi:10.1038/nrc949 (2002).

29 Kanda, M. *et al.* Presence of Somatic Mutations in Most Early-Stage Pancreatic Intraepithelial Neoplasia. *Gastroenterology* **142**, 730-733.e739, doi:10.1053/j.gastro.2011.12.042 (2012).

30 Gregson, E. M., Bornschein, J. & Fitzgerald, R. C. Genetic progression of Barrett's oesophagus to oesophageal adenocarcinoma. *British Journal Of Cancer* **115**, 403, doi:10.1038/bjc.2016.219 (2016).

31 Stachler, M. D. *et al.* Paired exome analysis of Barrett's esophagus and adenocarcinoma. *Nat Genet* **47**, 1047-1055, doi:10.1038/ng.3343 (2015).

32 Weaver, J. M. J. *et al.* Ordering of mutations in preinvasive disease stages of esophageal carcinogenesis. *Nature Genetics* **46**, 837, doi:10.1038/ng.3013 (2014).

33 Umayahara, K. *et al.* Comparative genomic hybridization detects genetic alterations during early stages of cervical cancer progression. *Genes, Chromosomes and Cancer* **33**, 98-102, doi:10.1002/gcc.1215 (2002).

34 Schulze, K. *et al.* Exome sequencing of hepatocellular carcinomas identifies new mutational signatures and potential therapeutic targets. *Nature Genetics* **47**, 505, doi:10.1038/ng.3252 (2015).

35 Shain, A. H. *et al.* The Genetic Evolution of Melanoma from Precursor Lesions. *N Engl J Med* **373**, 1926-1936, doi:10.1056/NEJMoa1502583 (2015).

36 Heim, S. *et al.* Trisomy 7 and sex chromosome loss in human brain tissue. *Cytogenetic and Genome Research* **52**, 136-138, doi:10.1159/000132863 (1989).

37 Gerlinger, M. *et al.* Intratumor heterogeneity and branched evolution revealed by multiregion sequencing. *N Engl J Med* **366**, 883-892, doi:10.1056/NEJMoa1113205 (2012).

38 Mitchell, T. J. *et al.* Timing the Landmark Events in the Evolution of Clear Cell Renal Cell Cancer: TRACERx Renal. *Cell*, doi:10.1016/j.cell.2018.02.020 (2018).

39 Izumchenko, E. *et al.* Targeted sequencing reveals clonal genetic changes in the progression of early lung neoplasms and paired circulating DNA. *Nature Communications* **6**, 8258, doi:10.1038/ncomms9258 (2015).

40 Dentro, S. C. *et al.* Portraits of genetic intra-tumour heterogeneity and subclonal selection across cancer types. *bioRxiv*, doi:10.1101/312041 (2018).

Supplementary Note 1

Limitations of single-sample evolutionary timing analyses

To recapitulate the typical evolutionary history of each tumour subtype, the timing of mutational events and processes is extracted in as much detail as possible from individual samples, and then aggregated across a cohort. It should be kept in mind, however, that in terms of timing, certain samples and certain tumour types can be much more informative than others, due to a variety of both technical and biological factors.

Sample collection

Common mutational events need to be identified within each cohort, so that it may be determined whether these events have a specific pattern of timing. This requires suitably large sample sizes, and ideally, a cohort of tumours that correspond to the same type of disease. The PCAWG dataset comprises WGS data from many different sequencing projects, with varying sample sizes (range 2-327). Across the project, cancer types with fewer than 15 samples were excluded from cohort-specific analyses, but nevertheless, there will be more power to reconstruct a sequence of events in the larger cohorts.

Sampling strategy is also important; comparing primary tumours with metastases, or treated and non-treated samples, risks mixing tumours with different evolutionary dynamics, and confounding the overall picture of tumour evolution. Largely, this dataset is made up of untreated, primary tumours, with the exception of certain cohorts, such as Skin-Melanoma which is almost entirely metastatic, and Myeloid-AML, of which all samples have undergone chemotherapy. Similarly, it may be more difficult to reconstruct a comprehensive pathway of tumour development for cancer types which contain multiple subtypes, such as Breast-AdenoCA.

Detecting and timing genomic aberrations

The genomic aberrations a tumour has acquired over its lifetime may be detected from WGS data. The accuracy of these calls depends on how many reads actually correspond to each position in the tumour genome, determined by the depth of sequencing, tumour purity and

ploidy. The number of reads supporting each mutation could influence our ability to not only to detect mutations, but also to separate clonal from subclonal, or early (present on two or more copies) from late (present on one copy), which is a key step in all of the timing analyses.

In terms of sequencing depth, all samples have a minimum coverage of 30x in the tumour and 25x in the normal, which is typically lower than whole exome or targeted sequencing, for example. However, the breadth of WGS is important here; the timing analyses rely on accurate copy number profiles, and relatively high numbers of mutations, particularly for the mutational timing of gains. This would not be so achievable with targeted or whole exome sequencing. Purity values range from 0.13 to 1 (mean 0.64, median 0.66) and overall tumour ploidy varies between 1.29 and 6.18 (mean 2.38, median 2.00). The interplay between these different factors may mean that there is some variability in the resolution for timing point mutations, which would impact downstream timing analyses, such as the mutational timing of gains, or the league model.

We are confident, however, that both the mutations themselves, and their timing, is largely accurate for the samples in this study. Mutation calls were provided by the PCAWG technical working group, and were validated down to low allele frequencies, whilst clonal and subclonal mutations and CNAs were derived from a high-confidence consensus approach (described in Dentro *et al.*[40]). For the mutational timing analysis, subclonal gains were excluded as this would require co-assignment of mutations and CNAs to subclonal populations.

It is also important to consider the effects of biological factors, such as mutation rate, on the timing analyses. The mutational timing of gains does not depend on the assumption of a constant mutation rate over time; time estimates simply describe the relative ordering between point mutations and chromosomal gains. Regional differences in mutation rate should also not impact time estimates, as long as the differences are maintained over time. If different parts of the genome do experience varying rates of acceleration, then this will skew time estimates across the genome, making them incomparable. One way to examine the effect of regional differences in acceleration is to compare the timing of individual chromosomes that are part of a WGD event. From our analyses, time estimates for single chromosomes in WGD samples cluster tightly around a single point. This would suggest that regional differences in mutation rate over time do not substantially impact mutational time estimates.

Lastly, the real-time analyses are based on the assumption of a patient-specific constant accumulation of CpG>TpG mutations. While the overall rate of CpG>TpG mutations in

cancers appears slightly increased compared to normal cells, its exact temporal evolution in a given sample remains unknown. Nevertheless – unless one assumes dramatic rate increases, at odds with the fairly homogenous CpG>TpG mutation burden in primary cancers without repair deficiency, and with the relative surplus of mutations in relapse samples – WGD, and the driver mutations preceding it, appear to occur several years and in some samples possibly a decade or more before diagnosis.

Tumour biology

Differences in the level of genomic aberrations between tumour types mean that ultimately, we can say more about the evolution of some than others. Particularly, as many of the timing analyses rely on interdependencies between mutations and copy number, it is more difficult to reconstruct the evolutionary history of tumours with few genomic alterations.

The clonal allelic status of point mutations is contingent on sampling and local copy number, and thus has somewhat fluid boundaries, that vary between samples. Early and late clonal time-points are often derived from samples with WGD, which occurs in 30% of cancers. In samples that have not undergone genome doubling, this separation is not so clear. Conversely, near-diploid samples provide greater power to detect subclonal variants. It should be noted that mutations in less than 10% of cancer cells will generally be missed by this analysis. The inference thus represents the evolutionary history up until the point of deep subclonal diversification.

It is therefore, inevitable that the more mutated samples, with more timing information, may dominate the cancer type summary, and that clonal history reconstruction may be better powered in samples with WGD. Furthermore, when point mutations are used to quantitatively time copy number gains, samples with more mutations are likely to have more accurate time estimates, although this is reflected in the size of the accompanying confidence intervals.

It is also possible that certain drivers, CNAs, or large events such as WGD, may set a cancer off on a specific evolutionary trajectory that is not common to all samples in the cohort that don't have this transformative event. As we are presenting the average timeline for each tumour type, this may in fact be a mix of multiple timelines, as discussed above. This mixture of evolutionary histories can be captured in the results of the league model, which will assign events that have a changeable timing across the cohort as "intermediate or variable".

Overall, we aim to characterise the evolutionary history of the *average* tumour from each histological subtype, from the information that we can derive from the samples. The events along this timeline are not necessarily present in every tumour, and it may be that this general profile can be more influenced by samples which have more events that can be timed.

Finally, we may also consider the effects of tissue structure and spatial constraints on somatic tumour evolution. In blood cancers, clonal expansion may theoretically proceed unchecked, but in solid tumours there may be structural constraints that influence clonal dynamics. For example, the emergence and expansion of subclonal populations could be inhibited by spatial limitations. In this case, subclones may remain small in physical size, but should continue to acquire mutations as cells in the lineage grow and divide. These mutations would indicate that while the subclone reflects a small proportion of cells, it has been around for a longer time during tumour evolution. This can be distinguished from a similarly sized subclone which has acquired fewer mutations as it is relatively younger. Thus, while some tumour types may appear to have longer periods of subclonal evolution as a result of such tissue or spatial constraints, it likely reflects true underlying biology. Furthermore, we may expect such influences to vary considerably between tissues. However, we would expect cancers deriving from the same tissue to experience similar environmental constraints. As the overall evolutionary trajectories are determined per tumour type, these should be relatively unbiased.

Supplementary Note 2

A detailed interpretation of an example cancer timeline

An example evolutionary timeline can be seen in the figure below (**Supplementary Figure 4**), which shows the typical trajectory of colorectal adenocarcinoma development.

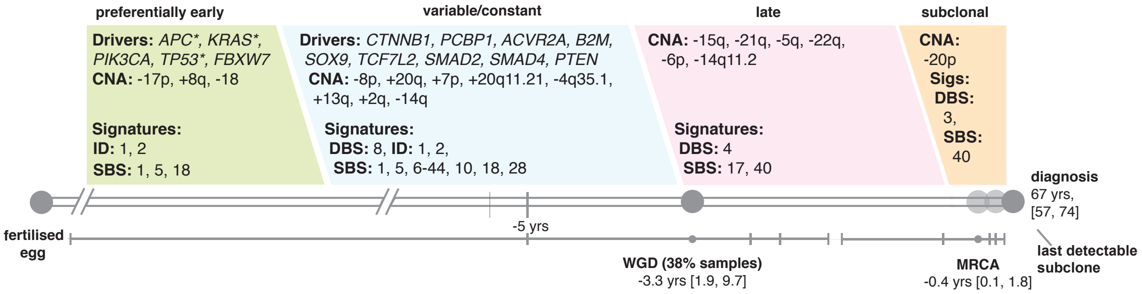

Supplementary Figure 4. The evolutionary timeline of colorectal adenocarcinoma.

The very earliest events, which may indeed play a role in tumour initiation, include mutations in many well-known genes associated with colorectal cancer, including *APC*, *KRAS* and *TP53*. Losses of chromosome 17p, presumably contributing to biallelic inactivation of *TP53*, as well as copy number alterations to 8p and all of chromosome 18 are also identified as particularly early events. In these early phases of somatic evolution, which likely encompass many years or decades of normal tissue maintenance, it is the clock-like signatures which are most active: SBS1 and SBS5, as well as ID1 and ID2, which correspond to small indels generated as a result of slipped strand mispairing during DNA replication. SBS18, considered to represent mutations caused by reactive oxygen species, also appears to be important in the early stages of this disease.

In the variable/constant epoch, there are many events that are estimated to typically occur prior to whole genome duplication. These include driver mutations in multiple cancer genes, and additional copy number gains and losses. In this epoch, we also get a glimpse of mutational signatures that are either particularly active in clonal mutations, or which contribute a substantial proportion to the total mutation burden. Again, the clock-like signatures are influential here, but the impact of other mutational processes on the tumour genome can also be observed, including DBS8 (of unknown aetiology, largely comprised by CA>NN MNVs), single-base substitution signatures 6-44 (which reflect defective MMR), SBS10 (mutant

polymerase epsilon) and SBS28 (of unknown aetiology, but associated with mutant polymerase epsilon, and SBS10).

Whole genome duplication occurs in 38% samples, typically around 3 years prior to diagnosis (median chronological time estimate 3.3 years before diagnosis according to a 5-fold rate of molecular clock acceleration). This value has a substantial range, and the WGD event may be between almost two years before diagnosis, to up to almost a decade prior. Following WGD, there are additional copy number losses across the genome. DBS4 is more active (a signature of unknown derivation), as well as SBS17 (unknown, with a potential link to acid reflux in oesophageal cancer) and SBS40 (unknown aetiology). Typically, losses of chromosome 20 are more associated with subclonal tumour evolution, as are mutations derived from DBS3 (linked to the activity of polymerase epsilon) and SBS40. This subclonal phase typically begins in the last few months before diagnosis.

Supplementary Note 3

Data availability

Supplementary Table 3: Overview of data sets used in this study.

Label	Synapse ID	ICGC DCC URL	ICGC DCC Filename	Access (Open/ Controlled)	Description
**Consensus ICGC SNV+Indel**	syn7357330	http://dcc.icgc.org/releases/PCAWG/consensus_snv_indel/	final_consensus_snv_indel_passonly_icgc.public.tgz	Open	The set of somatically acquired SNVs and indels across PCAWG tumour samples contributed by projects run under the auspices of ICGC. Variant calls were generated by three pipelines run independently on each sample, with subsequent merging into a consensus set of high-quality calls. The file is formatted using the MAF format.
**Consensus TCGA SNV+Indel**	syn7357330	http://dcc.icgc.org/releases/PCAWG/consensus_snv_indel/	final_consensus_passonly.snv_mnv_indel.tcga.controlled.maf.gz	Controlled	The set of somatically acquired SNVs and indels across PCAWG tumour samples contributed by projects run under the auspices of TCGA. Variant calls were generated by three pipelines run independently on each sample, with subsequent merging into a consensus set of high-quality calls. The file is formatted using the MAF format.
**Consensus ICGC SVs (VCF)**	syn7596712	http://dcc.icgc.org/releases/PCAWG/consensus_sv/	final_consensus_sv_vcfs_passonly.icgc.controlled.tgz	Controlled	The set of somatically acquired structural variants across PCAWG tumour samples contributed by researchers in ICGC. Variant calls were generated by three pipelines run independently on each sample, with subsequent merging into a consensus set of high-quality calls. The file is formatted using the VCF format.
**Consensus TCGA SVs (VCF)**	syn7596712	http://dcc.icgc.org/releases/PCAWG/consensus_sv/	final_consensus_sv_vcfs_passonly.tcga.controlled.tgz	Controlled	The set of somatically acquired structural variants across PCAWG tumour samples contributed by researchers in TCGA. Variant calls were generated by three pipelines run independently on each sample, with subsequent merging into a consensus set of high-quality calls. The file is formatted using the VCF format.
**Consensus ICGC CNA (VCF)**	syn8042988	http://dcc.icgc.org/releases/PCAWG/consensus_cnv/	consensus.20170119.somatic.cna.icgc.controlled.tar.gz	Open	The set of somatically acquired copy number alterations across PCAWG tumour samples contributed by researchers in ICGC. Variant calls were generated by three pipelines run independently on each sample, with subsequent merging into a consensus set of high-quality calls. The file is formatted using the VCF format.
**Consensus TCGA CNA (VCF)**	syn8042988	http://dcc.icgc.org/releases/PCAWG/consensus_cnv/	consensus.20170119.somatic.cna.tcga.controlled.tar.gz	Open	The set of somatically acquired copy number alterations across PCAWG tumour samples contributed by researchers in TCGA. Variant calls were generated by three pipelines run independently on each sample, with subsequent merging into a consensus set of high-quality calls. The file is formatted using the VCF format.
**Driver mutational events (ICGC)**	syn11639581	http://dcc.icgc.org/releases/PCAWG/driver_mutations/	TableS3_panorama_driver_mutations_ICGC_samples.controlled.tsv.gz	Controlled	The set of inferred driver mutations in each patient's tumour across PCAWG samples contributed by researchers in ICGC. All classes of somatic mutation are incorporated, including SNVs, indels, somatic mutations and copy number alterations. Drivers are annotated by whether they are coding or non-coding. Both somatic and pathogenic germline variants are reported. The format is a tab-delimited flat text file.
**Driver mutational events**	syn11639581	http://dcc.icgc.org/releases/PCAWG/driv	TableS3_panorama_driver_mutatio	Controlled	The set of inferred driver mutations in each patient's tumour across PCAWG samples contributed by researchers in TCGA. All classes of somatic

**(TCGA)**		er_mutations/	ns_TCGA_samples.controlled.tsv.gz		mutation are incorporated, including SNVs, indels, somatic mutations and copy number alterations. Drivers are annotated by whether they are coding or non-coding. Both somatic and pathogenic germline variants are reported. The format is a tab-delimited flat text file.
**Purity ploidy calls**	syn8272483	http://dcc.icgc.org/releases/PCAWG/consensus_cnv/	consensus.20170217.purity.ploidy.txt.gz	Open	The set of inferred purity and ploidy calls for each patient's tumour across PCAWG samples. Purity values represent the estimated fraction of cells in the sample that are derived from the tumour; ploidy values represent the estimated average copy number of the genome in the tumour cells. Also reported is whether the tumour is predicted to have undergone whole genome duplication. The format is a tab-delimited flat text file.
**Donor clinical data**	syn10389158	https://dcc.icgc.org/releases/PCAWG/clinical_and_histology	pcawg_donor_clinical_August2016_v9.xlsx	Open	Clinical data from PCAWG patients. This dataset includes information on donor demographics (age and sex); treatment, vital status and survival time; smoking history and alcohol history. Note that some of the data for some of the clinical features and risk factors are missing. The format is a spreadsheet.
**Tumor histopathology**	syn1038916	https://dcc.icgc.org/releases/PCAWG/clinical_and_histology	pcawg_specimen_histology_August2016_v9.xlsx	Open	The tumour subtypes were hand-curated and harmonised to icd-0-3 organ system and histological descriptions using a semi-automated process, and then grouped into a series of tiers using a tumour subtype grouping system. This grouping system was reviewed and approved by a group of pathology experts under the coordination of Dr. David Louis at Massachusetts General Hospital. The format is a spreadsheet.
**Mutational Signature activities**	syn11738669	https://dcc.icgc.org/releases/PCAWG/mutational_signatures	PCAWG_sigProfiler_SBS_signatures_in_samples.csv	Open	Mutational signature activities as provided by the PCAWG Mutational Signatures Working Group. Format is Spreadsheet.
**Subclonal architectures - ICGC samples**	syn8532460	https://dcc.icgc.org/releases/PCAWG/subclonal_reconstruction	20170325_consensus_subclonal_reconstruction_beta1.icgc.controlled.tar.gz	Open	Subclonal architectures for every tumour, including the number of clones in each sample, their proportion of tumour cells and mutation assignments. These calls are the result of a robust and conservative consensus constructed out of 11 individual callers via a rigorously validated procedure. The format is reported in a number of tab delimited text files.
**Subclonal architectures - TCGA samples**	syn8532460	https://dcc.icgc.org/releases/PCAWG/subclonal_reconstruction	20170325_consensus_subclonal_reconstruction_beta1.tcga.controlled.tar.gz	Controlled	Subclonal architectures for every tumour, including the number of clones in each sample, their proportion of tumour cells and mutation assignments. These calls are the result of a robust and conservative consensus constructed out of 11 individual callers via a rigorously validated procedure. The format is reported in a number of tab delimited text files.

Supplementary Table 4: Overview of data sets produced by this study.

Label	Synapse File Name	Synapse ID	ICGC DCC Filename	ICGC DCC URL	Access (Open/ Controlled)	Description
**ICGC Timed copy number segments (MutationTime.R)**	2018-07-19-allSegmentsTimeRaw.txt.gz	syn14778989	2018-07-19-allSegmentsTimeRaw.icgc.controlled.txt.gz	https://dcc.icgc.org/releases/PCAWG/evolution_and_heterogeneity	Open	The file contains the following columns:<br>• seqnames chromosome,<br>• start segment start position,<br>• end segment end position,<br>• width segment length,<br>• strand unused segment strand field,<br>• clonal_frequency frequency of the clone carrying the segment (purity if it is the main clone),<br>• total_cn sum of major and minor copy number,<br>• major_cn major copy number,<br>• minor_cn minor copy number,<br>• star copy number classification quality classification,<br>• level copy number category,<br>• n.snv_mnv number of somatic SNV in the segment,<br>• type segment timing classification type,<br>• time molecular time of the gain event,<br>• time.lo, time.up time confidence interval,<br>• time.2nd molecular time of second amplification,<br>• time.2nd.lo, time.2nd.up time second amplification confidence interval,<br>• time.star time classification category,<br>• n.indel number of somatic indels in the segment,<br>• sample sample ID.
**TCGA Timed copy number segments (MutationTime.R)**	2018-07-19-allSegmentsTimeRaw.txt.gz	syn14778989	2018-07-19-allSegmentsTimeRaw.tcga.controlled.txt.gz	https://dcc.icgc.org/releases/PCAWG/evolution_and_heterogeneity	Open	The file contains the following columns:<br>• seqnames chromosome,<br>• start segment start position,<br>• end segment end position,<br>• width segment length,<br>• strand unused segment strand field,<br>• clonal_frequency frequency of the clone carrying the segment (purity if it is the main clone),<br>• total_cn sum of major and minor copy number,<br>• major_cn major copy number,<br>• minor_cn minor copy number,<br>• star copy number classification quality classification,<br>• level copy number category,<br>• n.snv_mnv number of somatic SNV in the segment,<br>• type segment timing classification type,<br>• time molecular time of the gain event,<br>• time.lo, time.up time confidence interval,<br>• time.2nd molecular time of second amplification,<br>• time.2nd.lo, time.2nd.up time second amplification confidence interval,<br>• time.star time classification category,<br>• n.indel number of somatic indels in the segment,<br>• sample sample ID.
**Real time inferences of MRCA and WGD**	2018-07-24-wgdMrcaTiming.txt	syn14778990	2018-07-24-wgdMrcaTiming.txt	https://dcc.icgc.org/releases/PCAWG/evolution_and_heterogeneity	Open	To establish a chronological timing estimate of the most recent common ancestor (MRCA) and whole genome duplications (WGD), only CpG>TpG mutations, which occur in nearly all tissues, were used and corrected to a possible mutation rate increase.<br><br>The file contains the following columns<br>• uuid sample ID,<br>• icgc_sample_id ICGC sample ID,<br>• icgc_donor_id ICGC donor ID,<br>• tissue tumour type,<br>• WGD true/false the sample is carrying a whole genome duplication,<br>• ploidy total ploidy of the sample,<br>• eff_ploidy effective ploidy,<br>• purity purity of the sample,<br>• age patient's age,

						• n_snv_mnv number of somatic SNV,<br>• CpG_TpG_trunk_pwradj number of clonal C>T in CpG sites adjusted,<br>• CpG_TpG_subclonal_branch_pwradj number of subclonal C>T in CpG sites adjusted in the main subclone,<br>• CpG_TpG_subclonal_linear_pwradj number of subclonal C>T in CpG sites adjusted,<br>• TiN Tumour in normal value (samples with TiN > 0.1 are removed),<br>• remove indicator if sample has been retained for chronological timing analysis,<br>• accel estimated acceleration used in the prediction,<br>• WGD.time time of whole genome duplication before diagnosis,<br>• WGD.time.10%, WGD.time.90% confidence intervals of WGD time,<br>• WGD.CpG_TpG_total total number of CpG>TpG mutations in 2+0, 2+1, and 2+2 segments used for chronological timing analysis,<br>• MRCA.time.branching time of most recent common ancestor before diagnosis,<br>• MRCA.time.branching.10%, MRCA.time.branching.90% confidence intervals for the MRCA,<br>• MRCA.time.linear time of most recent common ancestor before diagnosis assuming linear evolution,<br>• MRCA.time.linear.10%, MRCA.time.linear.90% confidence intervals for the MRCA assuming linear evolution.
**ICGC Timed copy number segments (CancerTiming)**	2018-07-25-allSegmentsTime.txt	syn14778991	2018-07-25-allSegmentsTime.icgc.controlled.txt	https://dcc.icgc.org/releases/PCAWG/evolution_and_heterogeneity	Open	The file contains the following columns:<br>• samplename is the tumour whole genome sequencing aliquot identifier,<br>• chromosome indicates the chromosome on which the gain is located,<br>• start and end correspond to the breakpoints of the segments,<br>• time is the mutational time estimate (corresponding to the proportion of mutations before the gain, a number between 0 and 1),<br>• time_lCI and time_uCI are the 95% CI,<br>• no.snvs is the number of mutations used to time the gain, and<br>• type describes the copy number state of the gained segment and can be SingleGain (2+1), DoubleGain (3+1) and CNLOH (2+0).
**TCGA Timed copy number segments (CancerTiming)**	2018-07-25-allSegmentsTime.txt	syn14778991	2018-07-25-allSegmentsTime.tcga.controlled.txt	http//dcc.icgc.org/releases/PCAWG/evolution_and_heterogeneity	Open	The file contains the following columns:<br>• samplename is the tumour whole genome sequencing aliquot identifier,<br>• chromosome indicates the chromosome on which the gain is located,<br>• start and end correspond to the breakpoints of the segments,<br>• time is the mutational time estimate (corresponding to the proportion of mutations before the gain, a number between 0 and 1),<br>• time_lCI and time_uCI are the 95% CI,<br>• no.snvs is the number of mutations used to time the gain, and<br>• type describes the copy number state of the gained segment and can be SingleGain (2+1), DoubleGain (3+1) and CNLOH (2+0).
**Timing of Signature Changes**	2018-07-25-allSignatureChanges.txt	syn14778992	2018-07-25-allSignatureChanges.txt	https://dcc.icgc.org/releases/PCAWG/evolution_and_heterogeneity	Open	For each sample, point mutations were split by their categorical early/late/clonal/subclonal timing. Subsequently the catalogue of active mutational signatures in a given sample (as determined by Alexandrov, et al.) was refit to the mutation spectra in each timing category and fold changes between categories calculated.<br><br>The file contains the following columns:<br>• samplename is the tumour whole genome sequencing aliquot identifier,

						• signature is the PCAWG mutational signature,<br>• wt_clonal, wt_clonalNA, wt_early, wt_late, wt_subclonal correspond to the proportion of mutations attributed to the signature in clonal, clonalNA, early, late, and subclonal mutations,<br>• log2fc_earlyLate and log2fc_clonalSubclonal are the calculated log2 fold changes, after normalising for the proportion of the signature, and<br>• lCI_earlyLate, uCI_earlyLate, lCI_clonalSubclonal and uCI_clonalSubclonal are the corresponding 95% CI for the change.
**ICGC Timing of Driver Gene Mutations**	2018-07-25-driversTiming.txt	syn14954376	2018-07-25-driversTiming.icgc.controlled.txt	https://dcc.icgc.org/releases/PCAWG/evolution_and_heterogeneity	Open	MutationTimeR's output also provides a timing classification for the PCAWG catalogue of driver gene mutations. For each variant an allele frequency estimate was computed and used to establish a qualitative (early/late/clonal/subclonal) timing estimate relative to the local copy number configuration.<br><br>The file contains the following columns:<br>• seqnames chromosome,<br>• start mutation starting coordinate,<br>• ref reference allele,<br>• alt alternative allele,<br>• refDepth number of unmutated reads,<br>• altDepth number of read carrying the alternative variant,<br>• sampleNames donor ID,<br>• sample sample ID,<br>• samples all samples ID from the given donor,<br>• ID mutation unique ID,<br>• MutCN number of segments the mutation is present,<br>• MutDeltaCN Difference between subclonal and ancestral copy number state, if present,<br>• MajCN major copy number of the segment carrying the mutation,<br>• MinCN minor copy number of the segment carrying the mutation,<br>• MajDerCN major copy number of the segment carrying the mutation in the subclone,<br>• MinDerCN minor copy number of the segment carrying the mutation in the subclone,<br>• CNF fraction of read carrying the copy number,<br>• CNID fragment ID the mutation belongs to,<br>• pMutCN probability mutation belongs to the given state,<br>• pGain, pSingle, pSub, probability the mutation belongs to amplified, non-amplified of subclone segment,<br>• pMutCNTail tail probability mutation belongs to the given state,<br>• CLS final timing state assignment.
**TCGA Timing of Driver Gene Mutations**	2018-07-25-driversTiming.txt	syn14954376	2018-07-25-allSegmentsTime.tcga.controlled.txt*	http//dcc.icgc.org/releases/PCAWG/evolution_and_heterogeneity	Controlled	MutationTimeR's output also provides a timing classification for the PCAWG catalogue of driver gene mutations. For each variant an allele frequency estimate was computed and used to establish a qualitative (early/late/clonal/subclonal) timing estimate relative to the local copy number configuration.<br><br>The file contains the following columns:<br>• seqnames chromosome,<br>• start mutation starting coordinate,<br>• ref reference allele,<br>• alt alternative allele,<br>• refDepth number of unmutated reads,<br>• altDepth number of read carrying the alternative variant,<br>• sampleNames donor ID,<br>• sample sample ID,<br>• samples all samples ID from the given donor,<br>• ID mutation unique ID,<br>• MutCN number of segments the mutation is present,

						• MutDeltaCN Difference between subclonal and ancestral copy number state, if present,<br>• MajCN major copy number of the segment carrying the mutation,<br>• MinCN minor copy number of the segment carrying the mutation,<br>• MajDerCN major copy number of the segment carrying the mutation in the subclone,<br>• MinDerCN minor copy number of the segment carrying the mutation in the subclone,<br>• CNF fraction of read carrying the copy number,<br>• CNID fragment ID the mutation belongs to,<br>• pMutCN probability mutation belongs to the given state,<br>• pGain, pSingle, pSub, probability the mutation belongs to amplified, non-amplified of subclone segment,<br>• pMutCNTail tail probability mutation belongs to the given state,<br>• CLS final timing state assignment.
**Mutation Rate Increase**	2018_05_acceleration.txt	syn16780149	2018_05_acceleration.txt	https://dcc.icgc.org/releases/PCAWG/evolution_and_heterogeneity	Open	Estimated mutation rate and acceleration between primary tumours and relapse samples from mutations obtained from different longitudinal studies.<br><br>The file contains the following columns:<br>• ID: ID of the sample for the given study<br>• Ttype: Tumour type the sample belongs to.<br>• Acc_CpG: Acceleration estimation using mutations C>T in CpG sites (used in figure 6c)<br>• Acc_max Acc_min: Error bars for Acc_CpG obtained from mutation bootstrapping. (used in figure 6c)<br>• Primary purity Relapse purity: Purity values of the sample.<br>• Primary_CpG_muts: Number of C>T in CpG site mutations in the primary sample (used in figure 6b)<br>• Relapse_CpG_muts: Number of C>T in CpG site mutations in the relapse sample (used in figure 6b)<br>• shared: Percentage of mutations in the primary tumour also present in the relapse one.<br>• Primary age: Age in years at primary tumour diagnosis.<br>• Relapse age: Period in years between the primary age and the relapse.
**Cancer Timelines**	2018-07-25-timelineInfo.zip	syn14778993	2018-07-25-timelineInfo.zip	https://dcc.icgc.org/releases/PCAWG/evolution_and_heterogeneity	Open	Cancer timelines were generated to summarise the output of the different analysis streams<br><br>The zipped folder comprises the following 5 files:<br>• timelines_ages.txt: This file contains the median ages and IQR per cancer type<br>• timelines_realTimeEstimates.txt: This file contains the median and IQR real time estimates for WGD and MRCA<br>• timelines_leagueModelEvents.txt: This file has a complete list of the events from the league model, the number of times they occur clonally and subclonally per cancer type (columns num_clonal and num_subclonal, the corresponding likelihood of being clonal or subclonal (combined_likelihood_clonal, combined_likelihood_subclonal) and an assignment to one or the other assignment<br>• timelines_sigWeights.txt: This file contains the mean signature weight per cancer type for early, clonal, late and subclonal time periods. mean activity is an average across all time periods used for ranking the signatures on the timelines.<br>• timelines_precursors: A summary of the precursor lesions added to the timelines
**Histology Count Table**	icgc_histology_summary_table.txt	syn14779015	icgc_histology_summary_table.txt	https://dcc.icgc.org/releases/PCAWG/evolution_and_he	Open	An overview of the number of samples per cancer type, split out by the type of tumour (primary, metastasis, etc)

				terogeneity		
**Sample Annotations**	icgc_sample_annotations_summary_table.txt	syn14779014	icgc_sample_annotations_summary_table.txt	https://dcc.icgc.org/releases/PCAWG/evolution_and_heterogeneity	Open	Full set of annotations for each sample, including the number of clonal/subclonal SNVs, indels and SVs, various sample identifiers and high level clinical information such as age at diagnosis, tumour stage and grade and first therapy type and response

Summary Pages for all PCAWG cancer types

A summary of all results obtained per cancer type.

Acute myeloid leukaemia	46
B-cell non-Hodgkin lymphoma	47
Biliary adenocarcinoma	48
Breast adenocarcinoma	49
Chromophobe renal cell carcinoma	50
Chronic lymphocytic leukaemia	51
Clear cell renal cell carcinoma	52
Colorectal adenocarcinoma	53
Endometrial adenocarcinoma	54
Gastric adenocarcinoma	55
Glioblastoma	56
Head and neck squamous cell carcinoma	57
Hepatocellular carcinoma	58
Leiomyosarcoma	59
Liposarcoma	60
Lung adenocarcinoma	61
Medulloblastoma	62
Melanoma	63
Myeloproliferative neoplasms	64
Oesophageal adenocarcinoma	65
Oligodendroglioma	66
Osteosarcoma	67
Ovarian adenocarcinoma	68
Pancreatic adenocarcinoma	69
Pancreatic neuroendocrine tumours	70
Papillary renal cell carcinoma	71
Prostate adenocarcinoma	72
Pilocytic astrocytoma	73
Squamous cell cervical cancer	74
Squamous cell lung cancer	75
Thyroid adenocarcinoma	76
Transitional cell bladder cancer	77

Acute myeloid leukaemia

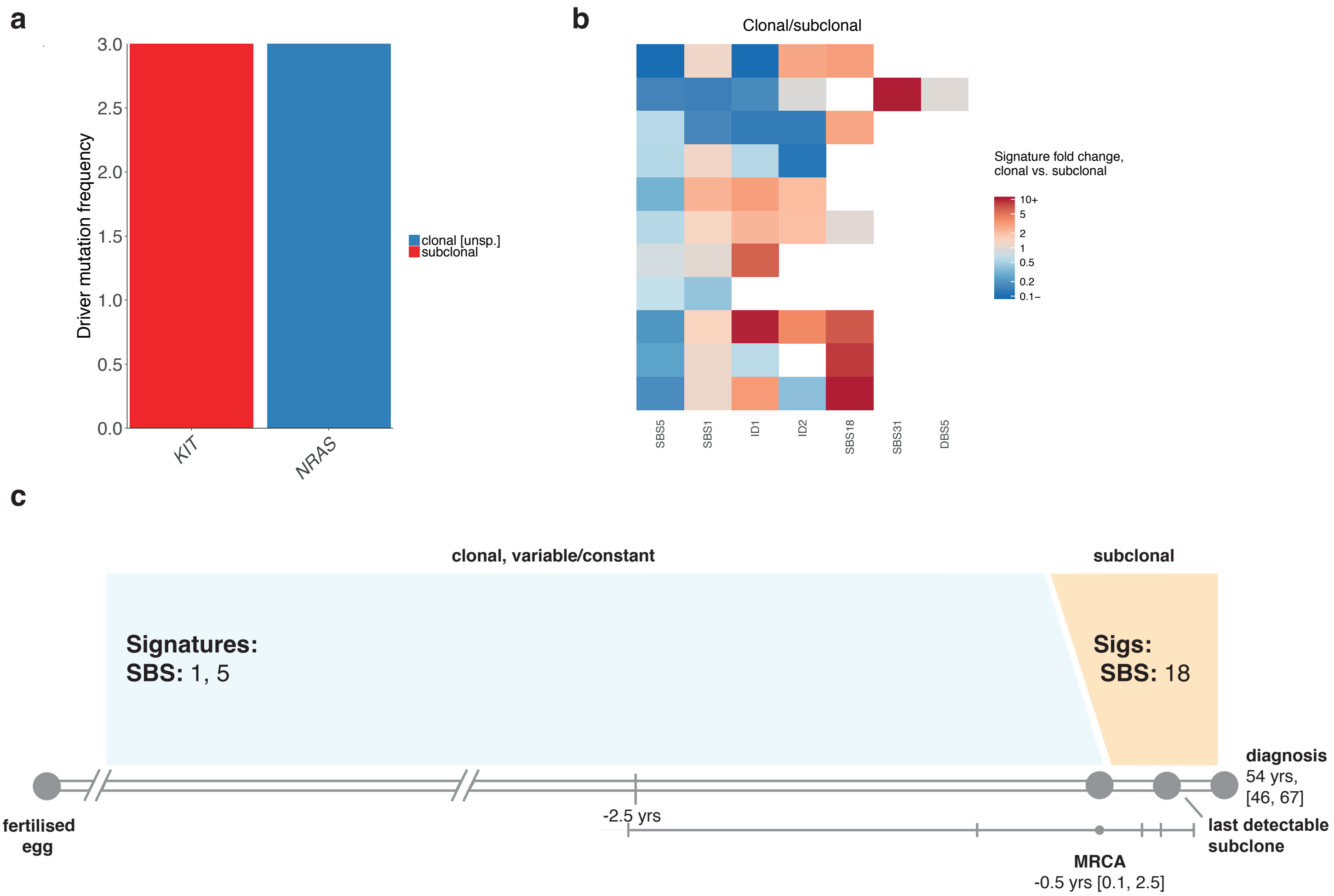

Supplementary Figure 5. Summary of all results obtained for acute myeloid leukaemia (*n*=16). a, Distribution of mutations across early clonal, late clonal and subclonal stages, for the most common driver genes. A maximum of 10 driver genes are shown. **b**, Clustered mutational signature fold changes between clonal and subclonal stages, per patient. Blue indicates a signature decrease and red an increase in subclonal from clonal mutations. **c**, Typical timeline of tumour development.

B-cell non-Hodgkin lymphoma

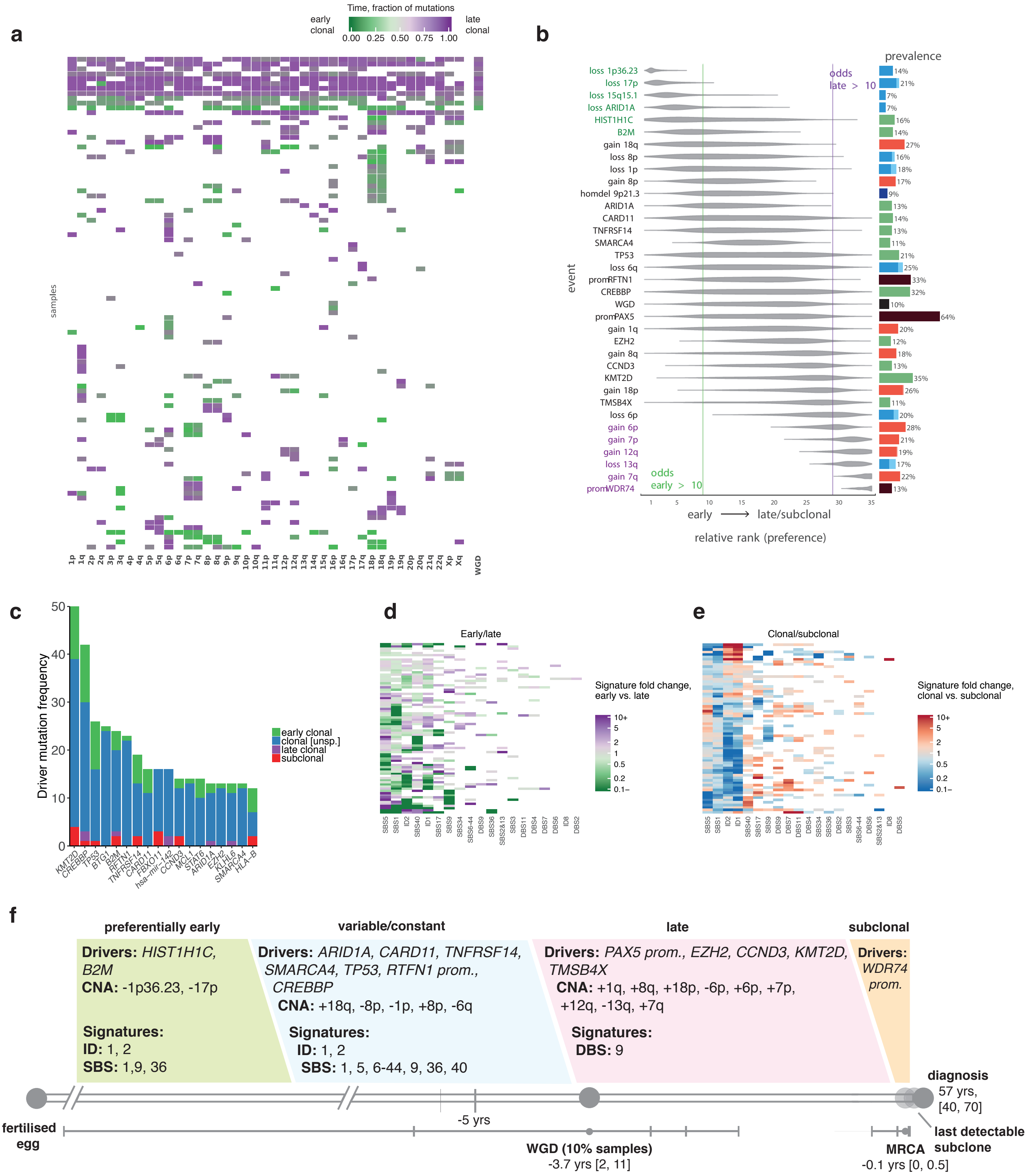

Supplementary Figure 6. Summary of all results obtained for B-cell non-Hodgkin lymphoma (*n*=107). a, Clustered heatmaps of mutational timing estimates for gained segments, per patient. Colours as indicated in main text: green represents early clonal events, purple represents late clonal. **b**, Relative ordering of copy number events and driver mutations across all samples. **c**, Distribution of mutations across early clonal, late clonal and subclonal stages, for the most common driver genes. A maximum of 10 driver genes are shown. **d**, Clustered mutational signature fold changes between early clonal and late clonal stages, per patient. Green and purple indicate, respectively, a signature decrease and increase in late clonal from early clonal mutations. Inactive signatures are coloured white. **e**, As in **d** but for clonal versus subclonal stages. Blue indicates a signature decrease and red an increase in subclonal from clonal mutations. **f**, Typical timeline of tumour development.

Biliary adenocarcinoma

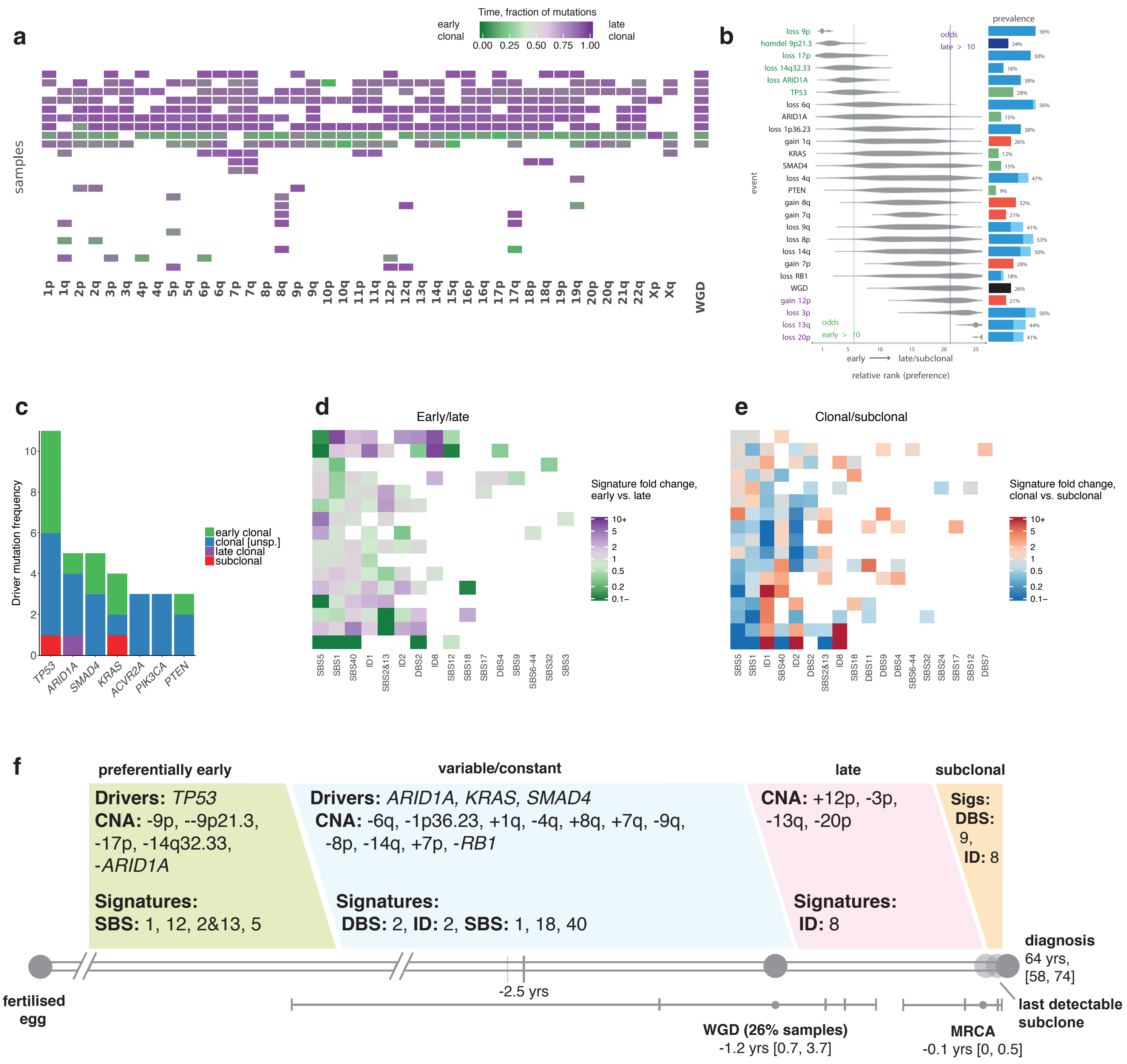

Supplementary Figure 7. Summary of all results for biliary adenocarcinoma (*n*=34). a, Clustered heatmaps of mutational timing estimates for gained segments, per patient. Colours as indicated in main text: green represents early clonal events, purple represents late clonal. **b**, Relative ordering of copy number events and driver mutations across all samples. **c**, Distribution of mutations across early clonal, late clonal and subclonal stages, for the most common driver genes. A maximum of 10 driver genes are shown. **d**, Clustered mutational signature fold changes between early clonal and late clonal stages, per patient. Green and purple indicate, respectively, a signature decrease and increase in late clonal from early clonal mutations. Inactive signatures are coloured white. **e**, As in **d** but for clonal versus subclonal stages. Blue indicates a signature decrease and red an increase in subclonal from clonal mutations. **f**, Typical timeline of tumour development.

Breast adenocarcinoma

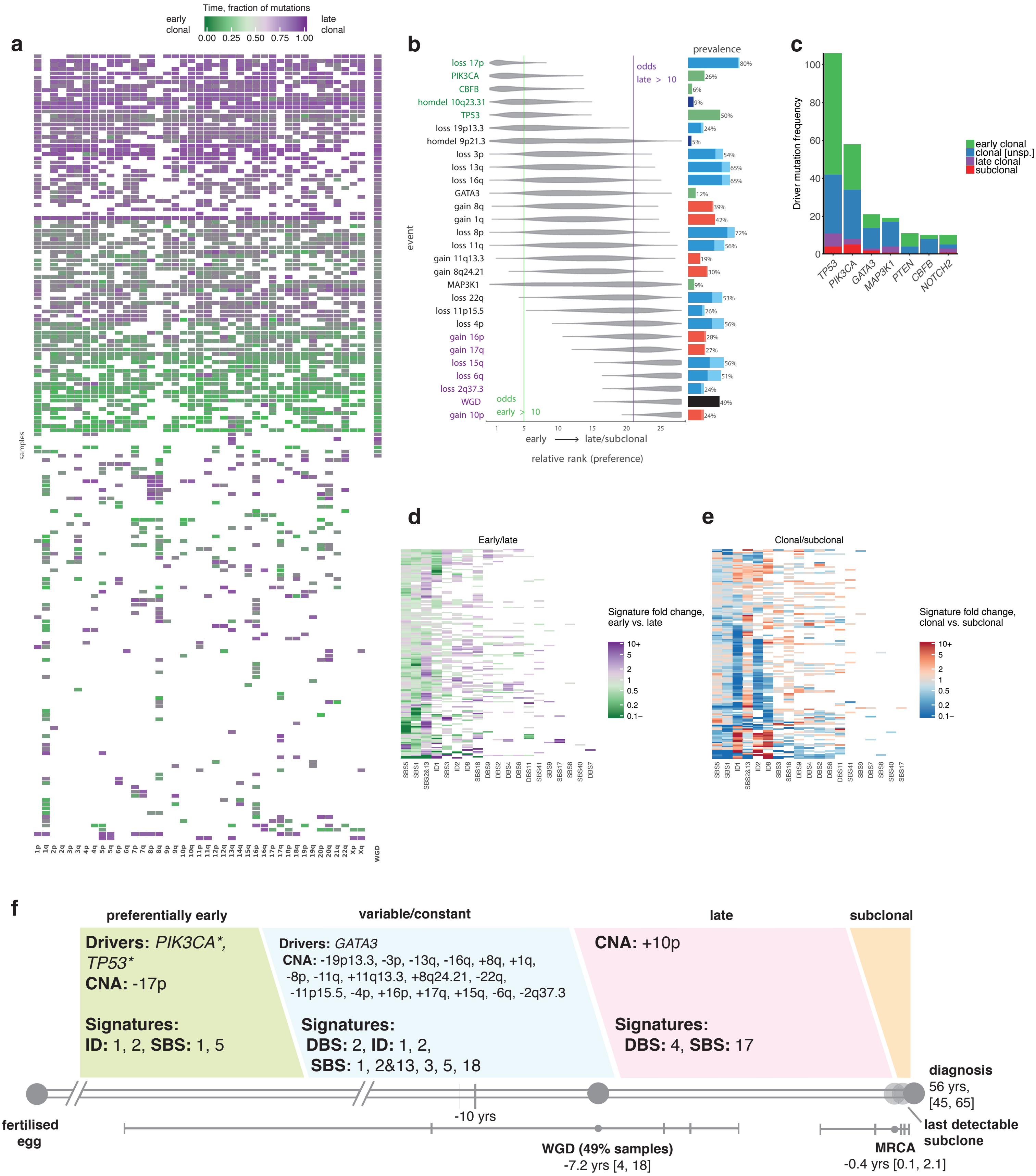

Supplementary Figure 8. Summary of all results obtained for breast adenocarcinoma (*n*=198). **a**, Clustered heatmaps of mutational timing estimates for gained segments, per patient. Colours as indicated in main text: green represents early clonal events, purple represents late clonal. **b**, Relative ordering of copy number events and driver mutations across all samples. **c**, Distribution of mutations across early clonal, late clonal and subclonal stages, for the most common driver genes. A maximum of 10 driver genes are shown. **d**, Clustered mutational signature fold changes between early clonal and late clonal stages, per patient. Green and purple indicate, respectively, a signature decrease and increase in late clonal from early clonal mutations. Inactive signatures are coloured white. **e**, As in **d** but for clonal versus subclonal stages. Blue indicates a signature decrease and red an increase in subclonal from clonal mutations. **f**, Typical timeline of tumour development.

Chromophobe renal cell carcinoma

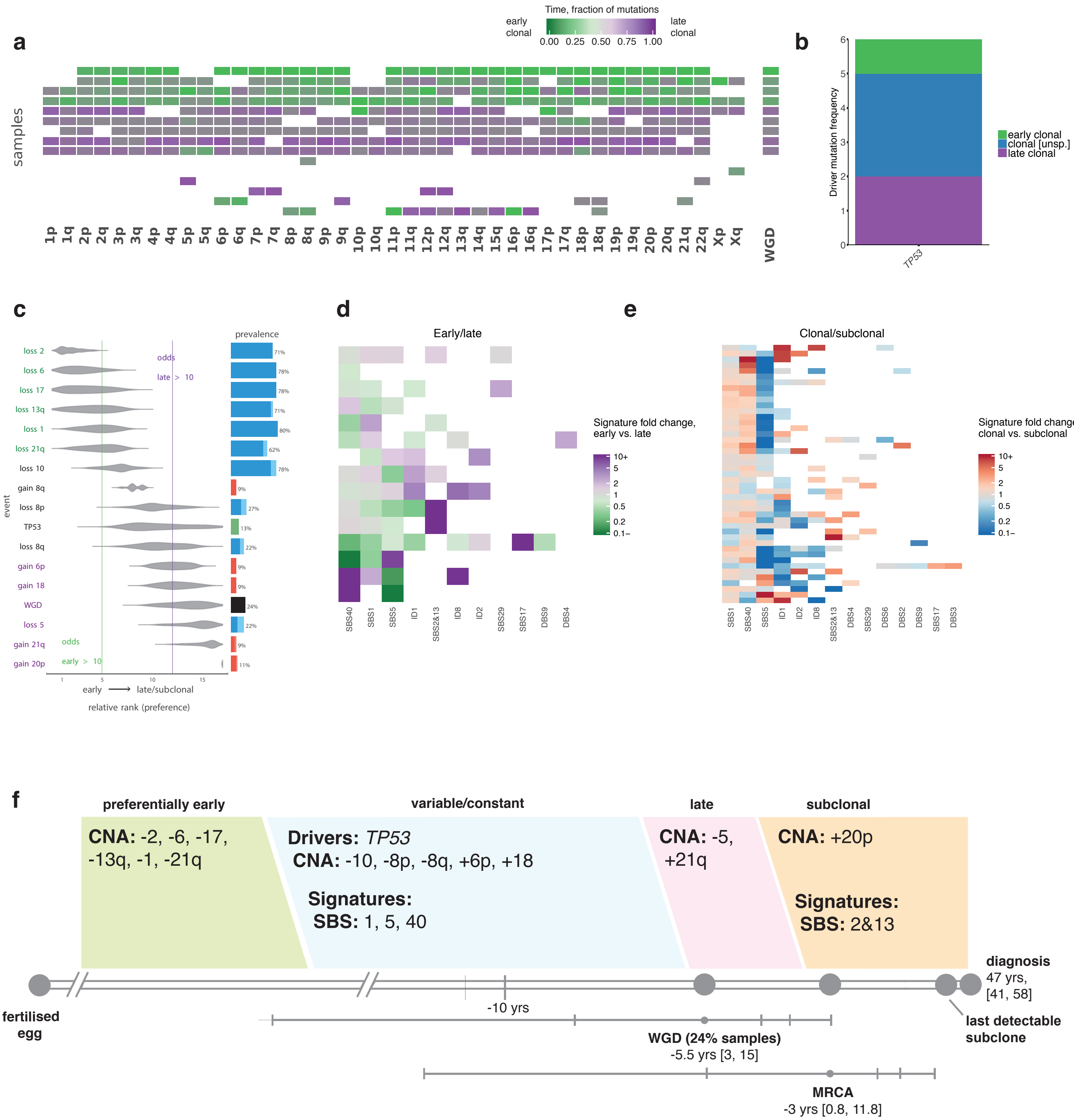

Supplementary Figure 9. Summary of all results obtained for chromophobe renal cell carcinoma (*n*=45). a, Clustered heatmaps of mutational timing estimates for gained segments, per patient. Colours as indicated in main text: green represents early clonal events, purple represents late clonal. **b**, Relative ordering of copy number events and driver mutations across all samples. **c**, Distribution of mutations across early clonal, late clonal and subclonal stages, for the most common driver genes. A maximum of 10 driver genes are shown. **d**, Clustered mutational signature fold changes between early clonal and late clonal stages, per patient. Green and purple indicate, respectively, a signature decrease and increase in late clonal from early clonal mutations. Inactive signatures are coloured white. **e**, As in **d** but for clonal versus subclonal stages. Blue indicates a signature decrease and red an increase in subclonal from clonal mutations. **f**, Typical timeline of tumour development.

Chronic lymphocytic leukaemia

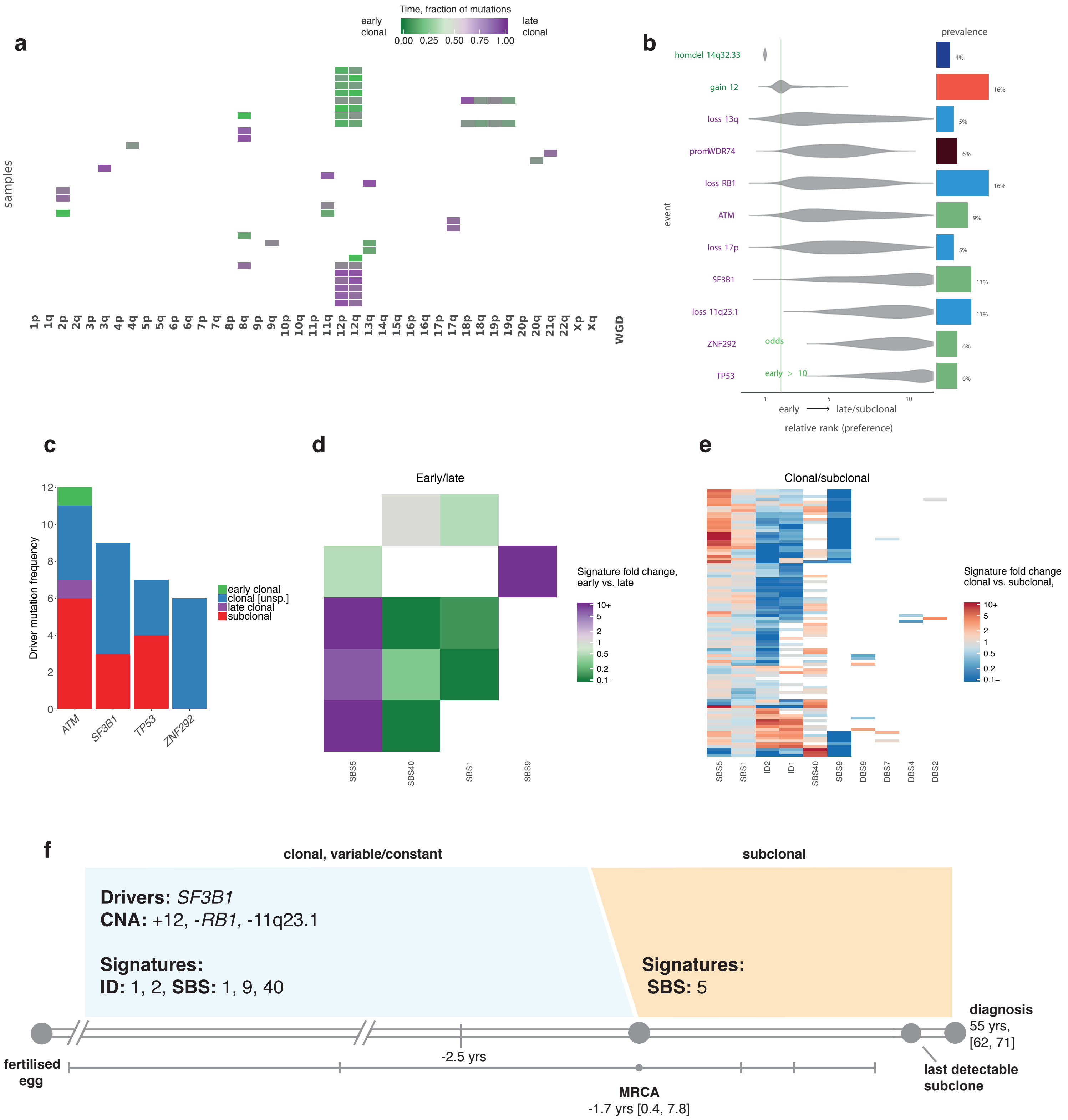

Supplementary Figure 10. Summary of all results obtained for chronic lymphocytic leukaemia (*n*=95). a, Clustered heatmaps of mutational timing estimates for gained segments, per patient. Colours as indicated in main text: green represents early clonal events, purple represents late clonal. **b**, Relative ordering of copy number events and driver mutations across all samples. **c**, Distribution of mutations across early clonal, late clonal and subclonal stages, for the most common driver genes. A maximum of 10 driver genes are shown. **d**, Clustered mutational signature fold changes between early clonal and late clonal stages, per patient. Green and purple indicate, respectively, a signature decrease and increase in late clonal from early clonal mutations. Inactive signatures are coloured white. **e**, As in **d** but for clonal versus subclonal stages. Blue indicates a signature decrease and red an increase in subclonal from clonal mutations. **f**, Typical timeline of tumour development.

Clear cell renal cell carcinoma

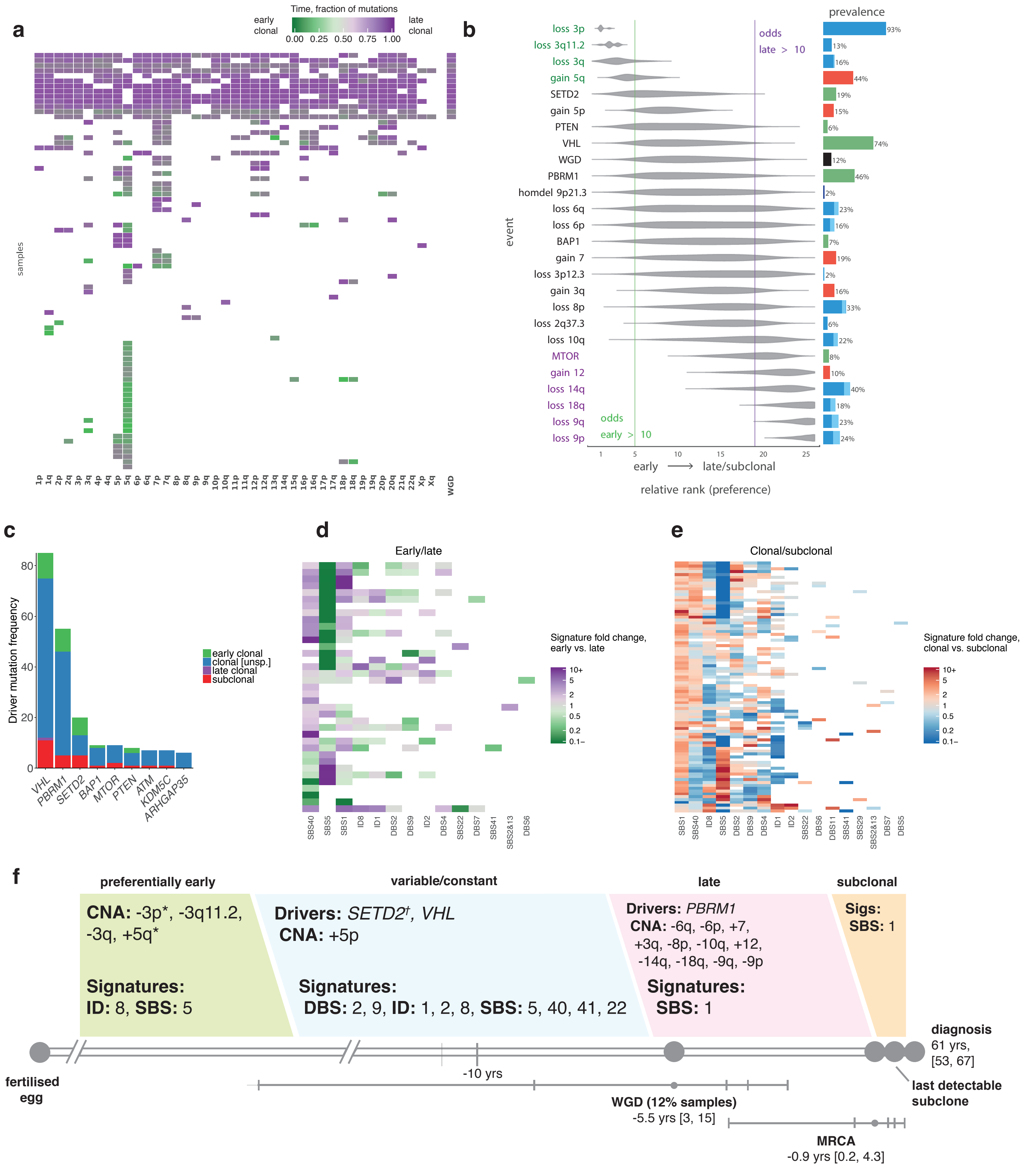

Supplementary Figure 11. Summary of all results obtained for clear cell renal cell carcinoma (*n*=111). a, Clustered heatmaps of mutational timing estimates for gained segments, per patient. Colours as indicated in main text: green represents early clonal events, purple represents late clonal. **b**, Relative ordering of copy number events and driver mutations across all samples. **c**, Distribution of mutations across early clonal, late clonal and subclonal stages, for the most common driver genes. A maximum of 10 driver genes are shown. **d**, Clustered mutational signature fold changes between early clonal and late clonal stages, per patient. Green and purple indicate, respectively, a signature decrease and increase in late clonal from early clonal mutations. Inactive signatures are coloured white. **e**, As in **d** but for clonal versus subclonal stages. Blue indicates a signature decrease and red an increase in subclonal from clonal mutations. **f**, Typical timeline of tumour development.

Colorectal adenocarcinoma

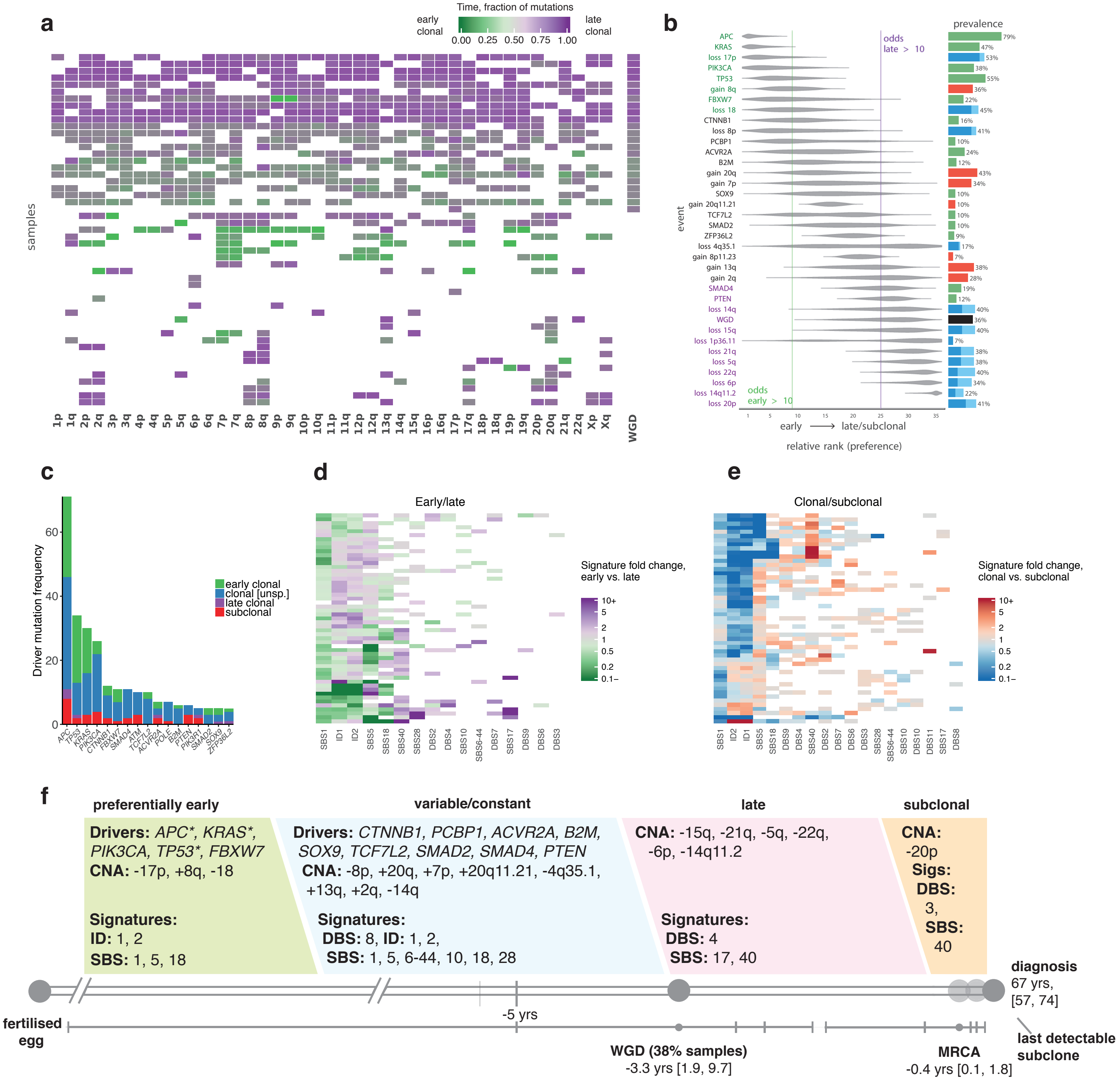

Supplementary Figure 12. Summary of all results obtained for colorectal adenocarcinoma (*n*=60). a, Clustered heatmaps of mutational timing estimates for gained segments, per patient. Colours as indicated in main text: green represents early clonal events, purple represents late clonal. **b**, Relative ordering of copy number events and driver mutations across all samples. **c**, Distribution of mutations across early clonal, late clonal and subclonal stages, for the most common driver genes. A maximum of 10 driver genes are shown. **d**, Clustered mutational signature fold changes between early clonal and late clonal stages, per patient. Green and purple indicate, respectively, a signature decrease and increase in late clonal from early clonal mutations. Inactive signatures are coloured white. **e**, As in **d** but for clonal versus subclonal stages. Blue indicates a signature decrease and red an increase in subclonal from clonal mutations. **f**, Typical timeline of tumour development.

Endometrial adenocarcinoma

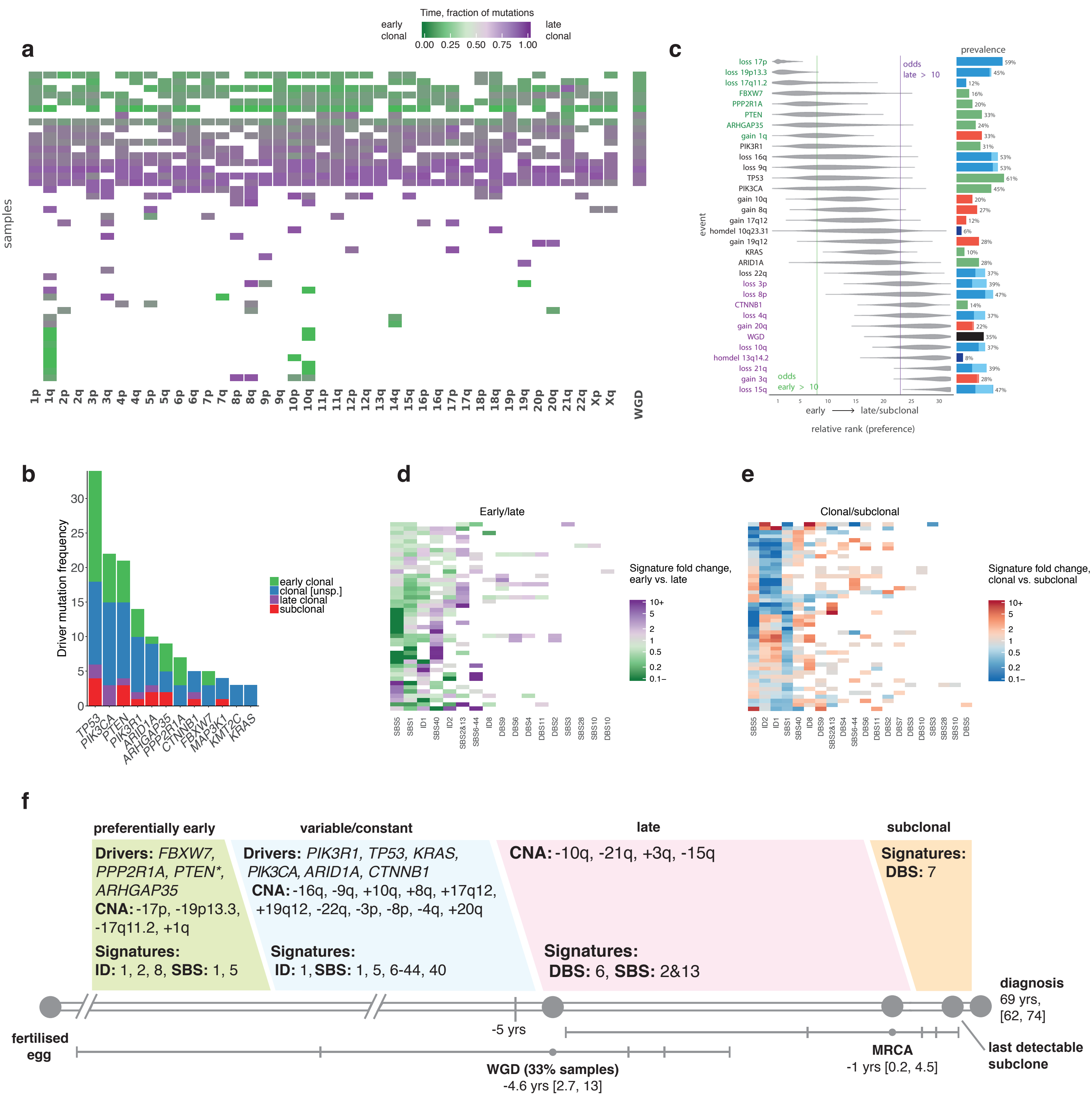

Supplementary Figure 13. Summary of all results obtained for endometrial adenocarcinoma (*n*=51). a, Clustered heatmaps of mutational timing estimates for gained segments, per patient. Colours as indicated in main text: green represents early clonal events, purple represents late clonal. **b**, Relative ordering of copy number events and driver mutations across all samples. **c**, Distribution of mutations across early clonal, late clonal and subclonal stages, for the most common driver genes. A maximum of 10 driver genes are shown. **d**, Clustered mutational signature fold changes between early clonal and late clonal stages, per patient. Green and purple indicate, respectively, a signature decrease and increase in late clonal from early clonal mutations. Inactive signatures are coloured white. **e**, As in **d** but for clonal versus subclonal stages. Blue indicates a signature decrease and red an increase in subclonal from clonal mutations. **f**, Typical timeline of tumour development.

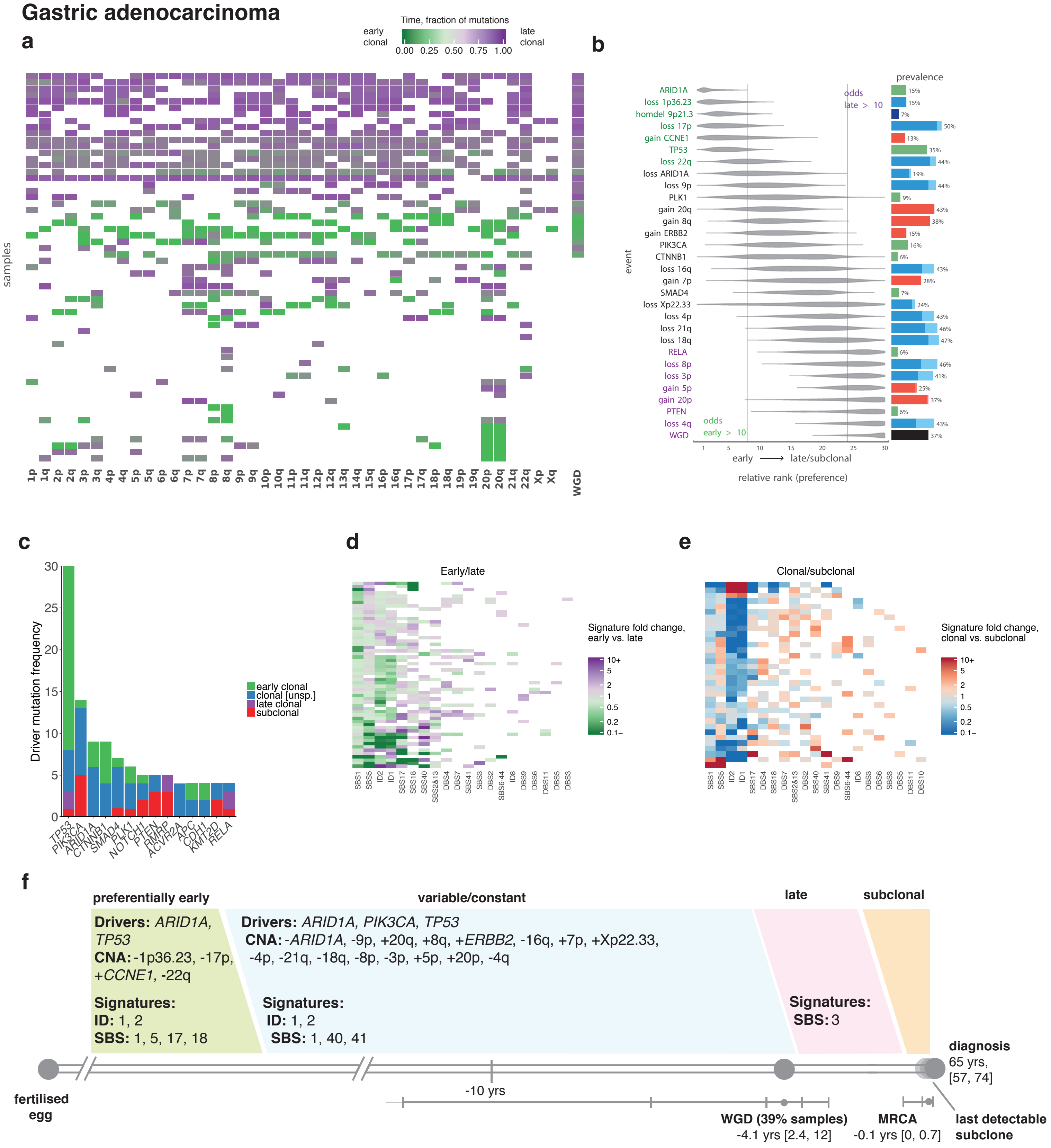

Supplementary Figure 14. Summary of all results obtained for gastric adenocarcinoma (*n*=75). **a**, Clustered heatmaps of mutational timing estimates for gained segments, per patient. Colours as indicated in main text: green represents early clonal events, purple represents late clonal. **b**, Relative ordering of copy number events and driver mutations across all samples. **c**, Distribution of mutations across early clonal, late clonal and subclonal stages, for the most common driver genes. A maximum of 10 driver genes are shown. **d**, Clustered mutational signature fold changes between early clonal and late clonal stages, per patient. Green and purple indicate, respectively, a signature decrease and increase in late clonal from early clonal mutations. Inactive signatures are coloured white. **e**, As in **d** but for clonal versus subclonal stages. Blue indicates a signature decrease and red an increase in subclonal from clonal mutations. **f**, Typical timeline of tumour development.

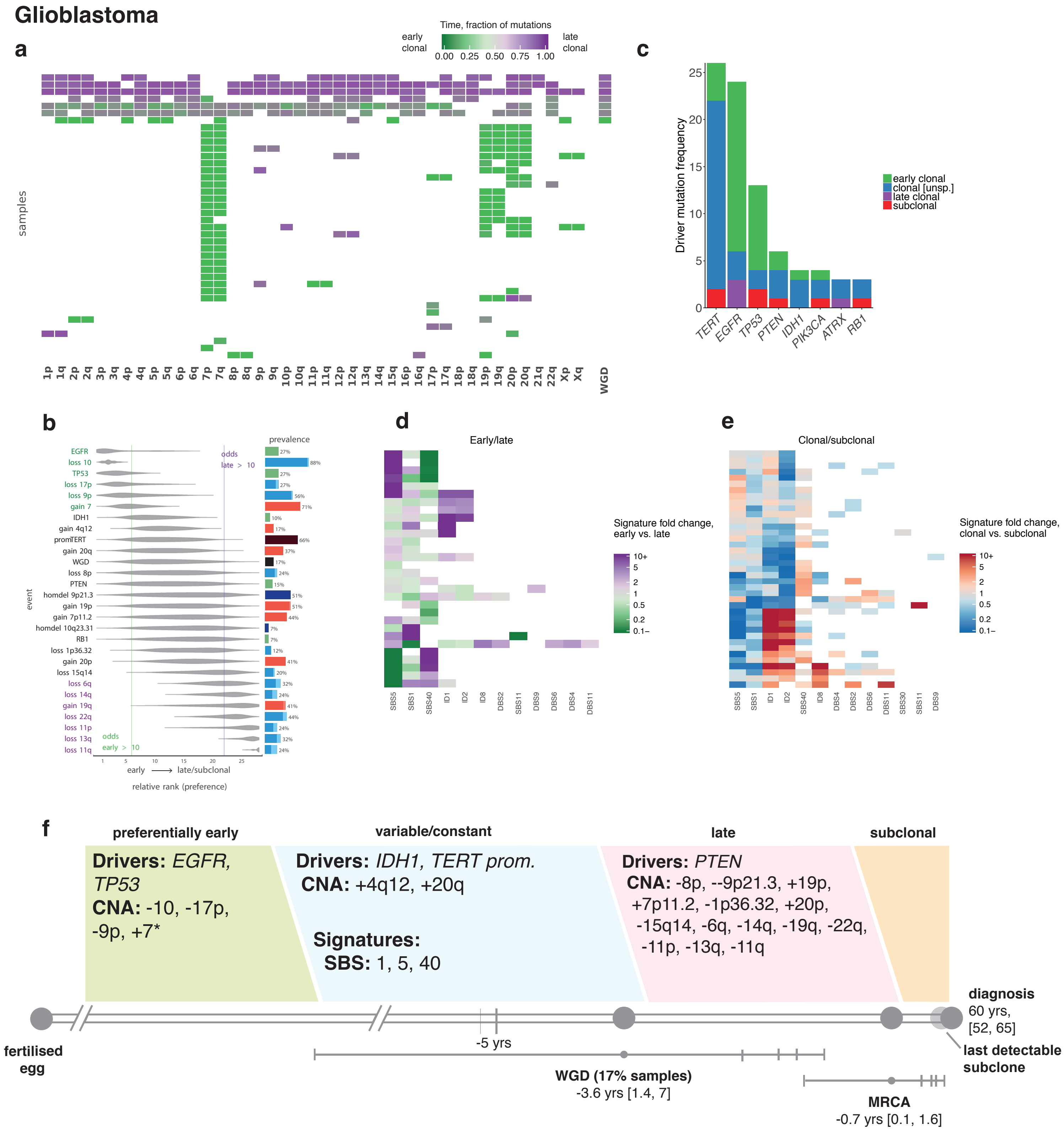

Supplementary Figure 15. Summary of all results obtained for glioblastoma (*n*=41). a, Clustered heatmaps of mutational timing estimates for gained segments, per patient. Colours as indicated in main text: green represents early clonal events, purple represents late clonal. **b**, Relative ordering of copy number events and driver mutations across all samples. **c**, Distribution of mutations across early clonal, late clonal and subclonal stages, for the most common driver genes. A maximum of 10 driver genes are shown. **d**, Clustered mutational signature fold changes between early clonal and late clonal stages, per patient. Green and purple indicate, respectively, a signature decrease and increase in late clonal from early clonal mutations. Inactive signatures are coloured white. **e**, As in **d** but for clonal versus subclonal stages. Blue indicates a signature decrease and red an increase in subclonal from clonal mutations. **f**, Typical timeline of tumour development.

Head and neck squamous cell carcinoma

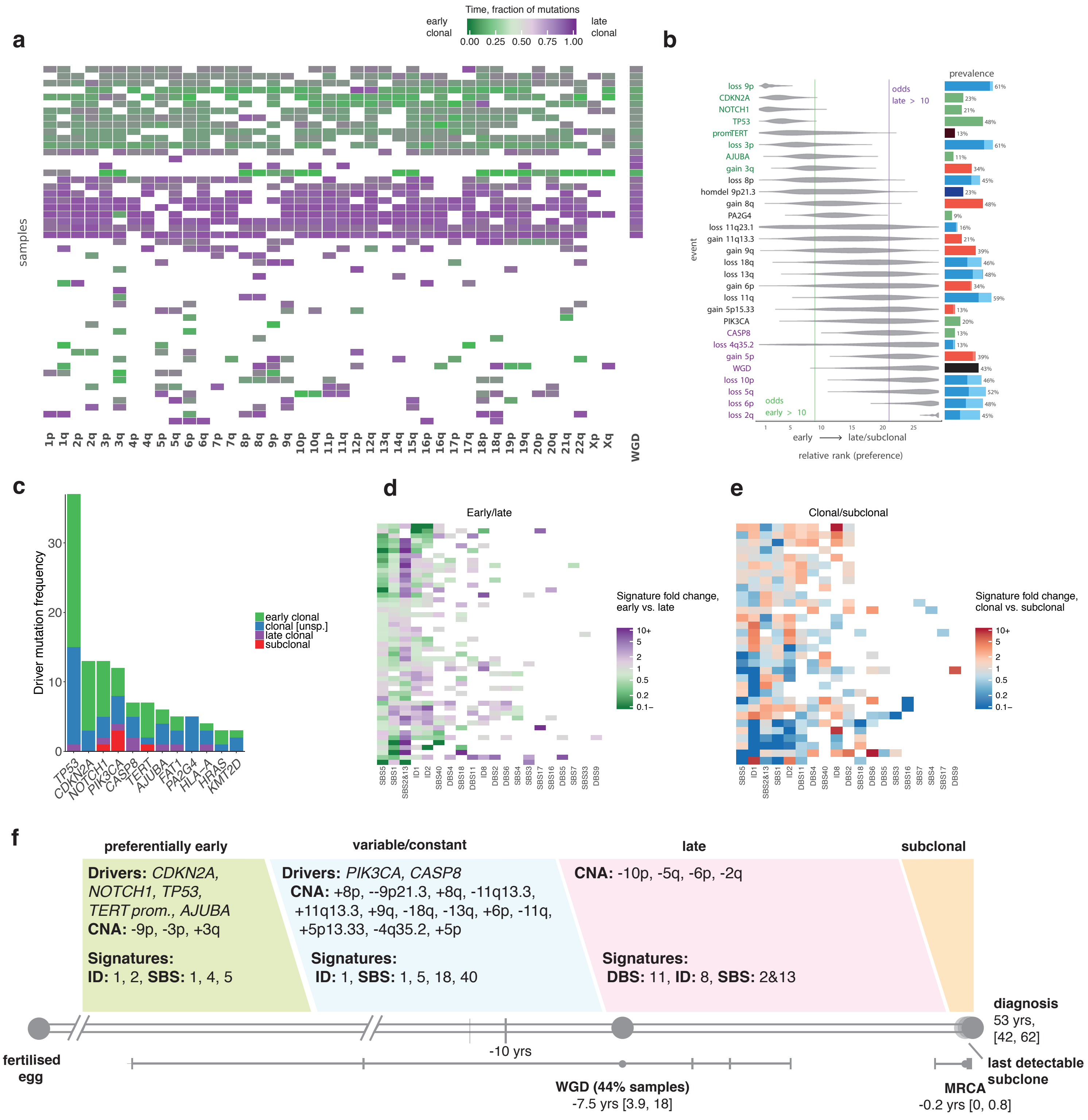

Supplementary Figure 16. Summary of all results obtained for head and neck squamous cell carcinoma (*n*=57). a, Clustered heatmaps of mutational timing estimates for gained segments, per patient. Colours as indicated in main text: green represents early clonal events, purple represents late clonal. **b**, Relative ordering of copy number events and driver mutations across all samples. **c**, Distribution of mutations across early clonal, late clonal and subclonal stages, for the most common driver genes. A maximum of 10 driver genes are shown. **d**, Clustered mutational signature fold changes between early clonal and late clonal stages, per patient. Green and purple indicate, respectively, a signature decrease and increase in late clonal from early clonal mutations. Inactive signatures are coloured white. **e**, As in **d** but for clonal versus subclonal stages. Blue indicates a signature decrease and red an increase in subclonal from clonal mutations. **f**, Typical timeline of tumour development.

Liver hepatocellular carcinoma

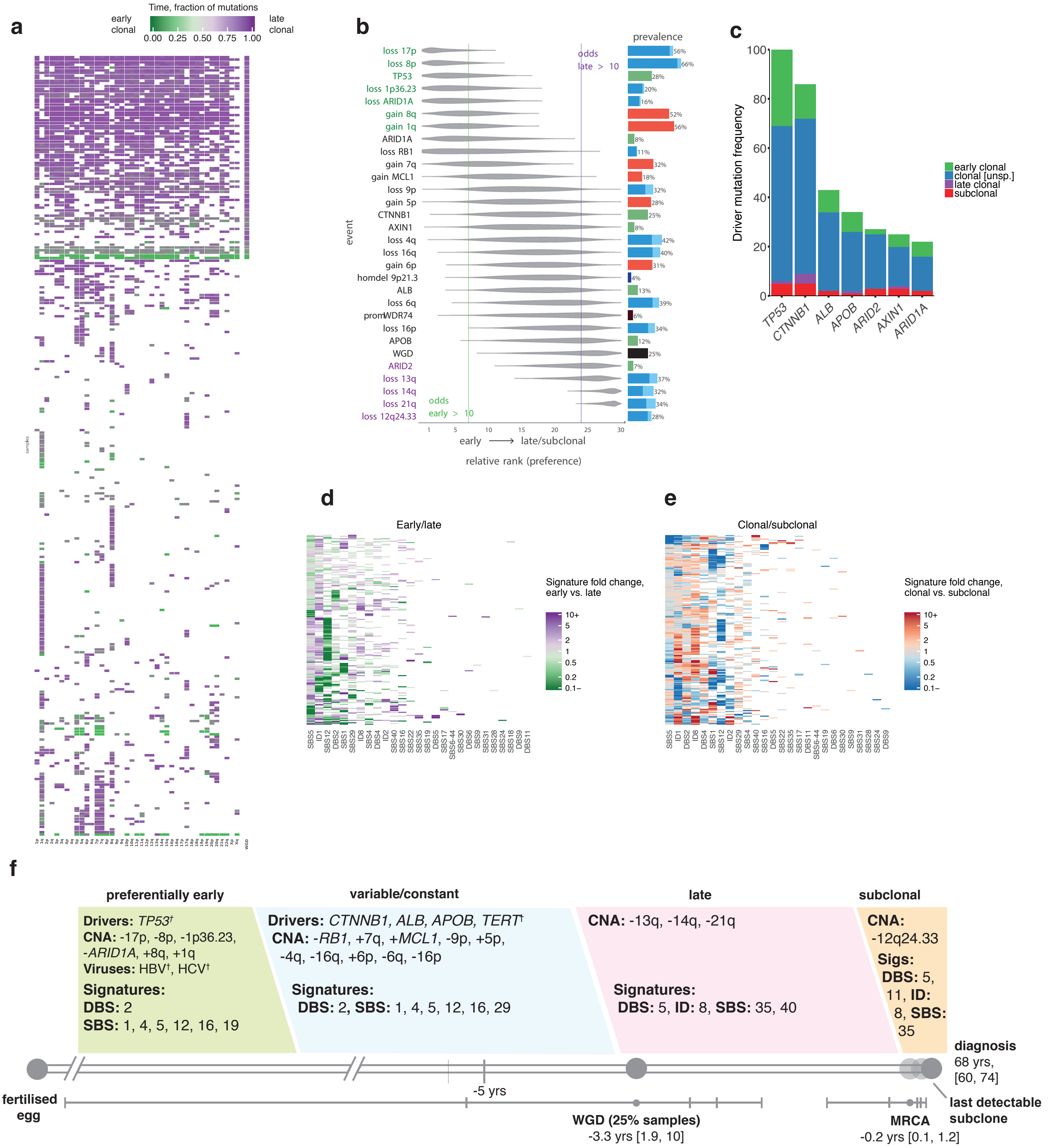

Supplementary Figure 17. Summary of all results obtained for hepatocellular carcinoma (*n*=327). a, Clustered heatmaps of mutational timing estimates for gained segments, per patient. Colours as indicated in main text: green represents early clonal events, purple represents late clonal. **b**, Relative ordering of copy number events and driver mutations across all samples. **c**, Distribution of mutations across early clonal, late clonal and subclonal stages, for the most common driver genes. A maximum of 10 driver genes are shown. **d**, Clustered mutational signature fold changes between early clonal and late clonal stages, per patient. Green and purple indicate, respectively, a signature decrease and increase in late clonal from early clonal mutations. Inactive signatures are coloured white. **e**, As in **d** but for clonal versus subclonal stages. Blue indicates a signature decrease and red an increase in subclonal from clonal mutations. **f**, Typical timeline of tumour development.

Leiomyosarcoma

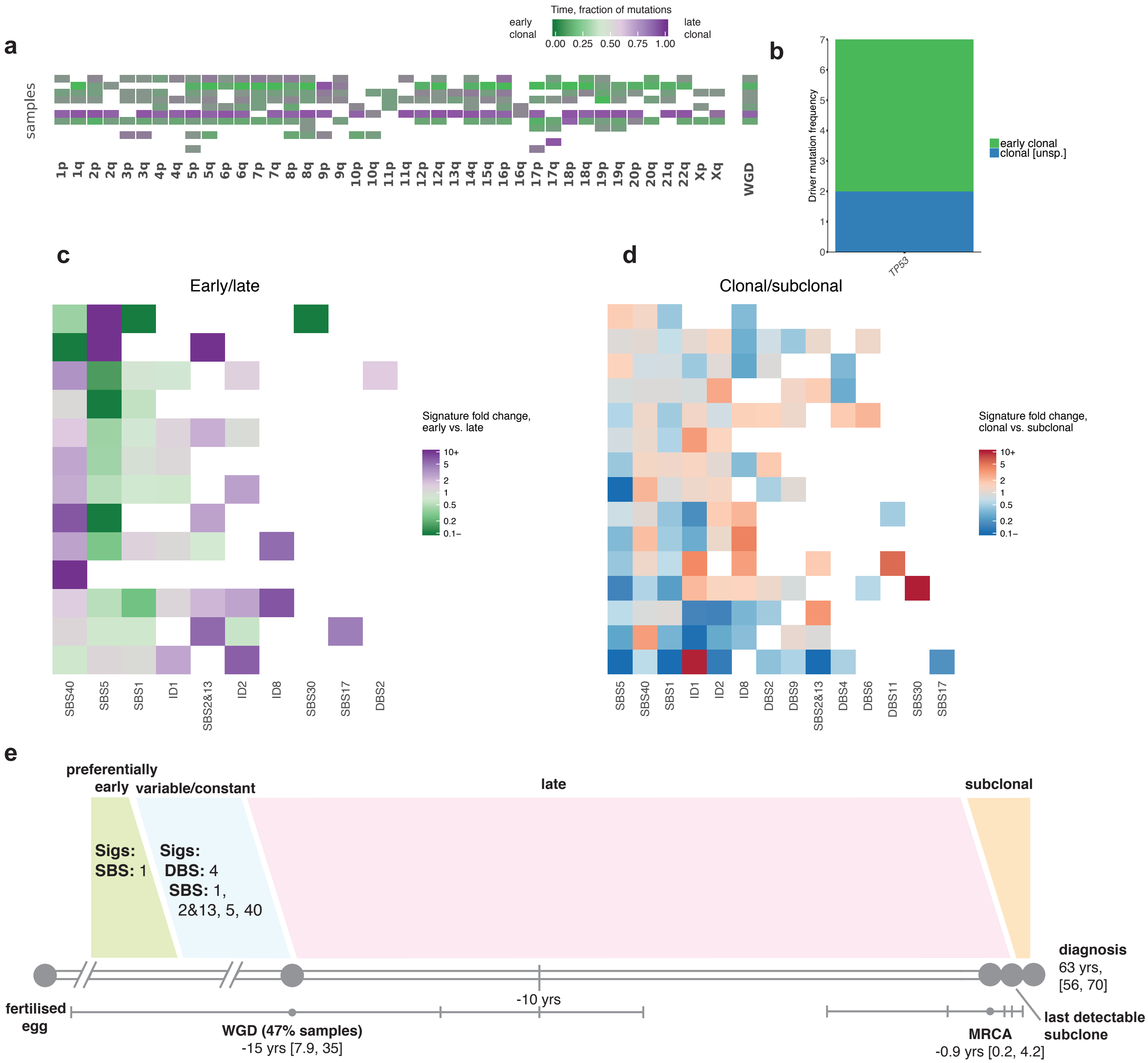

Supplementary Figure 18. Summary of all results obtained for leiomyosarcoma (*n*=15). a, Clustered heatmaps of mutational timing estimates for gained segments, per patient. Colours as indicated in main text: green represents early clonal events, purple represents late clonal. **b**, Distribution of mutations across early clonal, late clonal and subclonal stages, for the most common driver genes. A maximum of 10 driver genes are shown. **c**, Clustered mutational signature fold changes between early clonal and late clonal stages, per patient. Green and purple indicate, respectively, a signature decrease and increase in late clonal from early clonal mutations. Inactive signatures are coloured white. **d**, As in **c** but for clonal versus subclonal stages. Blue indicates a signature decrease and red an increase in subclonal from clonal mutations. **e**, Typical timeline of tumour development.

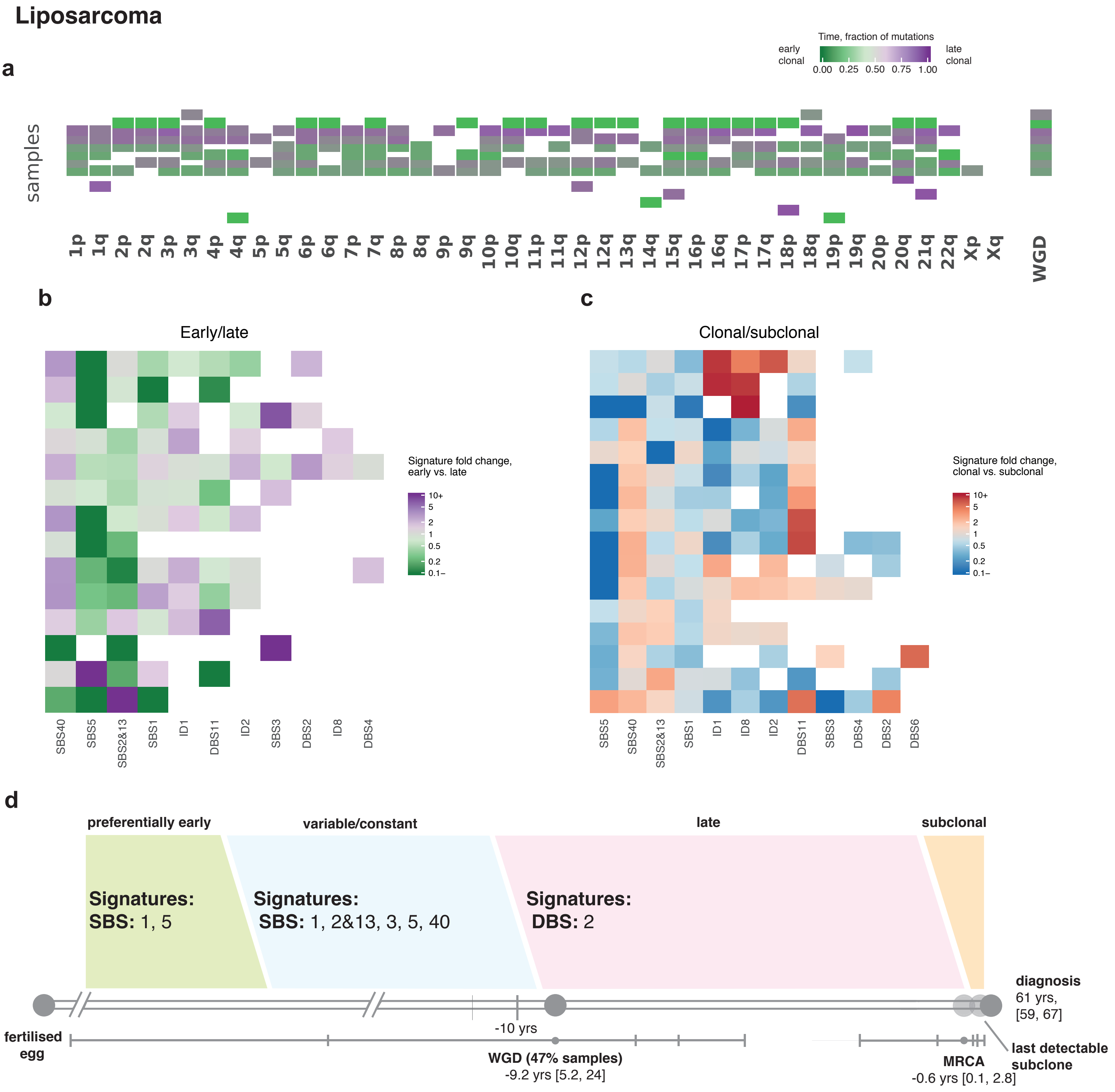

Supplementary Figure 19. Summary of all results obtained for liposarcoma (*n*=19). a, Clustered heatmaps of mutational timing estimates for gained segments, per patient. Colours as indicated in main text: green represents early clonal events, purple represents late clonal. **b**, Clustered mutational signature fold changes between early clonal and late clonal stages, per patient. Green and purple indicate, respectively, a signature decrease and increase in late clonal from early clonal mutations. Inactive signatures are coloured white. **c**, As in **b** but for clonal versus subclonal stages. Blue indicates a signature decrease and red an increase in subclonal from clonal mutations. **d**, Typical timeline of tumour development.

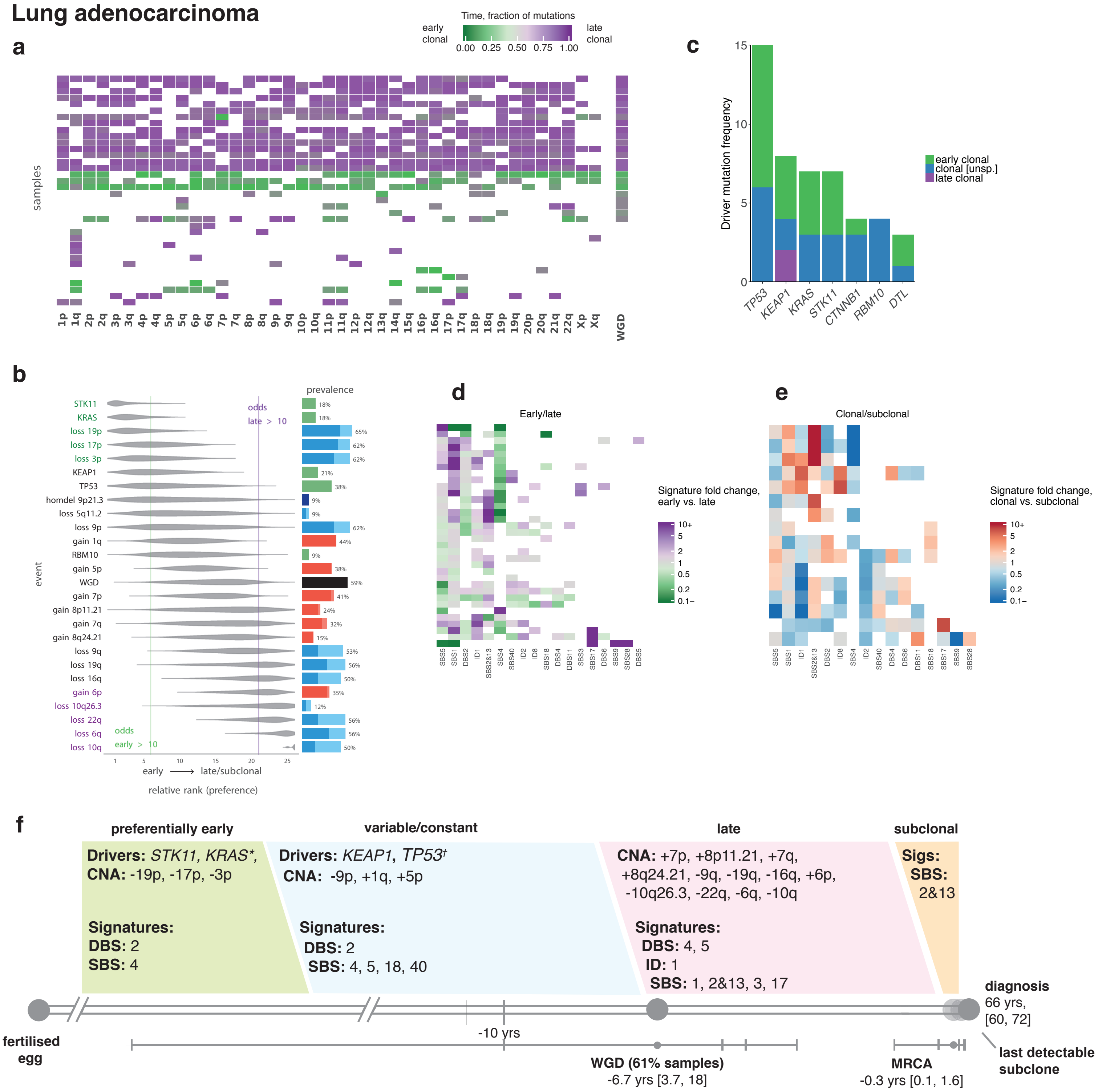

Supplementary Figure 20. Summary of all results obtained for lung adenocarcinoma (*n*=38). a, Clustered heatmaps of mutational timing estimates for gained segments, per patient. Colours as indicated in main text: green represents early clonal events, purple represents late clonal. **b**, Relative ordering of copy number events and driver mutations across all samples. **c**, Distribution of mutations across early clonal, late clonal and subclonal stages, for the most common driver genes. A maximum of 10 driver genes are shown. **d**, Clustered mutational signature fold changes between early clonal and late clonal stages, per patient. Green and purple indicate, respectively, a signature decrease and increase in late clonal from early clonal mutations. Inactive signatures are coloured white. **e**, As in **d** but for clonal versus subclonal stages. Blue indicates a signature decrease and red an increase in subclonal from clonal mutations. **f**, Typical timeline of tumour development.

Medulloblastoma

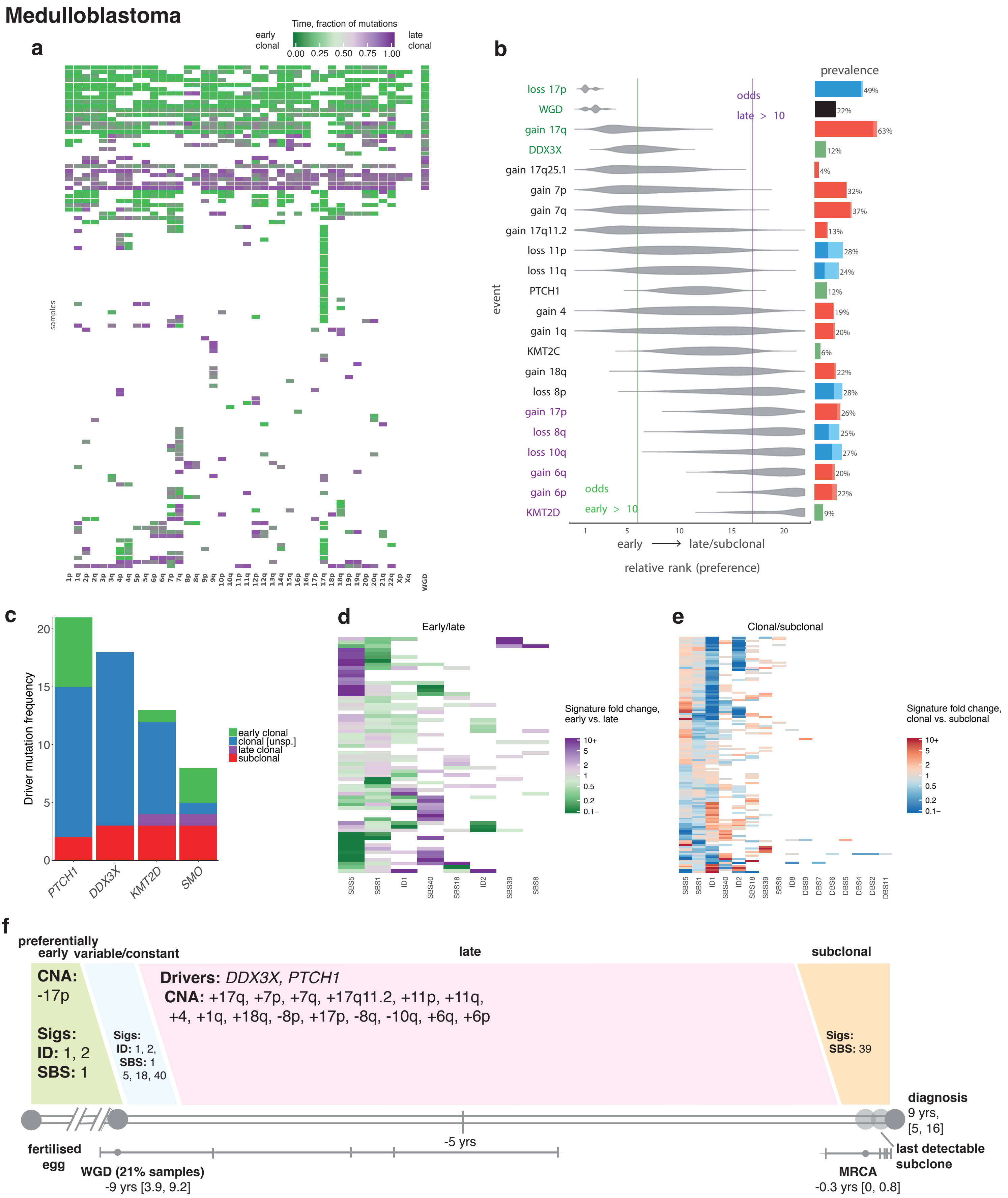

Supplementary Figure 21. Summary of all results obtained for medulloblastoma (*n*=146). a, Clustered heatmaps of mutational timing estimates for gained segments, per patient. Colours as indicated in main text: green represents early clonal events, purple represents late clonal. **b**, Relative ordering of copy number events and driver mutations across all samples. **c**, Distribution of mutations across early clonal, late clonal and subclonal stages, for the most common driver genes. A maximum of 10 driver genes are shown. **d**, Clustered mutational signature fold changes between early clonal and late clonal stages, per patient. Green and purple indicate, respectively, a signature decrease and increase in late clonal from early clonal mutations. Inactive signatures are coloured white. **e**, As in **d** but for clonal versus subclonal stages. Blue indicates a signature decrease and red an increase in subclonal from clonal mutations. **f**, Typical timeline of tumour development.

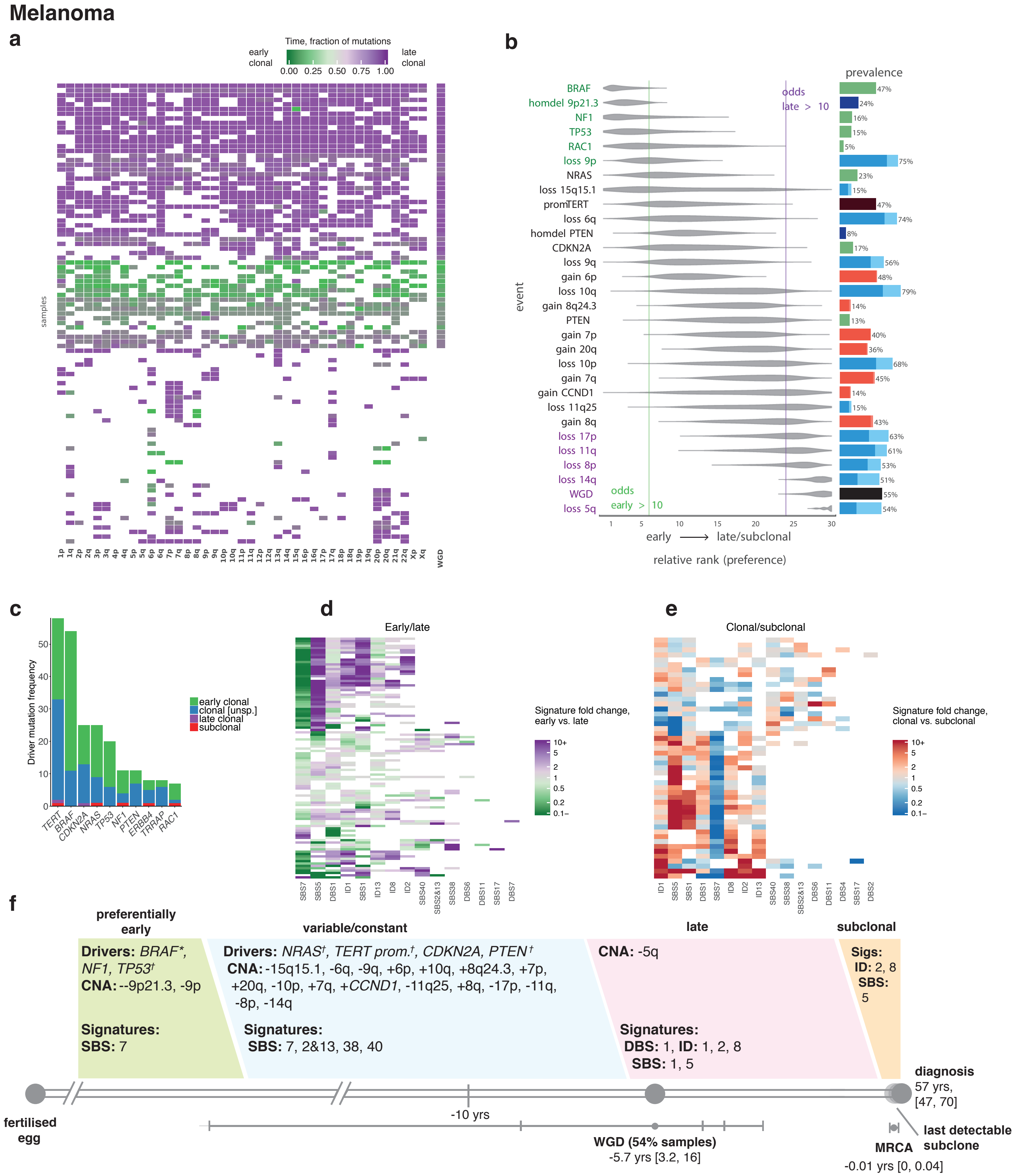

Supplementary Figure 22. Summary of all results obtained for melanoma (*n*=107). a, Clustered heatmaps of mutational timing estimates for gained segments, per patient. Colours as indicated in main text: green represents early clonal events, purple represents late clonal. **b**, Relative ordering of copy number events and driver mutations across all samples. **c**, Distribution of mutations across early clonal, late clonal and subclonal stages, for the most common driver genes. A maximum of 10 driver genes are shown. **d**, Clustered mutational signature fold changes between early clonal and late clonal stages, per patient. Green and purple indicate, respectively, a signature decrease and increase in late clonal from early clonal mutations. Inactive signatures are coloured white. **e**, As in **d** but for clonal versus subclonal stages. Blue indicates a signature decrease and red an increase in subclonal from clonal mutations. **f**, Typical timeline of tumour development.

Myeloproliferative neoplasms

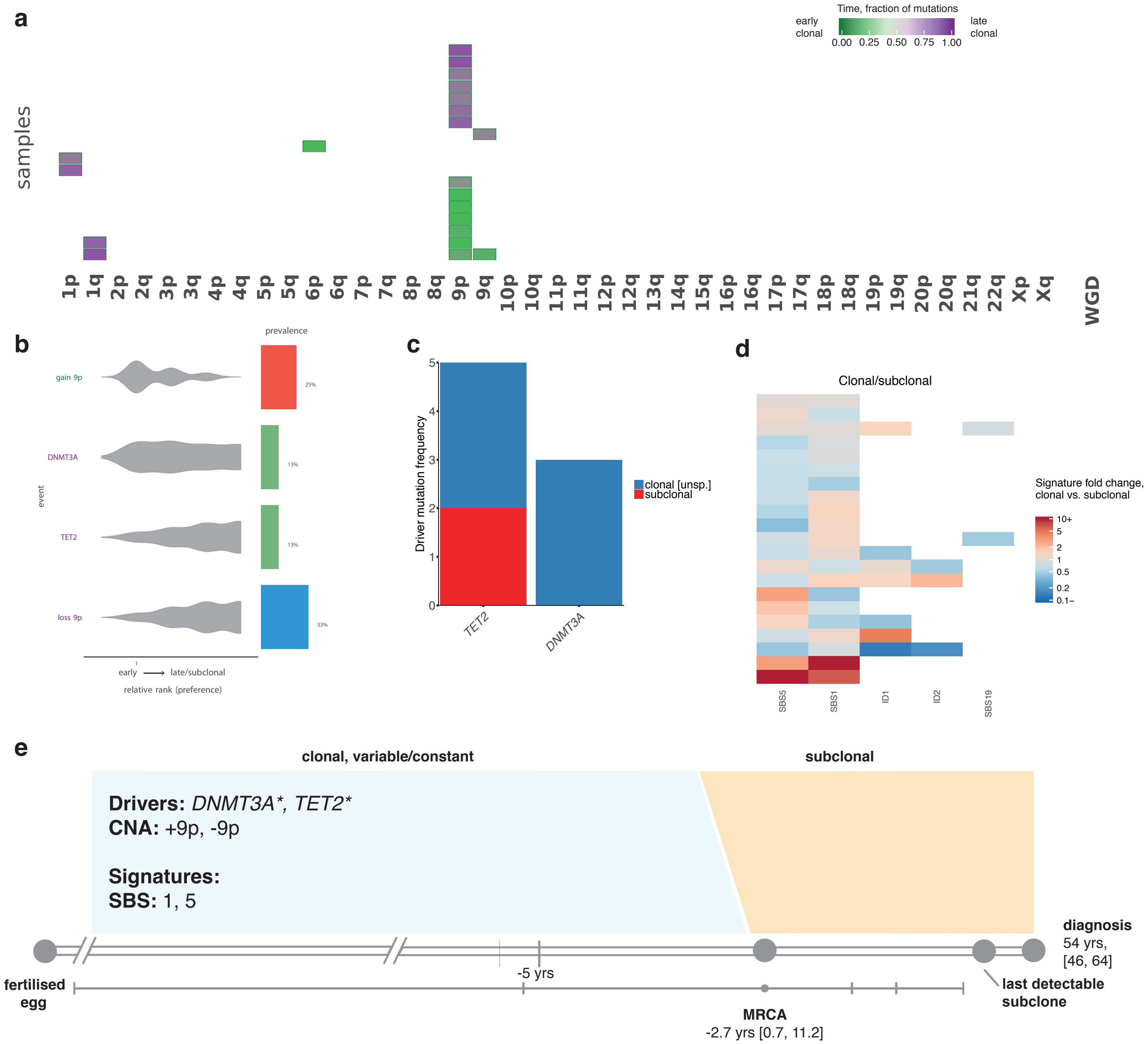

Supplementary Figure 23. Summary of all results obtained for myeloproliferative neoplasms (*n*=51). a, Clustered heatmaps of mutational timing estimates for gained segments, per patient. Colours as indicated in main text: green represents early clonal events, purple represents late clonal. **b**, Relative ordering of copy number events and driver mutations across all samples. **c**, Distribution of mutations across early clonal, late clonal and subclonal stages, for the most common driver genes. A maximum of 10 driver genes are shown. **d**, Clustered mutational signature fold changes between clonal and subclonal stages, per patient. Blue indicates a signature decrease and red an increase in subclonal from clonal mutations. **e**, Typical timeline of tumour development.

Oesophageal adenocarcinoma

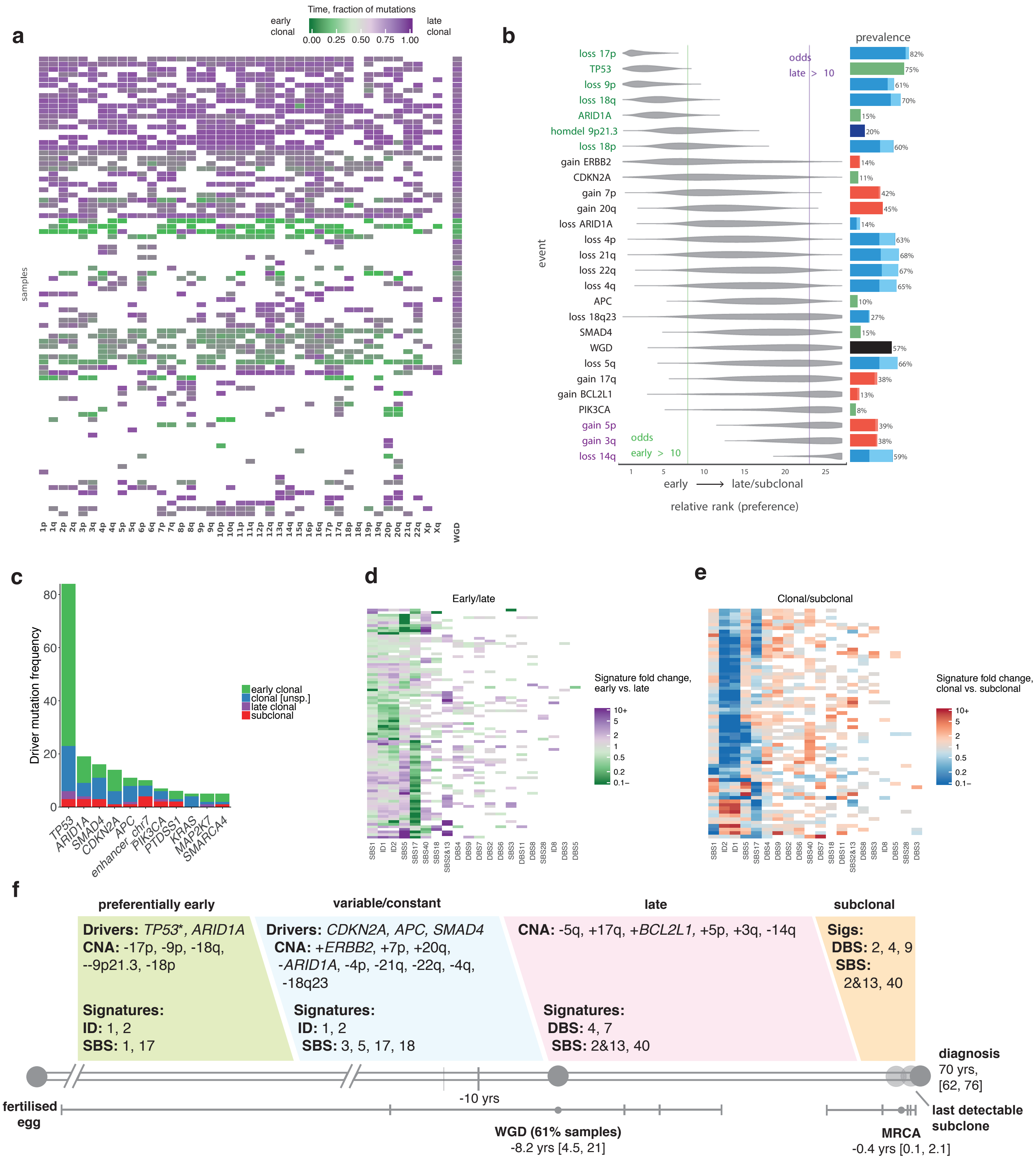

Supplementary Figure 24. Summary of all results obtained for oesophageal adenocarcinoma (*n*=98). a, Clustered heatmaps of mutational timing estimates for gained segments, per patient. Colours as indicated in main text: green represents early clonal events, purple represents late clonal. **b**, Relative ordering of copy number events and driver mutations across all samples. **c**, Distribution of mutations across early clonal, late clonal and subclonal stages, for the most common driver genes. A maximum of 10 driver genes are shown. **d**, Clustered mutational signature fold changes between early clonal and late clonal stages, per patient. Green and purple indicate, respectively, a signature decrease and increase in late clonal from early clonal mutations. Inactive signatures are coloured white. **e**, As in **d** but for clonal versus subclonal stages. Blue indicates a signature decrease and red an increase in subclonal from clonal mutations. **f**, Typical timeline of tumour development.

Oligodendroglioma

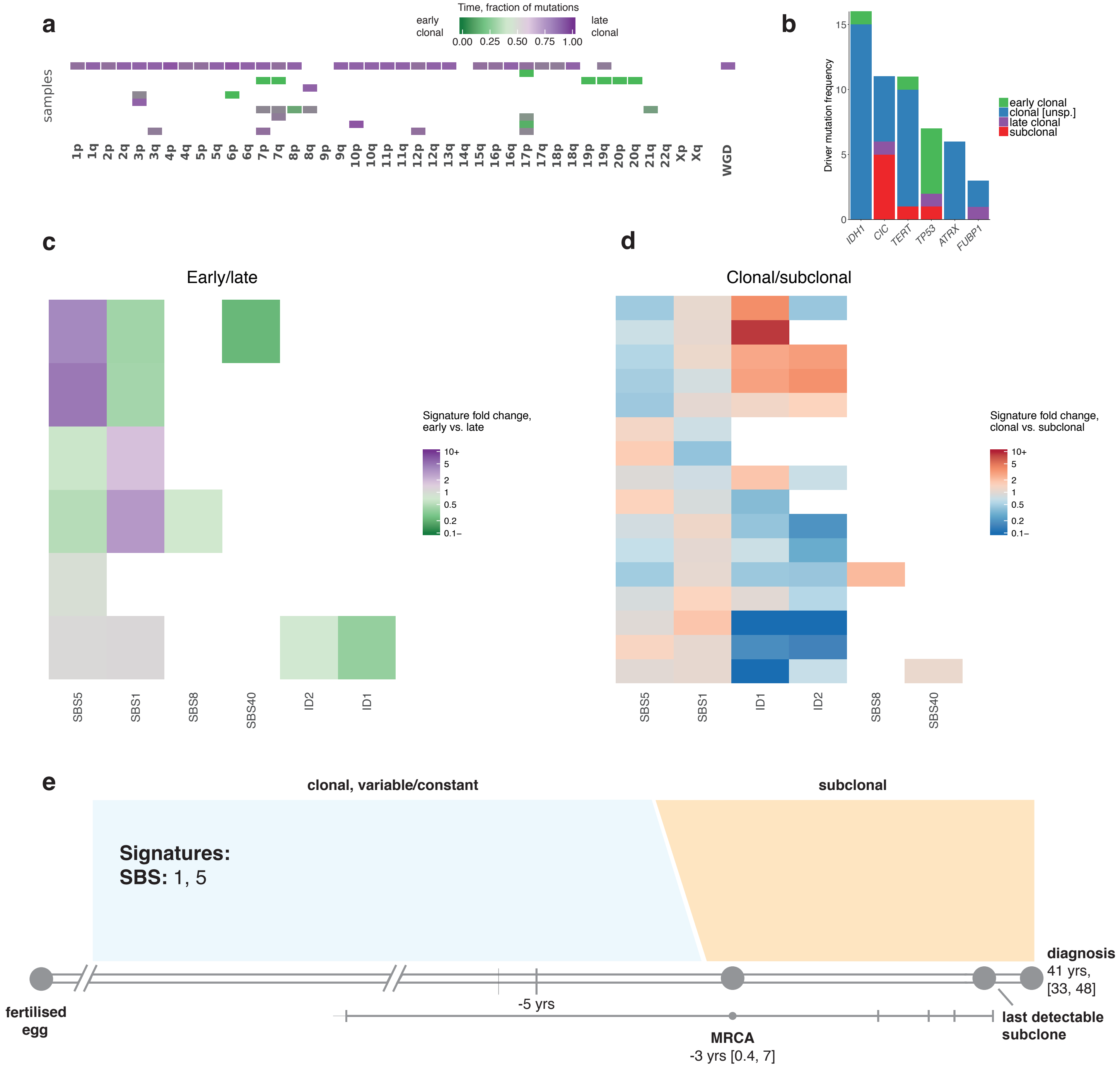

Supplementary Figure 25. Summary of all results obtained for oligodendroglioma (*n*=18). a, Clustered heatmaps of mutational timing estimates for gained segments, per patient. Colours as indicated in main text: green represents early clonal events, purple represents late clonal. **b**, Distribution of mutations across early clonal, late clonal and subclonal stages, for the most common driver genes. A maximum of 10 driver genes are shown. **c**, Clustered mutational signature fold changes between early clonal and late clonal stages, per patient. Green and purple indicate, respectively, a signature decrease and increase in late clonal from early clonal mutations. Inactive signatures are coloured white. **d**, As in **c** but for clonal versus subclonal stages. Blue indicates a signature decrease and red an increase in subclonal from clonal mutations. **e**, Typical timeline of tumour development.

Osteosarcoma

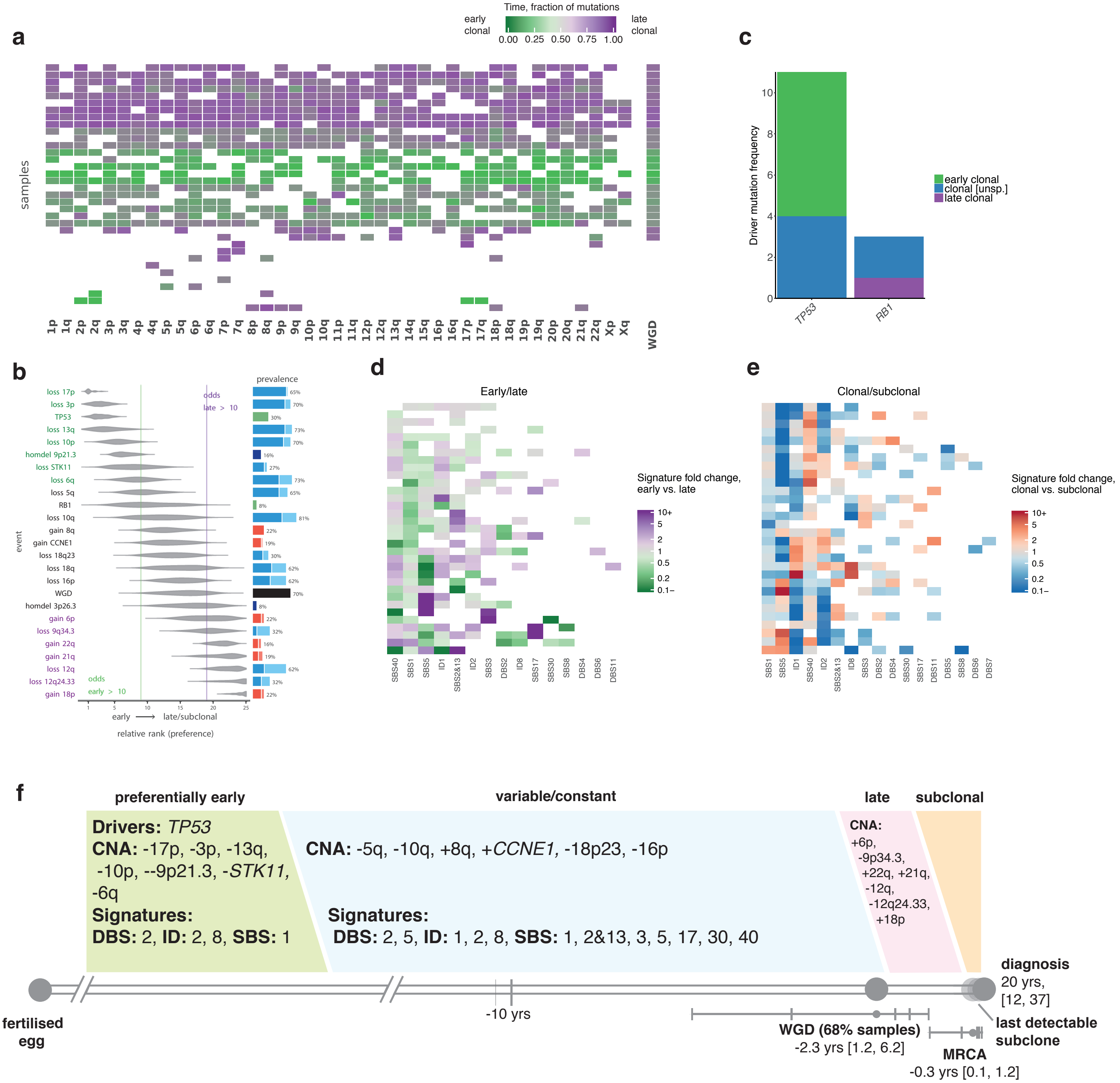

Supplementary Figure 26. Summary of all results for osteosarcoma (*n*=38). a, Clustered heatmaps of mutational timing estimates for gained segments, per patient. Colours as indicated in main text: green represents early clonal events, purple represents late clonal. **b**, Relative ordering of copy number events and driver mutations across all samples. **c**, Distribution of mutations across early clonal, late clonal and subclonal stages, for the most common driver genes. A maximum of 10 driver genes are shown. **d**, Clustered mutational signature fold changes between early clonal and late clonal stages, per patient. Green and purple indicate, respectively, a signature decrease and increase in late clonal from early clonal mutations. Inactive signatures are coloured white. **e**, As in **d** but for clonal versus subclonal stages. Blue indicates a signature decrease and red an increase in subclonal from clonal mutations. **f**, Typical timeline of tumour development.

Ovarian adenocarcinoma

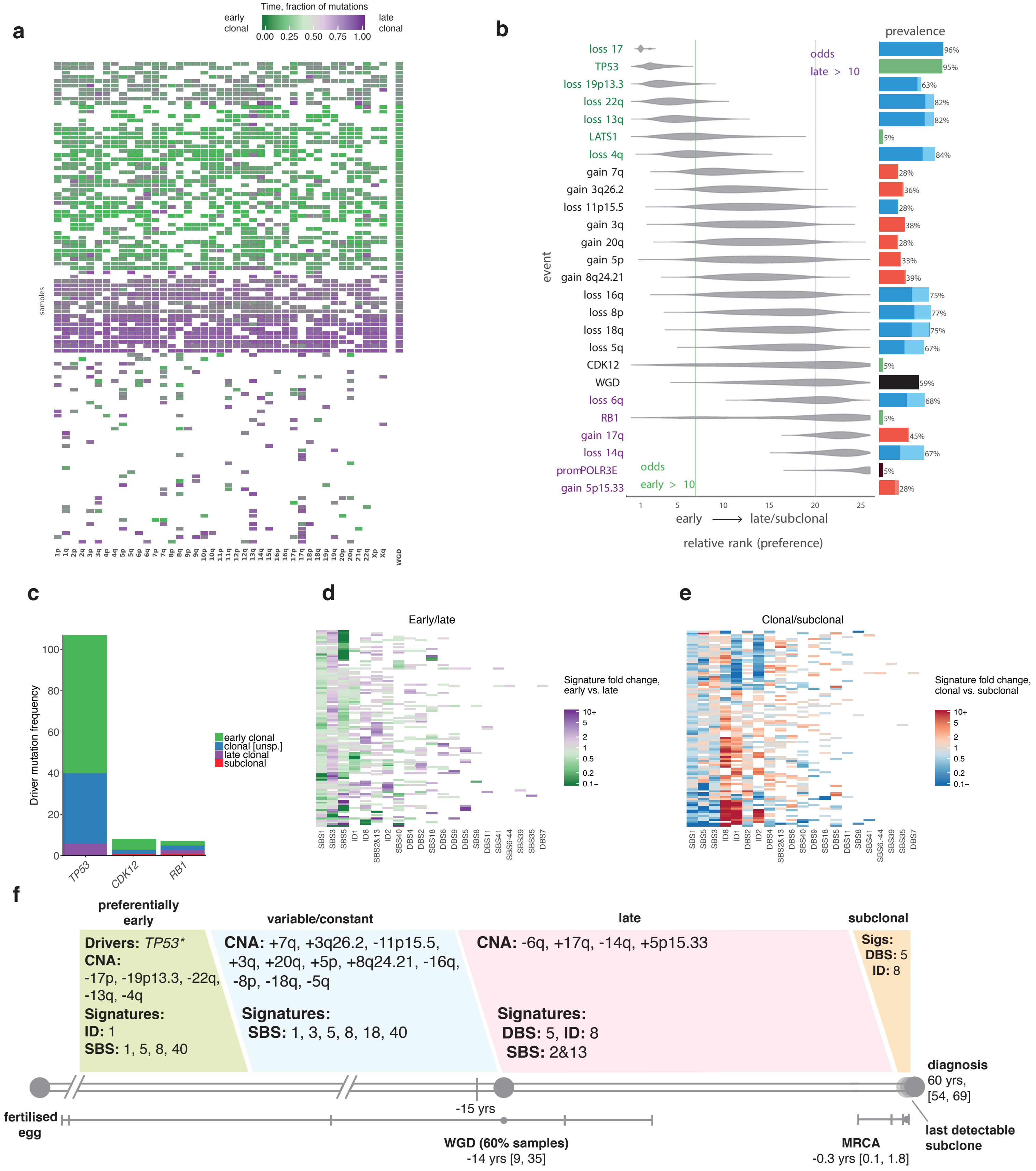

Supplementary Figure 27. Summary of all results obtained for ovarian adenocarcinoma (*n*=113). **a**, Clustered heatmaps of mutational timing estimates for gained segments, per patient. Colours as indicated in main text: green represents early clonal events, purple represents late clonal. **b**, Relative ordering of copy number events and driver mutations across all samples. **c**, Distribution of mutations across early clonal, late clonal and subclonal stages, for the most common driver genes. A maximum of 10 driver genes are shown. **d**, Clustered mutational signature fold changes between early clonal and late clonal stages, per patient. Green and purple indicate, respectively, a signature decrease and increase in late clonal from early clonal mutations. Inactive signatures are coloured white. **e**, As in **d** but for clonal versus subclonal stages. Blue indicates a signature decrease and red an increase in subclonal from clonal mutations. **f**, Typical timeline of tumour development.

Pancreatic adenocarcinoma

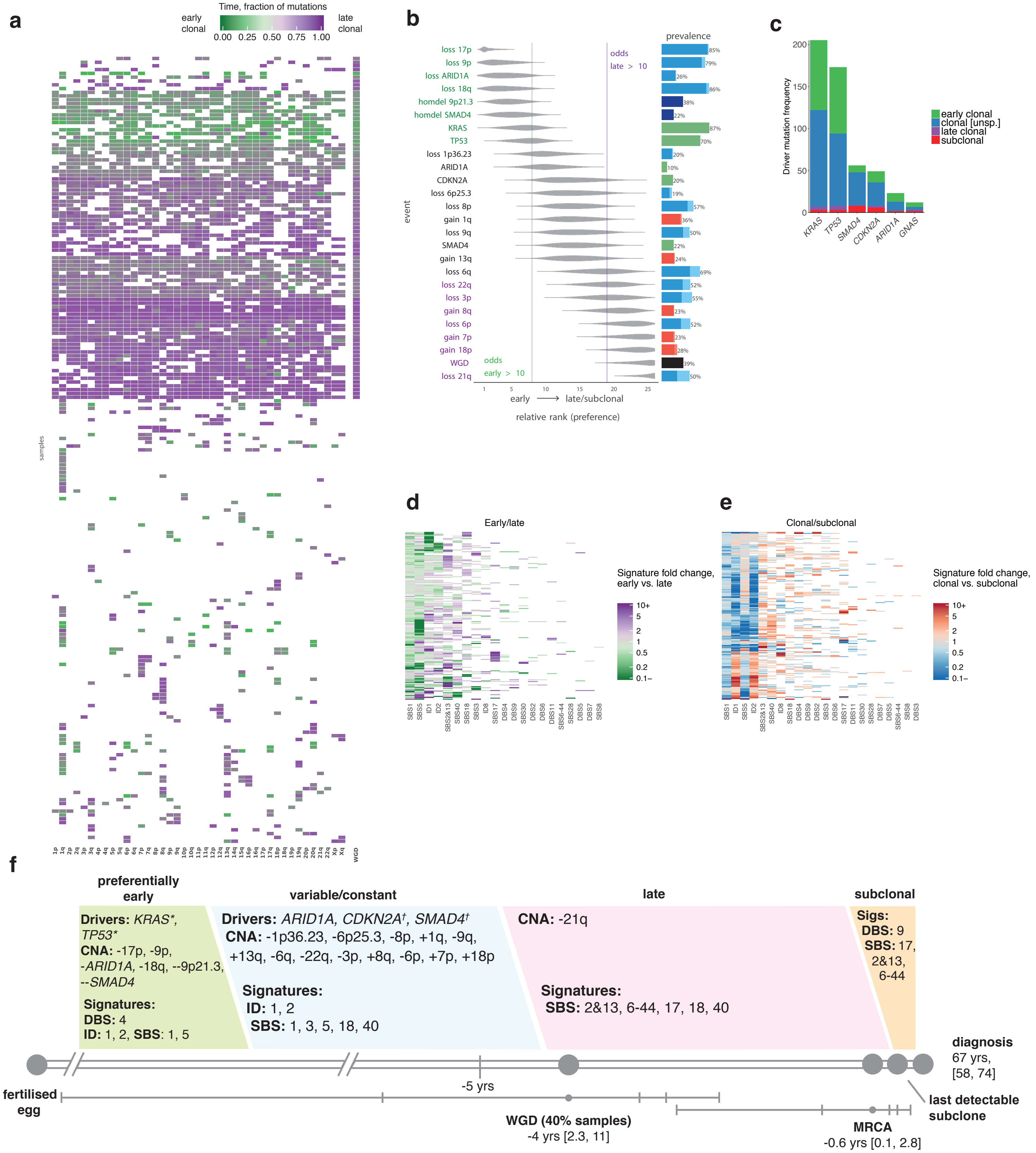

Supplementary Figure 28. Summary of all results obtained for pancreatic adenocarcinoma (*n*=241). a, Clustered heatmaps of mutational timing estimates for gained segments, per patient. Colours as indicated in main text: green represents early clonal events, purple represents late clonal. **b**, Relative ordering of copy number events and driver mutations across all samples. **c**, Distribution of mutations across early clonal, late clonal and subclonal stages, for the most common driver genes. A maximum of 10 driver genes are shown. **d**, Clustered mutational signature fold changes between early clonal and late clonal stages, per patient. Green and purple indicate, respectively, a signature decrease and increase in late clonal from early clonal mutations. Inactive signatures are coloured white. **e**, As in **d** but for clonal versus subclonal stages. Blue indicates a signature decrease and red an increase in subclonal from clonal mutations. **f**, Typical timeline of tumour development.

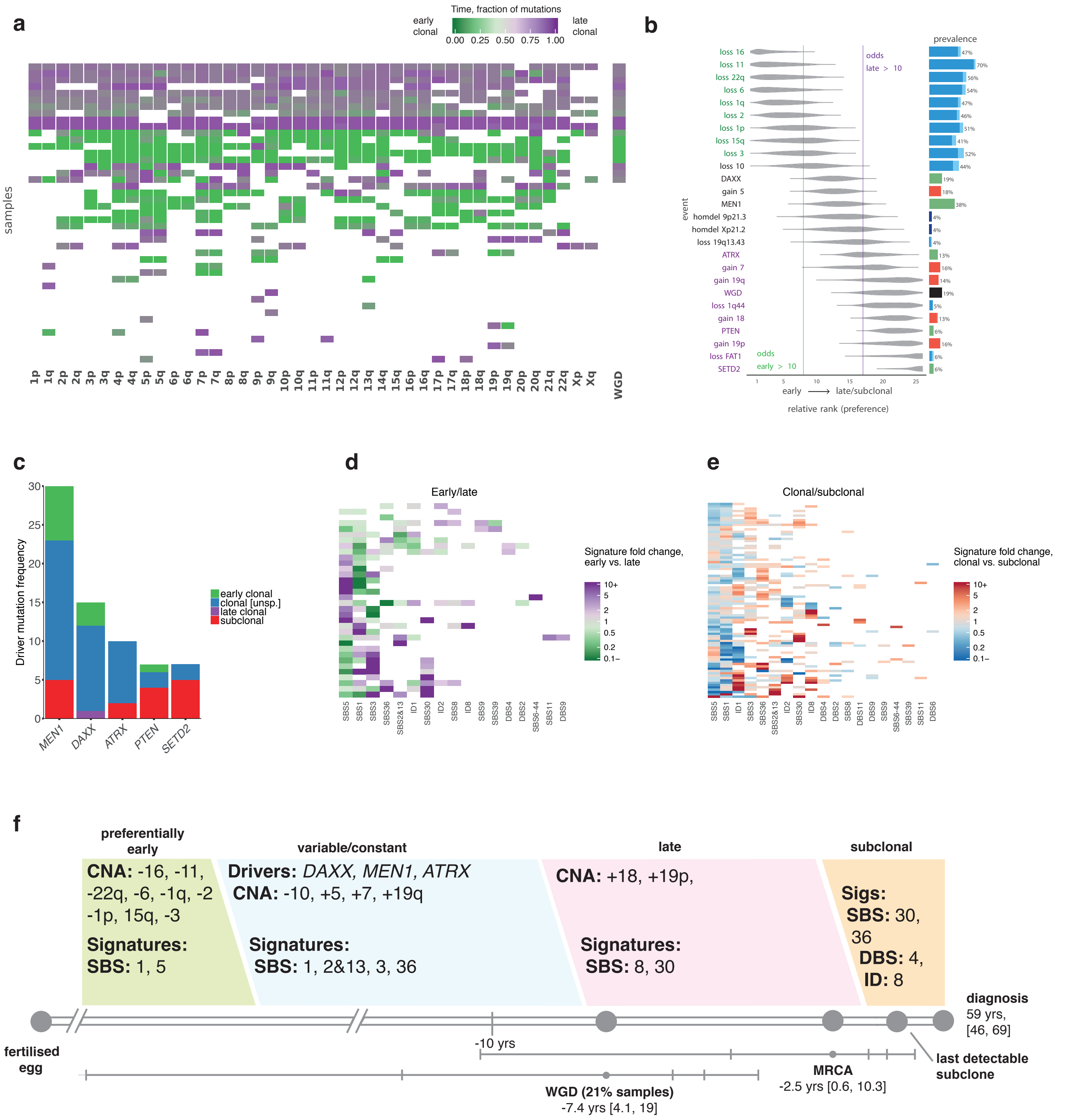

Supplementary Figure 29. Summary of all results obtained for pancreatic neuroendocrine tumours (*n*=85). a, Clustered heatmaps of mutational timing estimates for gained segments, per patient. Colours as indicated in main text: green represents early clonal events, purple represents late clonal. **b**, Relative ordering of copy number events and driver mutations across all samples. **c**, Distribution of mutations across early clonal, late clonal and subclonal stages, for the most common driver genes. A maximum of 10 driver genes are shown. **d**, Clustered mutational signature fold changes between early clonal and late clonal stages, per patient. Green and purple indicate, respectively, a signature decrease and increase in late clonal from early clonal mutations. Inactive signatures are coloured white. **e**, As in **d** but for clonal versus subclonal stages. Blue indicates a signature decrease and red an increase in subclonal from clonal mutations. **f**, Typical timeline of tumour development.

Papillary renal cell carcinoma

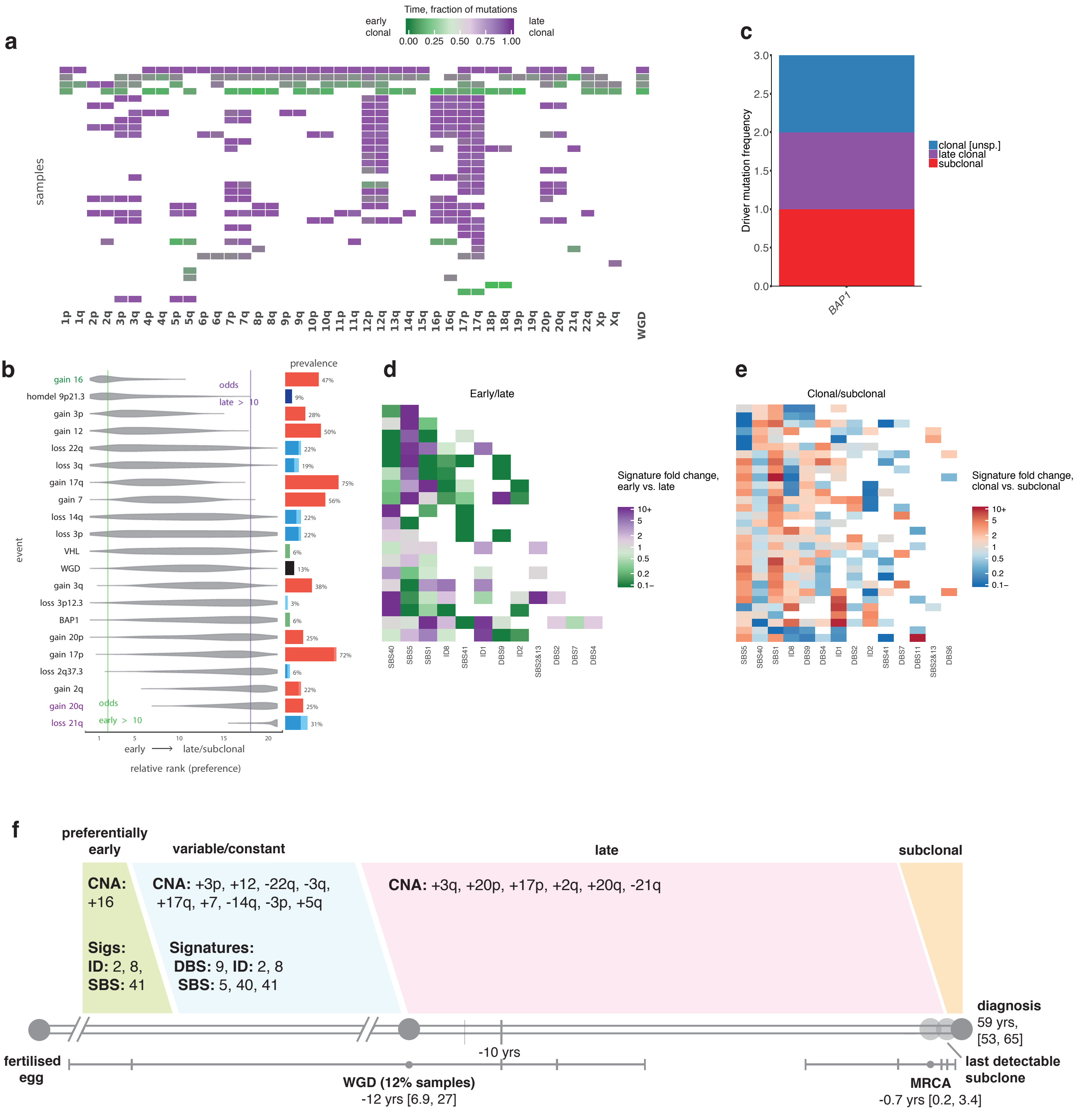

Supplementary Figure 30. Summary of all results obtained for papillary renal cell carcinoma (*n*=33). a, Clustered heatmaps of mutational timing estimates for gained segments, per patient. Colours as indicated in main text: green represents early clonal events, purple represents late clonal. **b**, Relative ordering of copy number events and driver mutations across all samples. **c**, Distribution of mutations across early clonal, late clonal and subclonal stages, for the most common driver genes. A maximum of 10 driver genes are shown. **d**, Clustered mutational signature fold changes between early clonal and late clonal stages, per patient. Green and purple indicate, respectively, a signature decrease and increase in late clonal from early clonal mutations. Inactive signatures are coloured white. **e**, As in **d** but for clonal versus subclonal stages. Blue indicates a signature decrease and red an increase in subclonal from clonal mutations. **f**, Typical timeline of tumour development.

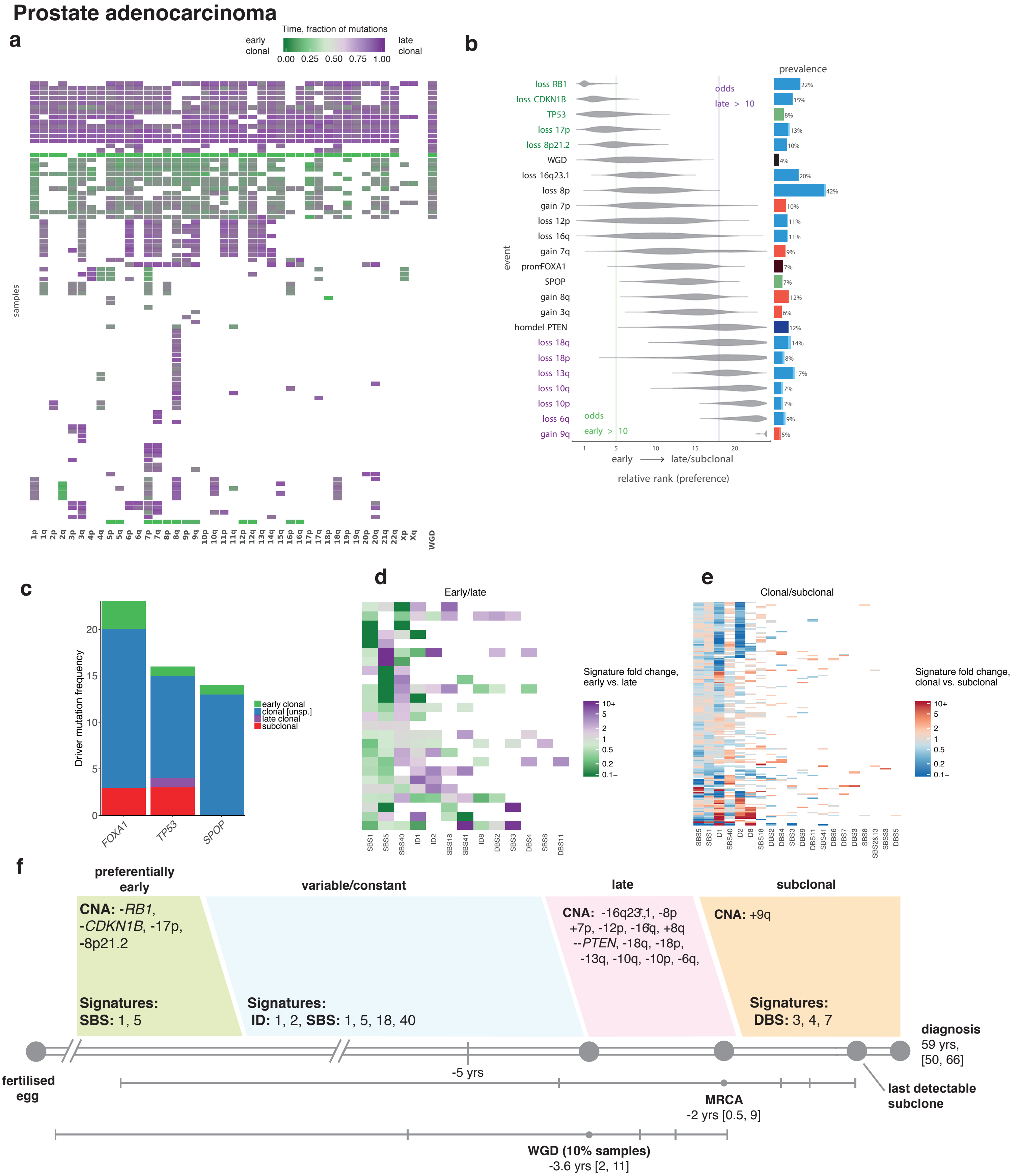

Supplementary Figure 31. Summary of all results obtained for prostate adenocarcinoma (*n*=286). a, Clustered heatmaps of mutational timing estimates for gained segments, per patient. Colours as indicated in main text: green represents early clonal events, purple represents late clonal. **b**, Relative ordering of copy number events and driver mutations across all samples. **c**, Distribution of mutations across early clonal, late clonal and subclonal stages, for the most common driver genes. A maximum of 10 driver genes are shown. **d**, Clustered mutational signature fold changes between early clonal and late clonal stages, per patient. Green and purple indicate, respectively, a signature decrease and increase in late clonal from early clonal mutations. Inactive signatures are coloured white. **e**, As in **d** but for clonal versus subclonal stages. Blue indicates a signature decrease and red an increase in subclonal from clonal mutations. **f**, Typical timeline of tumour development.

Pilocytic astrocytoma

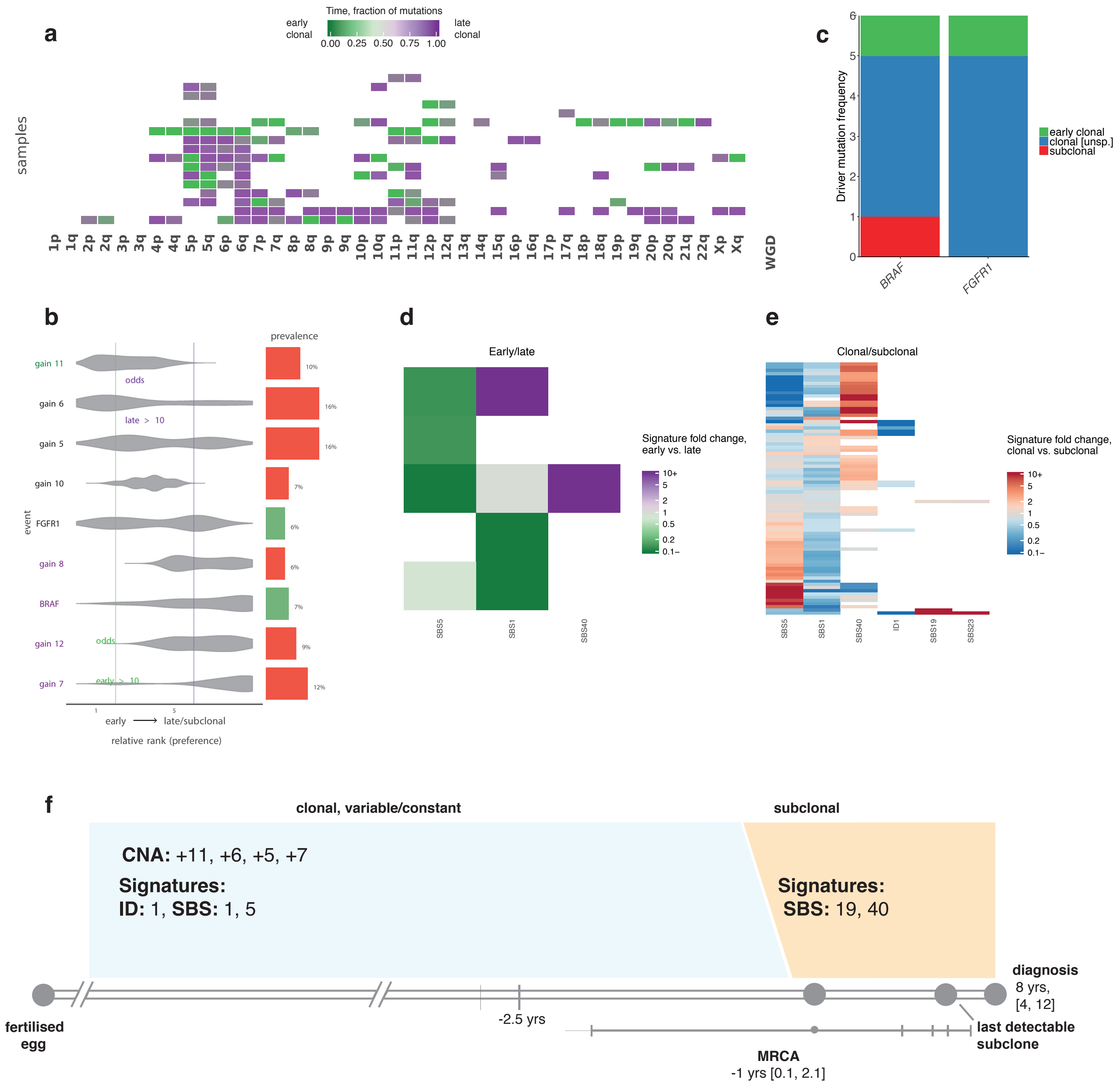

Supplementary Figure 32. Summary of all results obtained for pilocytic astrocytoma (*n*=89). a, Clustered heatmaps of mutational timing estimates for gained segments, per patient. Colours as indicated in main text: green represents early clonal events, purple represents late clonal. **b**, Relative ordering of copy number events and driver mutations across all samples. **c**, Distribution of mutations across early clonal, late clonal and subclonal stages, for the most common driver genes. A maximum of 10 driver genes are shown. **d**, Clustered mutational signature fold changes between early clonal and late clonal stages, per patient. Green and purple indicate, respectively, a signature decrease and increase in late clonal from early clonal mutations. Inactive signatures are coloured white. **e**, As in **d** but for clonal versus subclonal stages. Blue indicates a signature decrease and red an increase in subclonal from clonal mutations. **f**, Typical timeline of tumour development.

Squamous cell cervical cancer

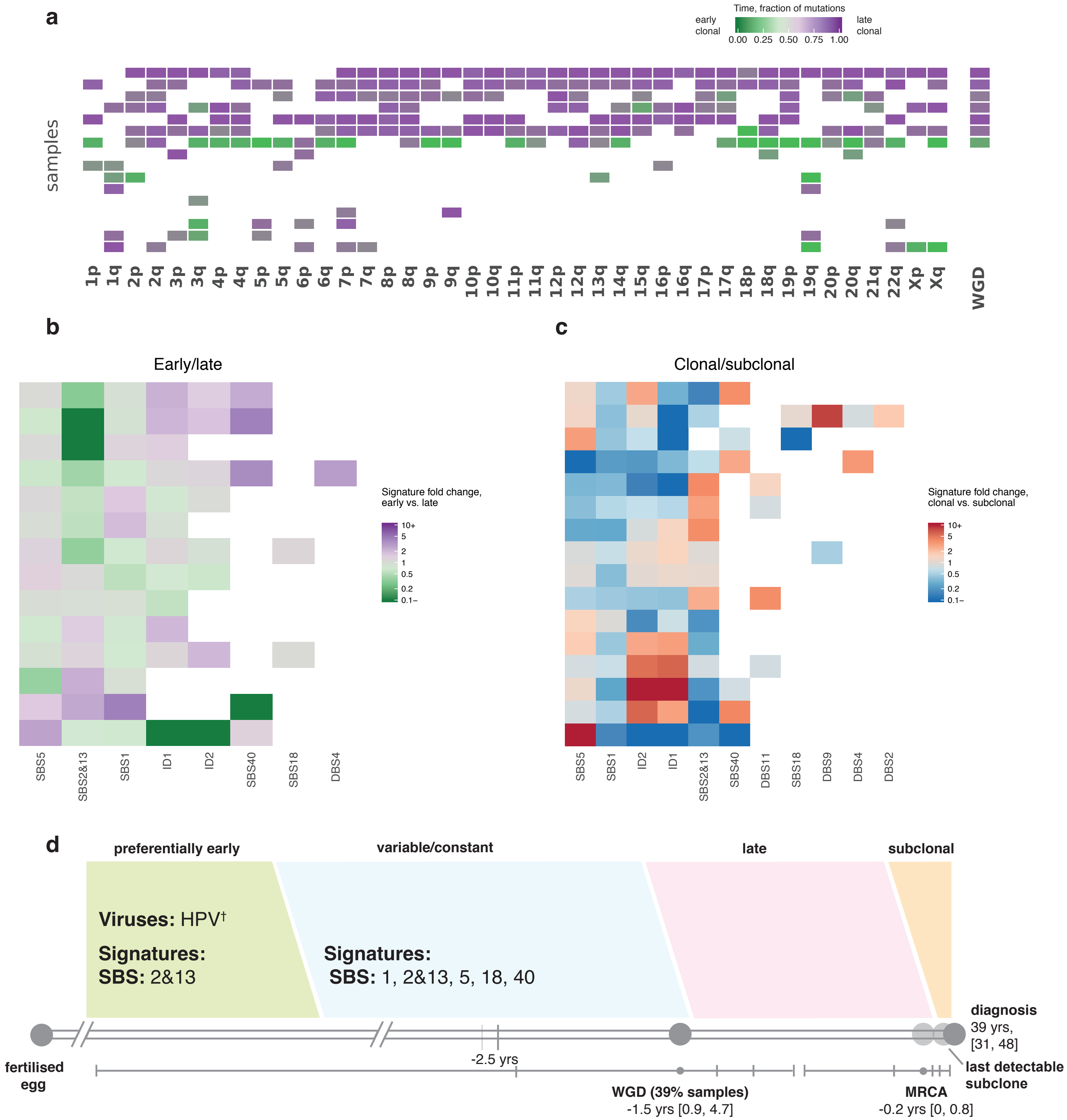

Supplementary Figure 33. Summary of all results obtained for squamous cell cervical cancer (*n*=18). a, Clustered heatmaps of mutational timing estimates for gained segments, per patient. Colours as indicated in main text: green represents early clonal events, purple represents late clonal. **b**, Clustered mutational signature fold changes between early clonal and late clonal stages, per patient. Green and purple indicate, respectively, a signature decrease and increase in late clonal from early clonal mutations. Inactive signatures are coloured white. **c**, As in **b** but for clonal versus subclonal stages. Blue indicates a signature decrease and red an increase in subclonal from clonal mutations. **d**, Typical timeline of tumour development.

Squamous cell lung cancer

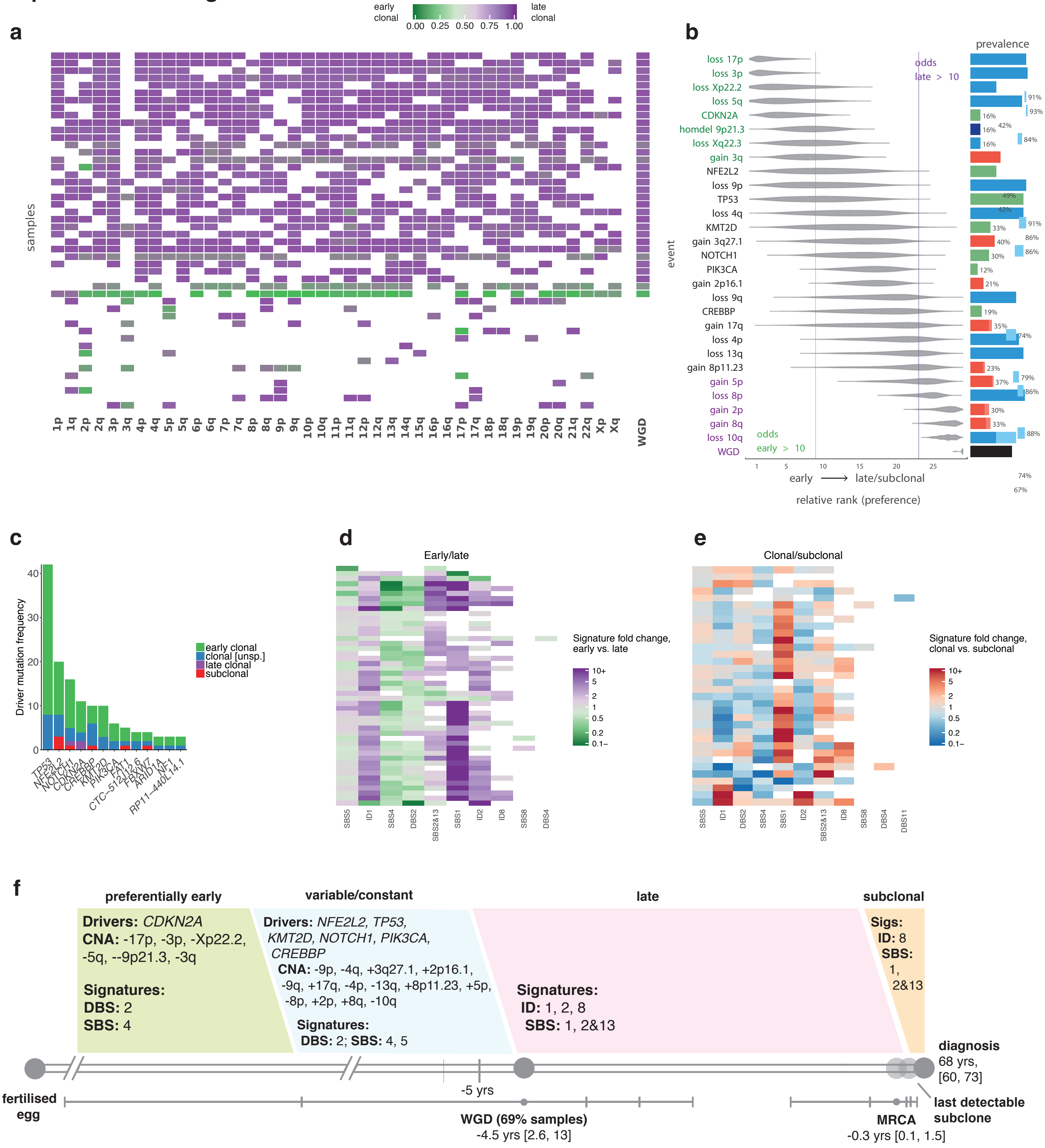

Supplementary Figure 34. Summary of all results obtained for squamous cell lung cancer (*n*=48). a, Clustered heatmaps of mutational timing estimates for gained segments, per patient. Colours as indicated in main text: green represents early clonal events, purple represents late clonal. **b**, Relative ordering of copy number events and driver mutations across all samples. **c**, Distribution of mutations across early clonal, late clonal and subclonal stages, for the most common driver genes. A maximum of 10 driver genes are shown. **d**, Clustered mutational signature fold changes between early clonal and late clonal stages, per patient. Green and purple indicate, respectively, a signature decrease and increase in late clonal from early clonal mutations. Inactive signatures are coloured white. **e**, As in **d** but for clonal versus subclonal stages. Blue indicates a signature decrease and red an increase in subclonal from clonal mutations. **f**, Typical timeline of tumour development.

Thyroid adenocarcinoma

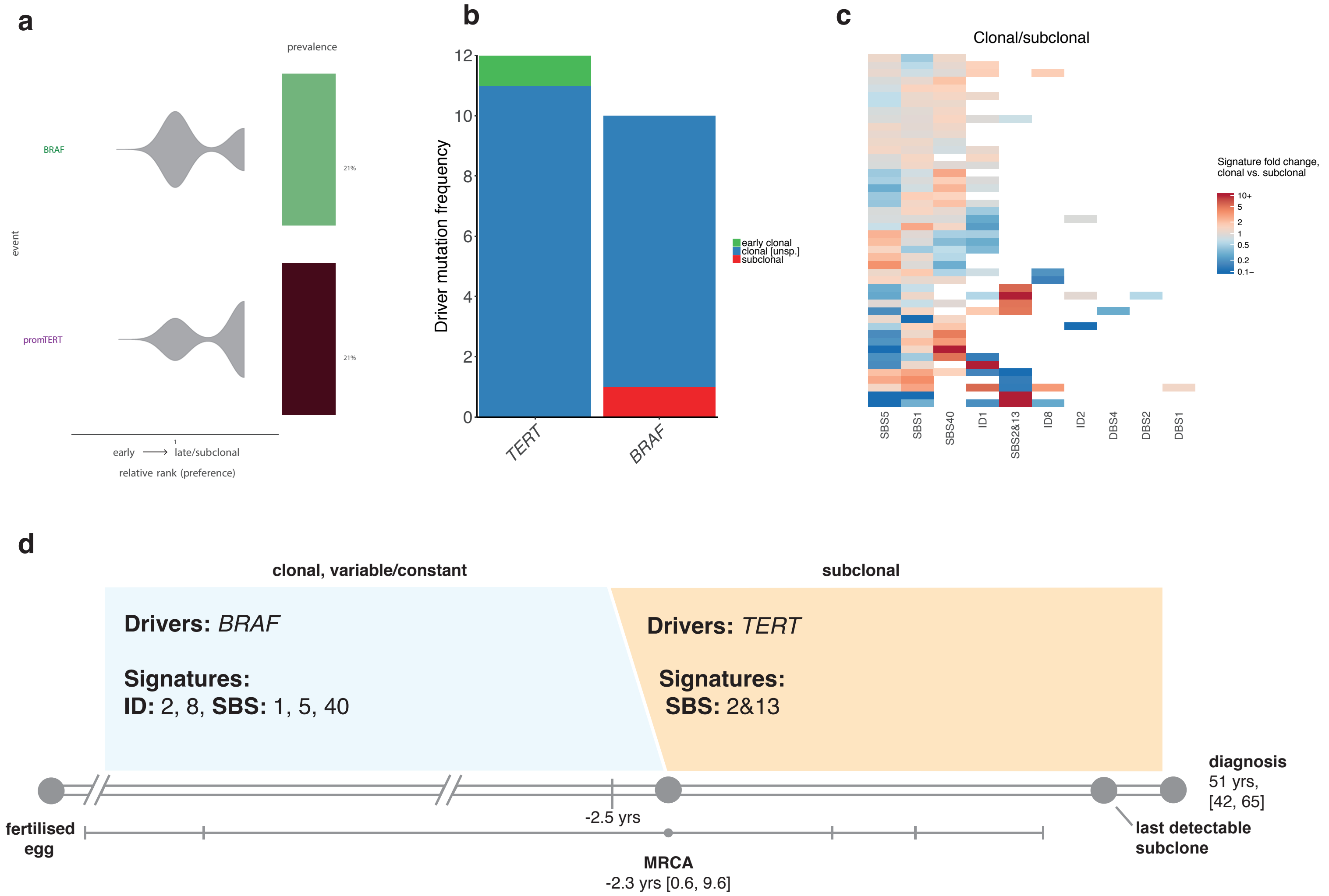

Supplementary Figure 35. Summary of all results obtained for thyroid adenocarcinoma (*n*=48). **a**, Relative ordering of copy number events and driver mutations across all samples. **b**, Distribution of mutations across early clonal, late clonal and subclonal stages, for the most common driver genes. A maximum of 10 driver genes are shown. **c**, Clustered mutational signature fold changes between clonal and subclonal stages, per patient. Blue indicates a signature decrease and red an increase in subclonal from clonal mutations. **d**, Typical timeline of tumour development.

Transitional cell bladder cancer

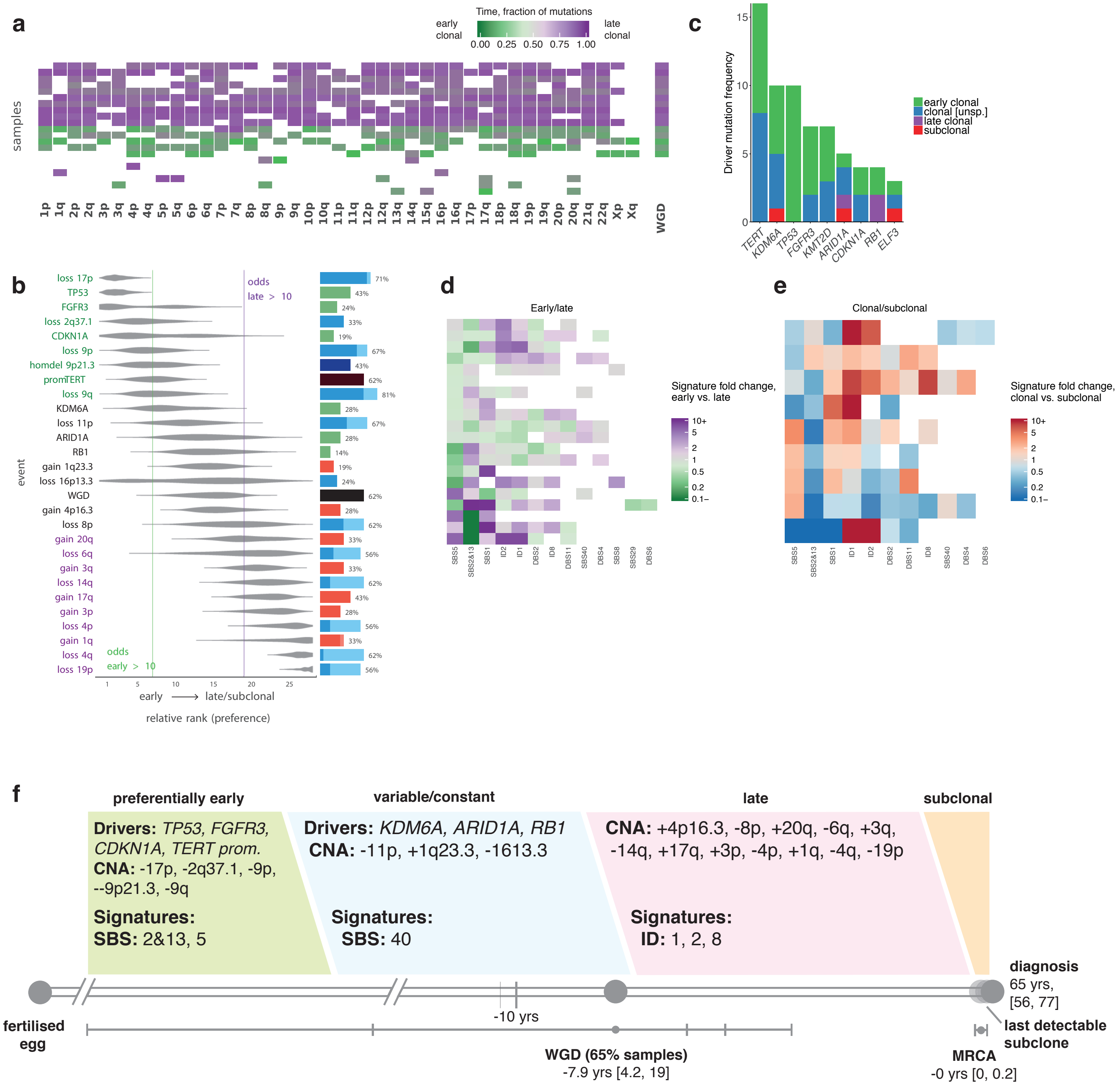

Supplementary Figure 36. Summary of all results for transitional cell bladder cancer (*n*=23). a, Clustered heatmaps of mutational timing estimates for gained segments, per patient. Colours as indicated in main text: green represents early clonal events, purple represents late clonal. **b**, Relative ordering of copy number events and driver mutations across all samples. **c**, Distribution of mutations across early clonal, late clonal and subclonal stages, for the most common driver genes. A maximum of 10 driver genes are shown. **d**, Clustered mutational signature fold changes between early clonal and late clonal stages, per patient. Green and purple indicate, respectively, a signature decrease and increase in late clonal from early clonal mutations. Inactive signatures are coloured white. **e**, As in **d** but for clonal versus subclonal stages. Blue indicates a signature decrease and red an increase in subclonal from clonal mutations. **f**, Typical timeline of tumour development.
